# Airway registries in primarily adult, emergent endotracheal intubation: a scoping review

**DOI:** 10.1186/s13049-023-01075-z

**Published:** 2023-03-08

**Authors:** Sarah Meulendyks, Daniel Korpal, Helen Jingshu Jin, Sameer Mal, Jacob Pace

**Affiliations:** 1 Schulich School of Medicine and Dentistry, 1151 Richmond St, London, ON N6A 5C1 Canada; 2 Department of Emergency Medicine, London Health Sciences Centre, 800 Commissioners Rd E, London, ON N6A 5W9 Canada

**Keywords:** Airway registry, Endotracheal intubation, Emergency department, Emergency medicine, Airway management

## Abstract

**Background:**

Emergency Department (ED) airway registries are formalized methods to collect and document airway practices and outcomes. Airway registries have become increasingly common in EDs globally; yet there is no consensus of airway registry methodology or intended utility. This review builds on previous literature and aims to provide a thorough description of international ED airway registries and discuss how airway registry data is utilized.

**Methods:**

A search of Medline, Embase, Scopus, Cochrane Libraries, Web of Science, and Google Scholar was performed with no date limitations applied. English language full-text publications and grey literature from centres implementing an ongoing airway registry to monitor intubations performed in mainly adult patients in an ED setting were included. Non-English publications and publications describing airway registries to monitor intubation practices in predominantly paediatric patients or settings outside of the ED were excluded. Study screening for eligibility was performed by two team members individually, with any disagreements resolved by a third team member. Data was charted using a standardized data charting tool created for this review.

**Results:**

Our review identified 124 eligible studies from 22 airway registries with a global distribution. We found that airway registry data is used for quality assurance, quality improvement, and clinical research regarding intubation practices and contextual factors. This review also demonstrates that there is a great deal of heterogeneity in definitions of first-pass success and adverse events in the peri-intubation period.

**Conclusions:**

Airway registries are used as a crucial tool to monitor and improve intubation performance and patient care. ED airway registries inform and document the efficacy of quality improvement initiatives to improve intubation performance in EDs globally. Standardized definitions of first-pass success and peri-intubation adverse events, such as hypotension and hypoxia, may allow for airway management performance to be compared on a more equivalent basis and allow for the development of more reliable international benchmarks for first-pass success and rates of adverse events in the future.

**Supplementary Information:**

The online version contains supplementary material available at 10.1186/s13049-023-01075-z.

## Introduction

Emergent endotracheal intubation is a core skill for Emergency Medicine (EM) physicians, is performed frequently in Emergency Departments (EDs) globally, and is a complex and challenging task in the care of critically ill patients [1]. Complications include failed first attempts, the need to perform a surgical airway, and other life-threatening adverse events such as hypotension, hypoxemia, aspiration, dysrhythmias, and cardiac arrest [2–7]. Many EDs have implemented airway registries to capture endotracheal intubation practices and monitor the frequency of adverse events, with the goal of optimizing patient outcomes [1, 2, 8]. Airway registries have been implemented in many regions including Australia and New Zealand [9–11], North America [12–18], Europe [19, 20], Asia [21–24], and Africa [25].

Airway registries offer significant potential utility as sources of data for quality assurance (QA), quality improvement (QI), and clinical research [2, 23, 25–27]. Despite the growing body of research involving airway registries, there is no consensus of their methodology, how airway registry data is utilized, or their definitions of key performance indicators of intubation. One review exploring this topic identified this problem but did not assess the full scope of airway registries as critical grey literature sources were not included [28].

We performed a scoping review to answer three important questions regarding airway registries. The primary objective is to describe the current scope and prevalence of ED airway registries globally. Secondary objectives include describing utilization of airway registry data and determining how various airway registries define adverse events and key performance indicators during the peri-intubation period.

## Methods

A scoping review was completed using the Preferred Reporting Items for Systematic Reviews and Meta-Analysis Extension for Scoping Reviews (PRISMA-ScR) framework [29].

### Eligibility criteria

We included all published studies (full-text publications, conference abstracts, conference posters) which reported the implementation of an airway registry to monitor intubation practices among adult patients within an ED. We defined an airway registry as a formal process implemented within the ED to monitor intubation practices by recording information pertinent to intubation and adverse events independent from a finite intervention or research hypothesis. Non-English publications, studies that reported greater than 50% of their intubations were performed in paediatric patients, studies that did not utilize an airway registry for data collection, or those that included intubations performed primarily outside of the ED (intensive care unit [ICU], operating theatre, pre-hospital, in-patient wards) were excluded. No date limitations were applied.

### Information sources and search

A search conducted in Ovid Medline (1946–2021), Embase (1947–2021), Scopus (2021), Cochrane Libraries (2021), Web of Science (2021), and Google Scholar (2021) was initially completed on November 3, 2021. A systematic search strategy was developed and translated into each of the databases’ syntax. The full search strategy for all databases is shown in Additional file 1. The intent of this search was to identify all peer-reviewed and grey literature pertaining to ED airway registries. The search of all databases followed by study screening was repeated on July 26, 2022, with a date limitation applied (November 3, 2021–July 26, 2022).

### Study selection

Studies identified were imported to Covidence, an online platform for screening and organizing data for literature reviews, and automatically de-duplicated. Any subsequent duplicates noted were manually excluded by authors collaboratively throughout the review process. An initial review of abstracts was completed using the aforementioned eligibility criteria. Full texts (if available) were reviewed for eligibility by two reviewers, independently. Discrepancies were resolved by a third team member.

### Data charting

Data was charted by two independent reviewers (SM and HJ) using a standardized data charting form created in Microsoft Excel (2018) [30] specifically for this review. The data charting form was piloted for usability by two reviewers for 5 studies each prior to data charting. Charted study information was informed by the Arksey and O’Malley Framework for Scoping Reviews [31]. Additional Files 2 through 5 depict the totality of all data charted for each included publication.

### Synthesis of results

The total number of patients for each registry was reported as the largest number of patients available in identified publications within each registry. Years active was determined based on time periods of data collection for all included studies in each registry. To be as representative of current trends as possible, data was reported from the most recent, full-text publication (if available), within each registry. If the most recent publication was missing any of this information, it was reported from the next most recent publication, and so on.

To examine the utilization of airway registry data, studies were grouped into research or QA/QI. For the purposes of this review, QA was defined as studies that measure compliance against certain required standards [32]. QI was defined as studies implementing a proactive approach to improve specific processes or systems [33]. Utilization of airway registry data from all publications was synthesized.

## Results

**Fig. 1:**
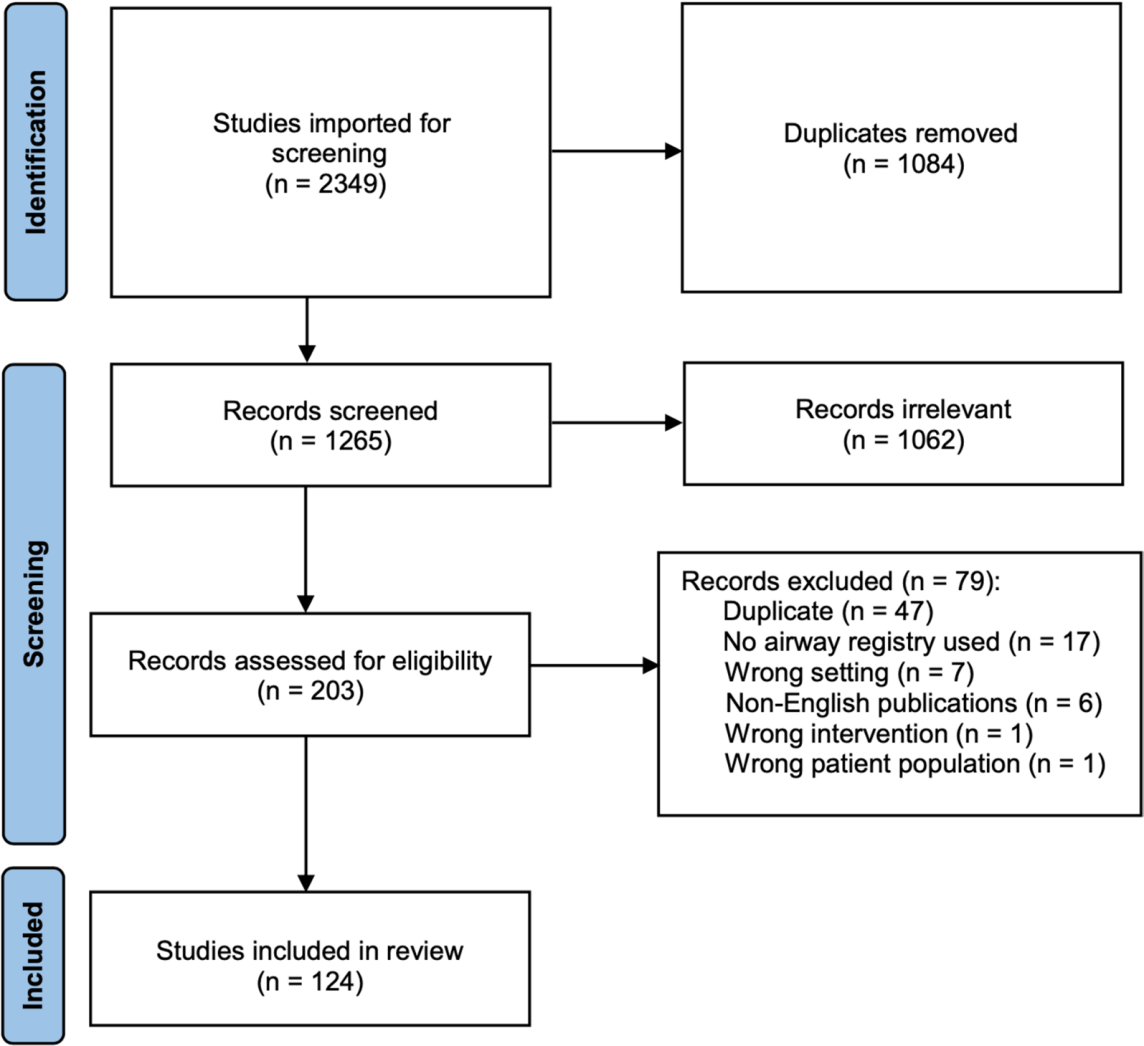
PRISMA Flow diagram for study inclusion

The combined searches yielded 2349 results, including 1084 duplicates. Following title/abstract and full-text screening, 124 studies were included in our review consisting of 29 conference abstracts, one conference poster, and 94 full-text, peer-reviewed publications, as illustrated in Fig. 1. Included studies were published between the years of 1999 and 2022.

### Characteristics of identified airway registries

A summary of characteristics of identified airway registries is provided in Table 1. A complete list of the characteristics for each included publication can be found in Additional File 2. A total of 22 airway registries were identified globally with seven registries in North America, seven registries in Asia, four registries in Australia and New Zealand, three registries in Europe, and one registry in Africa. Of the registries identified, nine of 22 have only a single full-text publication, conference abstract, or poster available. Nine of 22 registries provide data from multiple centres ranging from three centres in the British Columbia Airway Registry for Emergencies (BCARE) to 43 centres reported in the Australian and New Zealand Emergency Department Airway Registry (ANZEDAR) [1, 34]. The largest and longest running dataset is the National Emergency Airway Registry (NEAR) which exists as three iterations reporting on intubation practices across four countries (USA, Canada, Singapore, and Australia) from 1996 to 2018. NEAR includes a total of 29, 296 ED intubations between NEARI, NEARII, and NEARIII reporting 1288, 8937, and 19,071 intubations, respectively [12–14].

**Table 1:** Characteristics of airway registries

Name of Airway Registry	Geographic Location	Number of Participating Centres	Total number of patients reported *	Number of Publications Included	Years Active	Location of Intubations	Age (% paediatric patients included)	Most Representative Publication**
ANZEDAR	Australia, New Zealand	43	5063	8 (7 full-text, 1 abstract)	2010- 2019	ED only	All patients (4.8% aged 0–15)	Alkhouri et al., 2017
BCARE	Canada	3	737	3 (3 abstracts)	2017- 2019	ED (53.7%), ICU (30%), ward (16.3%)	17 years or older	Yoo et al., 2018
Chulalongkorn Airway Registry	Bangkok, Thailand	1	226	1 (full-text)	2017- 2018	ED only	18 years or older	Saoraya et al., 2021
Cipto Mangunkusumo General Hospital Airway Registry	Jakarta, Indonesia	1	231	1 (full-text)	2018- 2019	ED only	All ages (8.7%)	Sulistio et al., 2021
Cleveland Clinic Emergency Airway Registry	Cleveland, USA	1	961	4 (3 full-text, 1 abstract)	2005—2010	ED (70%) and prehospital (30%)	All ages	Phelan et al., 2016
Continuous Quality Improvement Database	Tucson, USA	1	5229	23 (18 full text, 5 abstracts)	2007—2018	ED only	18 years or older	Sakles et al., 2019
DREAM	San Antonio, USA	1	74	1 (full-text)	2020	ED only	18 years or older	Mendez et al., 2021
EDIR	Edinburgh, UK	7	3988	7 (7 full-text)	1999- 2011	ED only	All ages (0.2% aged < 13 years)	Hale et al., 2017
JEANI + II	Japan	14	10,927	7 (7 full-text)	2010—2016	ED only	All ages (3% aged 0–17)	Goto et al., 2017
KEAMR	South Korea	20	10,978	7 (6 full-text, 1 abstract)	2006—2012	ED only	All ages	Kim et al., 2017
King Abdulaziz University Hospital Airway Registry	Jeddah, Saudi Arabia	1	146	1(full-text)	2018- 2020	ED only	All ages	Bakhsh et al., 2021
Middlemore Hospital Airway Registry	Auckland, New Zealand	1	258	1 (poster)	2014	ED only	Not reported	Brainard et al., 2014
NEARI	USA	11	1288	1 (full-text)	1996- 1997	ED only	All ages (13.9% aged 0–18)	Sagarin et al., 2003
NEARII	USA, Canada, Singapore	31	8937	6 (2 abstract, 4 full-text)	1997–2002	ED only	All ages	Walls et al., 2011
NEARIII	USA	25	19,071	36 (12 abstract, 24 full-text)	2002–2018	ED only	> 17 years old	Nikolla et al., 2022
NERAA	Ireland	11	118	1 (1 full-text)	2020	ED only	16 years or older	Umana et al., 2022
Samsung Medical Centre Emergency Airway Program	Seoul, South Korea	1	1087	4 (4 full-text)	2007- 2017	ED only	18 years or older	Hwang, Park, et al., 2018
Singapore General Hospital Emergency Airway Registry	Singapore	1	2950	5 (2 abstract, 3 full-text)	2000–2016	ED only	All ages	Weng et al., 2021
South African Emergency Department Registry	Johannesburg, South Africa	1	374	1 (full-text)	2015- 2016	ED only	18 years or older	Hart and Goldstein., 2020
The Aberdeen Royal Infirmary Airway Registry	Aberdeen, UK	1	197	1 (abstract)	2015- 2017	ED only	All ages	Yeap et al., 2019
The Alfred Airway Registry	Melbourne, Australia	1	783	2 (2 full-text)	2017- 2020	ED only	All ages	Groombridge et al., 2021
The Royal North Shore Emergency Airway Registry	Sydney, Australia	1***	601	3 (1 abstract, 2 full-text)	2010- 2014	ED only	All ages (7% age < 17 years)	Fogg et al., 2016

*ANZEDAR* The Australian and New Zealand Emergency Department Airway Registry, *BCARE* British Columbia Airway Registry for Emergencies, *DREAM* Defense Registry for Emergency Airway Management,

*EDIR* Emergency Department Intubation Registry, *JEAN* Japanese Emergency Airway Network Registry 1 and 2, *KEAMR* Korean Emergency Airway Management Registry, *NEAR* National Emergency Airway Registry, *NERAA* National Emergency Resuscitation Airway Audit

^*^ Largest number of patients available in each registry

^**^Most recent, full-text, multicentre publication (if available) within each registry

^***^One study within this registry included data contributed from a second centre, but it is considered a single centre registry

Seventeen of the 22 registries identified began their data collection in 2005 or later. The earliest time periods of data collection are seen in NEARI, II, and III which began in 1996, 1997, and 2002 respectively [12, 35, 36], Emergency Department Intubation Registry (EDIR) which began in 1999 [37], and the Singapore General Hospital Emergency Airway Registry which began in 2000 [38]. Only two registries included data on intubations that took place outside of the ED setting. The Cleveland Clinic Emergency Airway Registry includes 30% prehospital intubations and BCARE includes 30% ICU intubations and 16.3% ward intubations [34, 39]. Six registries also specify the exact percentage of their data that reflects paediatric intubations, ranging from 0.2% of patients aged 13 or less in EDIR, to 13.9% patients aged zero to 18 in NEARI [12, 19]. This reflects variable definitions of the age which constitutes a paediatric patient across the identified airway registries depending on the region or country of origin.

### Types of publications and utilization of airway registry data

The utilization of airway registry data varied; eight of 22 registries were used in QA studies, eight of 22 for QI studies, and 21 of 22 for research. Note that one publication was classified as both QI and research [10]. Table 2 provides a summary of QA and QI publications identified. Five of 10 QA studies focused on auditing the practices and outcomes of their single local centre by comparing its performance to benchmark data from other centres within the same registry, or from a larger, more established registry. Of the QI publications identified, all but three focused on dedicated QI programs implemented at a local centre. The remaining three studies assessed the impact of procedural changes in response to disease outbreaks (COVID-19 and SARS [10, 38]) or conducted a cross-sectional evaluation of simulation-based training programs between centres [40]. A list of all included QA and QI publications is provided in Additional File 3.

**Table 2:** Utilization of airway registry data—QI/QA studies

Publication Type	Purpose of Study	Number of Studies	Registries Included
QA	Description of airway management practices at a local centre and its comparison to larger centres	5	ANZEDAR NEARIII South African ED Registry The Royal North Shore Emergency Airway Registry
	Examination of success rates, stratified by operator level of experience	1	JEANI + II
	Identification of drug use patterns and compliance to recommended standards	1	NEARI
	Characterization of emergency surgical airway cases	1	ANZEDAR
	Evaluation of changing airway management trends over time	1	JEANI + II
	Identification of documentation rate	1	Cleveland Clinic Emergency Airway Registry
QI	Evaluation of local quality improvement initiatives	7	
	Education	5	The Royal North Shore Emergency Airway Registry Cleveland Clinic Emergency Airway Registry Continuous quality improvement database Samsung Medical Centre Emergency Airway Program The Alfred Airway Registry
	Airway registry creation	4	Cleveland Clinic Emergency Airway Registry King Abdulaziz University Hospital Airway Registry Continuous quality improvement database The Alfred Airway Registry
	Procedure standardization	4	The Royal North Shore Emergency Airway Registry Cleveland Clinic Emergency Airway Registry Samsung Medical Centre Emergency Airway Program The Alfred Airway Registry
	Performance target implementation	3	Cleveland Clinic Emergency Airway Registry? Samsung Medical Centre Emergency Airway Program? The Alfred Airway Registry
	Equipment/supplies pre-preparation	1	Samsung Medical Centre Emergency Airway Program
	Impact determination of outbreak-response measures at a local centres	2	Singapore General Hospital Emergency Airway Registry The Alfred Airway Registry
	Evaluation of training programs between centres	1	KEAMR

*ANZEDAR* The Australian and New Zealand Emergency Department Airway Registry, *BCARE* British Columbia Airway Registry for Emergencies, *JEAN* Japanese Emergency Airway Network Registry 1 and 2, *KEAMR* Korean Emergency Airway Management Registry, *NEAR* National Emergency Airway Registry

The most common areas of focus amongst the research-based publications include 34 studies comparing between laryngoscope types, 15 regarding evaluations of medication choice, dosing, and outcomes, and 12 reporting summaries of captured airway management statistics as seen in Table 3. Of the 105 research publications identified, 73 were retrospective analyses and 29 were prospective observational studies. One study used a combination of retrospective analyses and qualitative surveys [41], while two abstracts did not specify their study methodology [34, 42]. A complete list of research publications included can be found in Additional file 4.

**Table 3:** Utilization of airway registry data—Research studies

Purpose of Study	Number of Studies	Registries Included
Evaluate the use and performance of various laryngoscopes	34	Cleveland Clinic Emergency Airway Registry Continuous quality improvement database KEAMR NEARIII Samsung Medical Centre Emergency Airway Program Singapore General Hospital Emergency Airway Registry The Royal North Shore Hospital Emergency Department Airway Registry
Evaluation of drug use and its associated outcomes	15	ANZEDAR Continuous quality improvement database EDIR NEARIII Samsung Medical Centre Emergency Airway Program The Aberdeen Royal Infirmary Airway Registry
General description of local airway management practices and outcomes	12	ANZEDAR Cipto Mangunkusumo General Hospital Airway Registry DREAM EDIR NEARII NEARIII NERAA
Description of airway management practices and outcomes for a specific patient subset (diagnosis)	7	JEANI + II KEAMR NEARII NEARIII
Examination of multiple intubation attempts	5	ANZEDAR Continuous quality improvement database JEANI + II KEAMR
Description of airway registry creation and implementation	4	ANZEDAR BCARE Middlemore Hospital Airway Registry
Description of local airway management practices and outcomes for a specific patient subset (demographics)	4	Continuous quality improvement database EDIR JEANI + II KEAMR
Evaluation of pre-oxygenation use and its associated outcomes	3	ANZEDAR Continuous quality improvement database
Evaluation of performance between operator specialty and/or level of experience	3	BCARE EDIR Singapore General Hospital Emergency Airway Registry
Description of airway management practices and outcomes for difficult airways	3	Chulalongkorn Airway Registry Continuous quality improvement database Singapore General Hospital Emergency Airway Registry
Evaluation of the incidence of adverse events	3	Continuous quality improvement database NEARIII Samsung Medical Centre Emergency Airway Program
Description of surgical airway cases	2	EDIR NEARIII
Examination of intubation method used	2	JEANI + II NEARIII
Examination of patient positioning used	2	NEARIII
Evaluation of the required incidence, use, and outcomes of rescue maneuvers	2	NEARII
Evaluation of end-tidal carbon dioxide monitoring use and its associated outcomes	1	NEARI
Evaluation of bougie use and its associated outcomes	1	NEARIII
Description of airway management cases involving telemedicine	1	NEARIII
Evaluation of implemented COVID-19 procedures	1	The Alfred Airway Registry

*ANZEDAR* The Australian and New Zealand Emergency Department Airway Registry, *BCARE* British Columbia Airway Registry for Emergencies, *DREAM* Defense Registry for Emergency Airway Management,

*EDIR* Emergency Department Intubation Registry, *JEAN* Japanese Emergency Airway Network Registry 1 and 2, *KEAMR* Korean Emergency Airway Management Registry, *NEAR* National Emergency Airway Registry, *NERAA* National Emergency Resuscitation Airway Audit

### Intubation practices and adverse events of identified airway registries

Intubation practices of various airway registries are summarized in Table 4. A complete list of all publications’ intubation practices and adverse events can be found in Additional File 5. EM physicians are reported as the most common intubator in 19 of 22 airway registries. Of these registries, 12 reported that the most common intubator is EM residents. One registry, the Cipto Mangunkusumo General Hospital Airway Registry, reported Anesthetists as the most common intubator.

**Table 4:** Intubation practices in identified airway registries

Name of Airway Registry	Most Common Intubator(s) (% of intubations)	Most Common Indication for Intubation (% of intubations) *	FPS (%)	Most common device (% of intubations)	Medications (% of intubations)	Rates of adverse events (% of patients)	Most common adverse event (% of patients)
ANZEDAR	EM physicians (85.7%)	Medical (78.4%)	84.3%	VL (54.3%)	Rate of RSI (74%) Etomidate (52%) Succinylcholine (43%)	16%	Hypoxia (11.9%)
BCARE	EM attending physicians (67.8%)	Intracranial hemorrhage/ stroke (14.6%)	85.2%	VL (57.5%)	Not reported	16.9%	Hypoxia (% not reported)
Chulalongkorn Airway Registry	EM 1st or 2nd year resident (53.6%)	Medical (35.5%), Congestive heart failure (18.6%)	74.3%	DL (84.5%)	Rate of RSI (57.4%) Etomidate (49.1%) Succinylcholine (55.5%)	13.2%	Hypotension (4.1%)
Cipto Mangunkusumo General Hospital Airway Registry	Anesthetists (63.8%)	Medical (93%), Respiratory failure (55.8%)	89.6%	Not reported	Rate of RSI (9%) Fentanyl (68.4%) Rocuronium (41.6%) No medication (21.6%)	22.1%	Hypotension (4.3%)
Cleveland Clinic Emergency Airway Registry	EM resident (91%)	Not reported	74%	DL (60.6%)	Rate of RSI (73%)**	Not reported	Not reported
Continuous Quality Improvement Database	EM physician PGY1 or 2 (56%)	Medical (66%), Airway protection (68%)	92.4%	VL (94%)	Rate of RSI (85.2%) Succinylcholine (60%) Etomidate (88%)	14.6%	Hypoxia (11.2%)
DREAM	EM PGY-2 (61%) EM PGY-1 (28%)	Trauma (64%)	93%	VL (86%)	Not reported	Not reported	Not reported
EDIR	EM physician (75.3%)	Medical (75%), cardiac arrest (21%)	85.5%	Not reported	Rate of RSI (68%) Thiopental (43%) Succinylcholine (65%)	9.8%	Hypotension (4.2%)
JEANI + II	EM attendings or residents (57%), transitional year resident (PGY1 or 2) (37%)	Medical (54%)	74%	DL (58%)	Rate of RSI (53%) Midazolam (49%) Rocuronium (84%)	14%	Esophageal intubation (4.4%)
KEAMR	PGY-1 EM resident (41%)	Medical (70.7%),"Anticipated oxygenation or airway protection failure" (40.3%)	84.8%	VL (55.4%)	Rate of RSI (61.3%)**	6.5%	Esophageal intubation (3.4%)
King Abdulaziz University Hospital Airway Registry	EM trainee (71.9%)	Airway protection (48.6%)	80%	VL (69.2%)	Not reported	Not reported	Esophageal intubation (3.4%)
NEARI	EM residents, EM attendings, non-EM residents (% not reported)	Not reported	Not reported	Not reported	Rate of RSI (79%)**	Not reported	Not reported
NEARII	EM physicians (87%)	Medical (67%), Cardiac arrest (12%)	81%	DL (86%)	Rate of RSI (69%) Etomidate 32.8%, Succinylcholine 75%	12%	Esophageal intubation (2.9%)
NEARIII	EM PGY2 (42%)	Altered mental status (26.4%)	91.9%	VL (80.3%)	Rate of RSI (83%) ​​Rocuronium (60%) Etomidate (78.9%)	16.5%	Hypoxia (10%)
NERRA	EM (54%) Anesthesia/ Intensive Care Medicine (46%) 90% at trainee level	Medical (83%), Cardiac arrest (30%)	89%	VL (53%)	Propofol (50%) Rocuronium (74%) No Sedation (23%) No paralytic (20%)	19%	Hypotension (10%)
Samsung Medical Centre Emergency Airway Program	EM PGY1 or 2 (50%)	Respiratory failure (50.1%)	79%	VL (60%)	Rate of RSI (84%) Etomidate (48%) Succinylcholine (62%)	8%	Esophageal intubation (6%)
Singapore General Hospital Emergency Airway Registry	EM physicians (98.6%) EM attendings (60%)	Medical (86.4% -91.5%)	77.5%	DL (87.3%)	Rate of RSI (55.4%)**	12.9%	Not reported
The Aberdeen Royal Infirmary Airway Registry	EM, anaesthesia, ITU doctors (% not reported)	Not reported	81.1%	Not reported	Not reported	13%	Hypoxia: With bougie (15.1%) No bougie (7.5%)
The Alfred Airway Registry	EM resident (63.5%)	Medical (56.4%)	93.8%	VL (82.5%)	Ketamine (42.3%) Rocuronium (52.1%)	20.4%	Hypoxia (9.6%)
The Royal North Shore Emergency Airway Registry	EM resident (57.5%)	Medical (70%)—Overdose/ingestion (% not reported)	93.9%	VL (92.7%)	Sedation used (92.4%) Paralytic used (92.6%)**	19.4%	Hypoxia (10.9%)
Middlemore Hospital Airway Registry	Not reported	Medical (52%), Intracranial hemorrhage/ stroke (12.4%)	Not reported	Not reported	Not reported	Not reported	Not reported
South African ED Registry	EM trainees (% not reported)	Medical (71.9%), Pulmonary causes (20.3%)	77.7%	VL (52%)	Etomidate (46.8%)	33%	Hypoxia (16.2%)

First pass success (*FPS*), Emergency Medicine (*EM*), Intensive Therapy Unit (*ITU*), Postgraduate year (*PGY*), video laryngoscope (*VL*), direct laryngoscope (*DL*), rapid sequence intubation (*RSI*)

^*^medical or trauma, with the most common indication within one of these two categories listed, if available

^**^no specific medications reported

Fourteen registries reported the most common indication for intubation as medical conditions including intracranial hemorrhage/stroke and airway protection. Only one registry reported trauma as the most common indication, Defense Registry for Emergency Airway Management (DREAM), a registry at Brooke Army Medical Centre, which is a level 1 trauma centre.

First pass success (FPS) rates were reported by 20 of 22 airway registries identified, with the exception of NEARI and the Middlemore Hospital Airway Registry. FPS ranged from 74% in the Japanese Emergency Airway Network I and II (JEANI + II) and the Cleveland Clinic Emergency Airway Registry to 93.9% in The Royal North Shore Emergency Airway Registry.

Seventeen of 22 registries reported the most used intubation device, either the video laryngoscope (VL) or direct laryngoscope (DL) in all of these registries. Twelve of these registries reported the VL as the most used with rates ranging from 52% in the South African ED Registry to 94% in the Continuous QI Database.

Medication use for intubation and rates of rapid sequence intubation (RSI) varied widely across the identified registries. Among the 13 registries that reported rates of RSI, rates ranged from 9% in the Cipto Mangunkusumo General Hospital Airway Registry to 85.2% in the Continuous QI Database. The most common induction agent was etomidate (7 of 22 registries). Ketamine, Propofol, Midazolam, Fentanyl, and Thiopental were each reported as the most common induction medication in one registry. Ten registries did not report their most common induction agent. Paralytic agent use was reported in 11 of 22 registries, with five of these reporting Rocuronium and six reporting Succinylcholine as the most used paralytic.

Total rates of adverse events were reported by 17 of 22 airway registries. These rates ranged from 6.5% in the Korean Emergency Airway Management Registry (KEAMR) to 33% in the South African ED Registry. Seventeen of 22 identified registries reported the most common adverse events associated with intubation. The most common adverse events were hypoxia in eight of 17 registries (7.5% to 16.2% of patients), hypotension in four of 17 registries (4.1% to 10% of patients), and esophageal intubation in five of these 17 registries (2.9 to 6% of patients).

### Definitions in identified airway registries

The identified airway registries provided various definitions of an intubation attempt, FPS, and adverse events, including hypoxia and hypotension. Table 5 provides a summary of these definitions in each registry, if available. Definitions provided in each included publication can be found in Additional File 5. Six of 22 registries defined an attempt at intubation as “a single passage of the laryngoscope blade into the mouth” while five registries made this definition more specific defining passage of the laryngoscope blade or endotracheal tube past various anatomical structures including past the lips (Royal North Shore), the teeth (JEANI + II and KEAMR), the vocal cords (Singapore General Hospital Emergency Airway Registry) or the alveolar ridge (NEARIII) as an intubation attempt. Eight registries did not define an intubation attempt.

**Table 5:** Definitions in identified airway registries

Name of Airway Registry	Definition of Intubation Attempt	Definition of FPS	Definition of hypoxia	Definition of hypotension
ANZEDAR	Single pass of the laryngoscope blade into the mouth	Successful placement of an endotracheal tube following the first pass of the laryngoscope into the mouth	Peripheral oxygen saturation < 93% measured by pulse oximeter	SBP < 90 mmHg
BCARE	Not reported	Not reported	Not reported	Not reported
Chulalongkorn Airway Registry	Attempt of laryngoscopy	Successful intubation during the first attempt	Not reported	Not reported
Cipto Mangunkusumo General Hospital Airway Registry	Not reported	Not reported	Oxygen saturation < 93%	Decrease in SBP requiring treatment with IV fluids
Cleveland Clinic Emergency Airway Registry	Single insertion of a laryngoscope for oral intubations or a single insertion of an endotracheal tube for nasal attempts	Not reported	Not reported	Not reported
Continuous Quality Improvement Database	Insertion of the laryngoscope blade into the mouth of the patient, regardless of whether an attempt was made to insert a tracheal tube	Successful tracheal intubation on a single laryngoscope insertion	Oxygen saturation < 93%	SBP < 90 mmHg
DREAM	Not reported	Not reported	Not reported	Not reported
EDIR	Not reported	Not reported	Oxygen saturation < 90%	SBP < 90 mmHg
JEANI + II	Single insertion of a laryngoscope (or other device) past the teeth	Endotracheal tube being placed past the vocal cords, with confirmation by quantitative or colorimetric end-tidal carbon dioxide monitoring, on first attempt	Pulse oximetry saturation < 90%	SBP < 90 mmHg
KEAMR	Single insertion of the laryngoscope past the teeth	Proper placement of the endotracheal tube through the vocal cords on first attempt	Pulse oximetry < 90%	Not reported
King Abdulaziz University Hospital Airway Registry	Insertion of the laryngoscope blade into the oropharynx regardless of whether an attempt was made to pass the endotracheal tube	Successful tracheal intubation on a single laryngoscope insertion	Not reported	Not reported
NEARI	Not reported	Not reported	Not reported	Not reported
NEARII	Single effort to place an airway	Endotracheal tube placed through the vocal cords on first attempt	Not reported	Not reported
NEARIII	Any insertion of a laryngoscope beyond the alveolar ridge, whether or not an endotracheal tube was inserted	Successful intubation with a single laryngoscope blade insertion	Oxygen saturation < 90% during or immediately after attempt or desaturation of > 10% of absolute	SBP < 100 mmHg
NERAA	Single passage of laryngoscope into the mouth	Successful passage of endotracheal tube through vocal cords on first attempt	Oxygen saturation < 93%	Hypotension requiring treatment with fluid or inotropes
Samsung Medical Centre Emergency Airway Program	Placement of laryngoscope blade into mouth regardless of successful tube insertion into trachea	Successful endotracheal intubation on first intubation attempt	Decrease in oxygen saturation below 80% at any time within 30 min of intubation	SBP < 90 mmHg or mean BP < 65 mmHg or requiring vasopressor administration or an increased dose of vasopressor or a decrease in BP by > 20%, using the lowest vital signs during 30 min after intubation
Singapore General Hospital Emergency Airway Registry	Attempt to pass the endotracheal tube through the vocal cords	Not reported	Not reported	Not reported
The Aberdeen Royal Infirmary Airway Registry	Not reported	Not reported	Not reported	Not reported
The Alfred Airway Registry	Single passage of the laryngoscope blade into mouth	Not reported	Oxygen saturation of < 93%	Hypotension requiring treatment with IV fluids/pressors
The Royal North Shore Emergency Airway Registry	Single passage of the laryngoscope blade past the lips	Correct placement of the endotracheal tube into the trachea, confirmed by end-tidal capnography, on the first attempt	Oxygen saturation < 93%	Hypotension requiring treatment with IV fluids
Middlemore Hospital Airway Registry	Not reported	Not reported	Oxygen saturation < 90%	Hypotension requiring treatment with IV fluids/pressors
South African ED Registry	Not reported	Successful intubation by the first operator on the first attempt	Oxygen saturation < 90%	SBP < 90 mmHg or 20% change from baseline

First pass success (*FPS*), Systolic blood pressure (*SBP*), Intravenous (*IV*)

FPS was most often defined as “successful placement of the endotracheal tube on the first laryngoscope insertion” in seven of 22 registries, while three defined FPS as “correct placement through the vocal cords on the first attempt” (National Emergency Resuscitation Airway Audit [NERAA], NEARII, KEAMR), and two stipulated that placement had to be confirmed by end-tidal capnography to be deemed successful (JEANI + II and Royal North Shore Emergency Airway Registry). Ten registries did not define FPS.

Thirteen of 22 registries defined specific parameters for hypoxia to be reported as an adverse event. Six registries reported hypoxia as peripheral oxygen saturation < 90%, six reported < 93%, and one reported < 80% within 30 min of intubation. NEARIII also specified that hypoxia would be reported as an adverse event if there was a desaturation of > 10% of absolute oxygen saturation.

Seven registries provided specific parameters to define hypotension as an adverse event. Hypotension was most often defined as a systolic blood pressure (SBP) of < 90 mmHg in six of these seven registries, whereas NEARIII defined hypotension as an SBP of < 100 mmHg. Among the registries that did not report a specific parameter, hypotension was defined as requiring treatment with either intravenous fluids or vasopressors in five registries. Both the South African ED Registry and the Samsung Medical Centre Emergency Airway Program also reported hypotension as an adverse event if a 20% change from the patients’ baseline SBP was recorded. The Samsung Medical Centre Emergency Airway Program included a third parameter to measure hypotension as a mean arterial pressure of < 65 mmHg. Ten registries did not define hypotension.

## Discussion

Of the identified registries, commonalities of reported information include patient demographics (most often age, sex, and weight), indication for intubation, method of intubation and adjuncts used (stylet, bougie), device used for each attempt, intubator level of training and specialty, number of attempts and the outcome of each attempt, presence of any difficult airway characteristics and predicted difficulty of intubation, induction and/or paralytic medications used, patients’ pre- and post-intubation vital signs, and complications or adverse events. Other commonly reported information included patient positioning, intubation maneuvers or rescue techniques, and the use of a pre-RSI checklist. Commonly reported adverse events included hypoxia, hypotension, bradycardia, esophageal intubation, failed airway requiring surgical airway, dysrhythmia or cardiac arrest, vomiting or aspiration, endobronchial intubation, dental or airway trauma, and laryngospasm.

There is a suggestion that the context of the registry is also important when comparing intubation performance. For example, the Cleveland Clinic Emergency Airway Registry also included pre-hospital intubations that comprised 30% of their data in one study [39]. Interestingly, this registry also reported the lowest FPS of North American airway registries, possibly due to the inclusion of pre-hospital intubations, which may be more difficult than ED intubations due to the inability to obtain assistance, lack of proper equipment, variability of intubator experience and training, and difficult intubation environments [43]. Additionally, 11 of 22 identified registries include intubations performed on patients of all ages, which complicates comparisons of intubation performance between institutions because of the added complexity of managing paediatric airways due to their smaller size, and anatomical and physiological differences from adult airways [44–46]. These inconsistencies of information reported, location of intubation, and patient population among airway registries may limit comparisons of intubation practices.

Airway registries are a critical QA tool that allow some degree of comparison of adverse events rates and key performance indicators of intubation practice between centres, both nationally and internationally. For example, Powell et al., 2018 utilized data from ANZEDAR as a benchmark to monitor intubation practices in a small, rural ED in New Zealand [47]. Using the data pooled from 43 centres in ANZEDAR, this study demonstrated that FPS rates and complication rates were similar in this rural ED to data collected from across Australia and New Zealand [47]. On a larger scale, Park et al., 2017 utilized real-time clinical data collected from airway registries internationally to determine an international benchmark for FPS of 84.1%, and provided rates of the most common peri-intubation adverse events [48].

Several studies identified in this review demonstrate that airway registry data play a crucial role in identifying deficiencies in ED intubation practices, developing targeted solutions, and monitoring the efficacy of multi-faceted QI programs to improve FPS while minimizing rates of peri-intubation adverse events [2, 11, 13, 39, 40, 49–55]. For example, Hwang et al., 2018 standardized intubation practices by introducing an ED intubation protocol checklist that directed staff to optimize pre-oxygenation, utilize VL, use RSI as a standard method for intubation, and limit intubation attempts to two by a single provider or with the same device [50]. This centre also provided evidence-based lectures on airway management to EM physicians and nurses, as well as hands-on skills training with various airway devices in a simulated setting [50]. After implementation of these changes, airway registry data collected in the subsequent three-year study period demonstrated that FPS increased by 11%, and overall peri-intubation complication rates decreased by 8% [50]. Tracking the efficacy of these QI interventions using airway registry data is crucial as it is well-established that multiple intubation attempts lead to higher rates of life-threatening peri-intubation complications, an increased risk of intubation failure on subsequent attempts, and lower probability of return of spontaneous circulation [3, 21, 22, 56–60].

The airway registries reviewed provide insight into historical practices and current trends. Airway registries identified in this review revealed a trend towards higher rates of intubations performed by RSI in America [13, 61–63], Europe [19, 64], and Australia and New Zealand [1, 47]. While rates of RSI are increasing in more recent publications, rates of RSI remain low in Japan [65–67] and Indonesia [24]. Likewise, DL has historically been the standard of care for performing ED intubations; however, trends in the identified airway registries demonstrate the increasing prevalence of VL as this intubation technique allows for higher rates of FPS with lower rates of adverse events [2, 8, 13, 27, 61, 68–73]. Trends in medication selection are also described in airway registry data, and this review demonstrated a trend towards increasing use of etomidate, despite the persistence of geographic variation in medication selection. Airway registries can be a valuable tool to increase awareness of how local practices compare to global trends in clinical care and their impact, such as standardizing the use of RSI for intubation, to provide better patient outcomes [4, 8, 52, 71, 76–78].

FPS is often used as a marker of intubation proficiency because multiple intubation attempts are associated with increased rates of adverse events [3, 21, 56–60]. Several studies included in our review reported the definition of an intubation attempt and FPS utilized at their institution; however, these definitions had some heterogeneity. The most recent systematic review performed to determine an international benchmark of FPS recommended defining FPS as “the proportion of endotracheal tubes placed successfully after the first attempt” and defining intubation attempt as “any single insertion of the laryngoscope into the mouth” [48]. This aligns with the most commonly reported definitions of FPS and intubation attempt identified in this review; however, there is still heterogeneity and a lack of transparent reporting of the definitions used by many airway registries identified which limits international comparison. To allow for the creation of reliable international benchmarks and equivalent comparisons of airway management performance between centres, we recommend that these definitions are used at all institutions implementing an airway registry.

Similarly, several identified airway registries did not provide definitions of common adverse events such as hypoxia and hypotension [12, 13, 15, 23, 34, 39, 49, 55]. Among those that provided definitions of these adverse events, there was a great deal of variability. A lack of agreed upon definitions of peri-intubation adverse events may explain the variability in reported overall adverse event rates among studies identified in this review, ranging from 6.5 to 33% [25, 79]. While there is little consensus of adverse event definitions that should be used in ED airway registries, guidelines do exist for these definitions in pre-hospital airway management [80]. Intubation attempts at a peripheral oxygen saturation of 93% or less have a much higher risk of progressing to critical oxygen desaturations [81]. This is in-keeping with the rationale that ANZEDAR provided for reporting hypoxia as an oxygen saturation of less than 93%, as they preferred this higher cut-off to provide a margin of safety before critical desaturation occurs [11]. Additionally, the most commonly used value of hypotension identified in this review was a SBP of less than 90 mmHg, in-keeping with the value recognized in some guidelines provided in pre-hospital airway management that report hypotension as a SBP less than 90 mmHg or a decrease in SBP of greater than 10% from the patient’s baseline value [82]. Based on the available literature, airway registries should consider reporting peri-intubation adverse events as hypoxia if peripheral oxygen saturation is 93% or less and hypotension if SBP falls below 90 mmHg or greater than 10% from the patient’s baseline measurement.

Of note, few publications provided meaningful information regarding the logistics of the airway registry use, sources of funding, methods of data collection or dissemination of information which would be valuable for consideration of how to optimally implement an ED airway registry.

### Limitations

This study reports only published data from airway registries or registry-like reporting procedures. We suspect that data from smaller or non-academic institutions, or those who did not provide evidence of an ongoing airway registry in their publication were likely not represented in this review which likely amounts to a degree of selection bias. Additionally, non-English publications were excluded from this review leading to an English-language bias.

Several of the identified registries include only a single publication or publications that are now dated. This may not be representative of the current state of intubation practices or the ongoing function of the airway registry within those institutions. The wide range of publication dates also means that data among various airway registries may no longer be representative of that airway registry. We have also included abstract only publications in this review to be as representative as possible of global airway registries; however, these publications do provide limited information when compared to full-text publications.

Lastly, we attempted to report the most recent and representative trends within each included airway registry. Data was reported from the most recent available publication. If the most recent publication was missing any of this data, it was reported from the next most recent publication, and so on. This method often led to intubation practices being reported from a single study within a registry and may not be representative of the nuances of emergency airway management among included institutions.

## Conclusions

This scoping review identified 22 airway registries globally that monitor ED intubation. Airway registries appear to be a crucial tool to improve intubation procedures and patient outcomes by contributing meaningful longitudinal data to guide local intubation practices, ensure quality of care, and serve as a platform to innovate and test clinically relevant questions, while providing important information regarding current intubation procedure trends and guidance for quality improvement initiatives. However, standardized definitions and transparent reporting of adverse events and other key performance indicators, such as FPS, would allow airway management performance to be compared on a more equivalent basis and allow for the determination of more reliable international benchmarks. With some consensus among pre-hospital airway registries and identified ED airway registries, it would be reasonable to adopt reporting hypoxia as a peripheral oxygen saturation of 93% or less and hypotension as SBP of less than 90 mmHg or a decrease of greater than 10% from the patient’s baseline. Additionally, FPS should be defined as “the proportion of endotracheal tubes placed successfully after the first attempt” and intubation attempt as “any single insertion of the laryngoscope into the mouth.” Given the importance of airway registry data, the creation and implementation of an airway registry should be considered by any centre looking to investigate and improve their emergency airway practices.

## Supplementary Information

**Additional file 1.** Search Strategy.

**Additional file 2.** Characteristics of Identified Airway Registries.

**Additional file 3.** QI/QA Studies.

**Additional file 4.** Research Studies.

**Additional file 5.** Intubation Practices and Adverse Events in Identified Airway Registries.

## Data Availability

All data generated or analyzed during this study are included in this published article and its supplementary information files.

## Abbreviations

ED: Emergency department
EM: Emergency medicine
QA: Quality assurance
QI: Quality improvement
PRISMA-ScR: Preferred reporting items for systematic reviews and meta-analysis extension for scoping reviews
ICU: Intensive care unit
BCARE: British Columbia airway registry for emergencies
ANZEDAR: Australian and New Zealand emergency department airway registry
NEAR: National emergency airway registry
EDIR: Emergency department intubation registry
DREAM: Defense registry for emergency airway management
FPS: First pass success
JEANI + II: Japanese emergency airway network I and II
VL: Video laryngoscope
DL: Direct laryngoscope
RSI: Rapid sequence intubation
KEAMR: Korean emergency airway management registry
NERAA: National emergency resuscitation airway audit
SBP: Systolic blood pressure
