# Supplementary material for: Airway registries in primarily adult, emergent endotracheal intubation: a scoping review
Source: Scand J Trauma Resusc Emerg Med. 2023 Mar 8;31:11. doi: 10.1186/s13049-023-01075-z (PMC9993388; doi:10.1186/s13049-023-01075-z)
Supplement: Supplementary file 1 — Additional file 1. Search Strategy. [file 13049_2023_1075_MOESM1_ESM.docx]

Additional File 1: Search Strategy for airway registries in primarily adult, emergent endotracheal intubation: a scoping review

**Database:**
Ovid MEDLINE(R) ALL <1946 to November 02, 2021>

| **#** | **Query** | **Results from 3 Nov 2021** |
| --- | --- | --- |
| 1 | (airway adj3 registr*).ab,kw,ti. | 148 |
| 2 | (airway or intubat* or rapid sequence induct*).ab,kw,ti. | 197,529 |
| 3 | airway management/ or intubation, intratracheal/ | 40,147 |
| 4 | first pass success.ab,kw,ti. | 330 |
| 5 | airway complicat*.ab,kw,ti. | 871 |
| 6 | 2 or 3 or 4 or 5 | 211,947 |
| 7 | Emergency Medicine/ | 14,402 |
| 8 | Emergency Service, Hospital/ | 78,556 |
| 9 | (emerg* adj3 (department* or medicine or service* or intubat* or ward*)).ab,kw,ti. | 139,826 |
| 10 | 7 or 8 or 9 | 173,960 |
| 11 | quality assurance, health care/ or benchmarking/ | 70,563 |
| 12 | (quality assurance or quality improvement or benchmark* or bench mark*).ab,kw,ti. | 118,815 |
| 13 | 11 or 12 | 169,238 |
| 14 | 6 and 10 and 13 | 194 |
| 15 | 1 or 14 | 333 |

**Database:**
Embase Classic+Embase <1947 to 2021 November 02>

| **#** | **Query** | **Results from 3 Nov 2021** |
| --- | --- | --- |
| 1 | (airway adj3 registr*).ab,kw,ti. | 294 |
| 2 | (airway or intubat* or endotrach* intubat*).ab,kw,ti. | 301,646 |
| 3 | exp respiration control/ | 19,323 |
| 4 | exp endotracheal intubation/ or exp respiratory tract intubation/ or exp rapid sequence induction/ | 64,468 |
| 5 | first pass success.ab,kw,ti. | 513 |
| 6 | airway complicat*.ab,kw,ti. | 1,382 |
| 7 | 2 or 3 or 4 or 5 or 6 | 338,464 |
| 8 | emergency medicine/ | 43,104 |
| 9 | (emerg* adj3 (department* or medicine or service* or intubat* or ward*)).ab,kw,ti. | 211,511 |
| 10 | exp emergency ward/ | 171,443 |
| 11 | 8 or 9 or 10 | 289,277 |
| 12 | exp quality control/ or exp benchmarking/ | 450,491 |
| 13 | (quality assurance or quality improvement or benchmark* or bench mark*).ab,kw,ti. | 167,113 |
| 14 | 12 or 13 | 534,182 |
| 15 | 7 and 11 and 14 | 505 |
| 16 | 1 or 15 | 778 |

**Database: Scopus – November 3, 2021**

Search Strategy:

( TITLE-ABS-KEY ( airway W/3 registr* ) ) OR ( TITLE-ABS-KEY ( emerg* W/3 ( med* OR department* OR ward* OR service* OR intubat* ) ) AND TITLE-ABS-KEY ( "bench mark*" OR benchmark* OR "quality improvement" OR "quality assurance" ) AND TITLE-ABS-KEY ( "airway complicat*" OR "first pass success" OR intubat* OR "airway management" ) )

537 results

**Cochrane Library**

Search Strategy:

Search Name: Airway Registry Scoping Review

Date Run: 04/11/2021 01:46:06

Comment:

ID Search Hits

#1 (airway NEAR/3 registr*):ti,ab,kw 31

#2 ((airway or intubat* or "rapid sequence" NEXT induct*)):ti,ab,kw 39923

#3 MeSH descriptor: [Airway Management] this term only 294

#4 MeSH descriptor: [Intubation, Intratracheal] this term only 3978

#5 MeSH descriptor: [Rapid Sequence Induction and Intubation] this term only 9

#6 ("first pass success"):ti,ab,kw 190

#7 (airway NEXT complicat*):ti,ab,kw 227

#8 {OR #2-#7} 40003

#9 MeSH descriptor: [Emergency Medicine] this term only 281

#10 MeSH descriptor: [Emergency Service, Hospital] this term only 2454

#11 ((emerg* NEAR/3 (department* or medicine or service* or intubat* or ward*))):ti,ab,kw 17192

#12 {OR #9-#11} 17192

#13 MeSH descriptor: [Quality Assurance, Health Care] this term only 612

#14 MeSH descriptor: [Benchmarking] this term only 115

#15 ("quality assurance" or "quality improvement" or benchmark* or bench NEXT mark*):ti,ab,kw 6019

#16 {OR #13-#15} 6019

#17 #8 AND #12 AND #16 10

#18 #1 OR #17 40

**Google Scholar (for component of grey literature)**

Search Strategy:

“airway registry” and “emergency medicine” = 1050 results

“airway registry” and “emergency department” = 1150 results

“airway registry” and “emergency” and “quality improvement” = 402 results

“airway registry” and “emergency” and “quality assurance” = 133 results

“airway registry” and “emergency” and “benchmark” = 95 results

“intubation registry” and “emergency medicine” = 95 results

“intubation registry” and “emergency department” = 113 results

“intubation registry” and “emergency” and “quality improvement” = 66 results

“intubation registry” and “emergency” and “quality assurance” = 32 results

“intubation registry” and “emergency” and “benchmark” = 28 results

The first 100 results were screened based on title and abstract, relevant searches were added to Covidence for review by a second team member

**Web of Science – November 3, 2021**

Search Strategy:

( TS= ( airway NEAR/3 registr* ) ) OR ( TS= ( emerg* NEAR/3 ( med* OR department* OR ward* OR service* OR intubat* ) ) AND TS= ( "bench mark*" OR benchmark* OR "quality improvement" OR "quality assurance" ) AND TS= ( "airway complicat*" OR "first pass success" OR intubat* OR "airway management" ) )

354 results
