# Supplementary material for: Airway registries in primarily adult, emergent endotracheal intubation: a scoping review
Source: Scand J Trauma Resusc Emerg Med. 2023 Mar 8;31:11. doi: 10.1186/s13049-023-01075-z (PMC9993388; doi:10.1186/s13049-023-01075-z)
Supplement: Supplementary file 2 — Additional file 2. Characteristics of Identified Airway Registries. [file 13049_2023_1075_MOESM2_ESM.docx]

Additional File 2: Characteristics of Airway Registries

| **Name of Registry** | **Author, Year of Publication** | **Abstract or Full-text** | **Geographic Location** | **Single or Multi-Centre (number of centres)** | **Total Number of Patients included** | **Time Period of Data Collection** | **Location of Intubations** | **Study Population** | **Aims of Study** |
| --- | --- | --- | --- | --- | --- | --- | --- | --- | --- |
| ANZEDAR | Freeman et al., 2021 | Full-text | Australia and New Zealand | Multi-centre (43) | 5,063 (total), 2,229 (included) | 2012-2019 | ED only | All patients 16+ intubated in the ED with an induction agent that is not etomidate | Describe and compare the hemodynamic effects of propofol, ketamine, and thiopentone during RSI. Identify predictors of hemodynamic instability |
|  | Arnold et al., 2021 | Full-text | Australia and New Zealand | Multi-centre | 4,806 | 2010-2015 | ED only | All patients intubated in the ED requiring a second attempt (7.1% paediatrics aged 18 or younger) | Describe current management practices after a failed intubation attempt in Autralian and New Zealand EDs; Explore factors associated with second attempt success |
|  | Perera et al., 2021 | Full-text | Australia and New Zealand | Multi-centre (43) | 3,710 (total), 2,831 (included) | 2013-2015 | ED only | All patients intubated in the ED not intubated due to cardiac arrest. those who received active ventilation prior to the first intubation attempt and where the use of apneic oxygenation were not recorded either way were excluded. (% paediatrics not reported, median age was 48) | Investigate whether the use of nasal oxygen during efforts securing a tube aponeic oxygenation during ED RSI decreases the incidence of desauration. Examine the contribution of aponeic oxygenation on the incidence of desaturation in the context of other potentially contributing factors. |
|  | Alkourhi et al., 2021 | Full-text | Australia and New Zealand | Multi-centre | 4,806 (total), 15 (included) | 2010-2015 | ED only | All patients undergoing emergency front-of-neck access in the ED (0% paediatrics) | Describe management of cases resulting in an emergency front-of-neck access |
|  | Ferguson et al., 2019 | Full-text | Australia and New Zealand | Multi-centre (43) | 4,388 | 2010-2015 | ED only | All patients aged 2+ undergoing intubation in the ED where an induction agent was used (3% of patients aged 2-16) | Describe the incidence of ketamine use in Australasian EDs and evaluate whether there has been any increase in its use. Identify predictors of ketamine use |
|  | Powell et al., 2018 | Full-text | New Zealand (Wanganui) | Single centre | 23 (total, included) | 2014-2015 | ED only | All patients who underwent endotracheal intubation in the ED (17% paediatric - specific age cut off not reported) | Describe the practice of intubation in a rural ED in New Zealand |
|  | Alkourhi et al., 2017 | Full-text | Australia and New Zealand | Multi-centre (43) | 3,710 (total, included) | 2013-2015 | ED only | All patients with attempted endotracheal intubation (4.8% paediatrics aged 0-15) | Describe the practice of endotracheal intubation across Australasian EDs |
|  | Fogg et al., 2015 | Abstract | Australia and New Zealand | Multi-centre (29) | >2000 | 2013-2015 (anticipated) | ED only |  | Create a pool of data for participating departments to use for self-improvement and for descriptive studies of the practice of intubation in Australasian EDs |
| BCARE | Botros et al., 2020 | Abstract | Canada | Multi-centre | 642 | 2017-2019 | ED only | All patients intubated by ED physicians and trainees | Compared intubation techniques, success, and complication rates between EM physicians and trainees |
|  | Yoo et al., 2018 | Abstract | Vancouver, Canada | Multi-centre (3) | 737 |  | 53.7% in ED, 30% in ICU, 16.3% on ward | All patients intubated outside OR - mean age 59.4, youngest age 17 | Describe development of BCARE network - an emergency intubation database at two tertiary care centres and one community hospital |
|  | Liu et al., 2018 | Abstract | Vancouver, Canada | Single Centre | 373 |  |  |  | Description of how a new airway registry was developed and implemented at a tertiary care centre in Canada |
| Chulalongkorn Airway Registry | Saoraya et al., 2021 | Full-text | Bangkok, Thailand | Single Centre | 226 (total), 220 (included) | 2017-2018 | ED only | Adult 18 or older) patients intubated in ED who were assessed for signs of difficult airway | To explore the incidence, management, and outcomes of patients with difficult airway predictors in an emergency department |
| Cipto Mangunkusumo General Hospital Airway Registry | Sulistio et al., 2021 | Full-text | Indonesia (Jakarta) | Single centre | 231 (total) | 2018-2019 | ED only | All patients intubated in the ED (8.7% paediatrics) | Determine first pass success rates and describe emergency intubation characteristics and outcomes at the centre |
| Cleveland Clinic Emergency Airway Registry | Good et al., 2017 | Abstract | USA (Cleveland) | Single centre |  |  | ED only | All patients intubated (age not reported) | Evaluate utilization trends of direct vs. video laryngoscopy over a four year period at the centre |
|  | Phelan et al., 2016 | Full-text | USA (Cleveland) | Single centre | 961 (included) | 2006-2007, 2008-2010 | ED (70%) and prehospital (30%) | All patients who underwent endotracheal tube intubation (no age reported) | Evaluate the use of a multi-faceted intervention designed to improve emergency physicians' documentation rates of the confirmation of ETT position is associated with lower mortality rates |
|  | Phelan et al., 2011 | Full-text | USA (Cleveland) | Single centre | 433 (total, included) | 2005-2007 | 65% in ED, 35% pre-hospital | All ED patients requiring invasive airway management (age not reported) | Determine the rate at which emergency physicians document confirmation of correct endotracheal tube location |
|  | Phelan et al., 2010 | Full-text | USA (Cleveland) | Single centre | 320 (total), 224 (included) | 2005-2007 | ED only | All patients intubated | Determine if an airway registry can be used as a tool to survey and evaluate EM airway management and if the collected data can eventually be used as a benchmark for comparison with a standard |
| Continuous Quality Improvement Database | Pacheco et al., 2021 | Full-text | USA (Tucson) | Single centre | 2,077 (total), 1,513 (included) | 2014-2018 | ED only | All patients 18+ intubated with RSI and either direct laryngoscopy or video laryngoscopy | Examine the association between physiologically and anatomically difficult airways using first pass success without adverse events |
|  | Sakles et al., 2019 | Full-text | USA (Tucson) | Single centre | 5,229 (total), 3,763 (included) | 2007-2017 | ED only | All patients 18+ that underwent RSI in the ED by an EM resident as the first operator | Describe the centre's airway continuous quality improvement program and its associated effect on the safety of airway management in the ED |
|  | Sakles et al., 2017 | Full-text | USA (Tucson) | Single centre | 4,626 (total), 1,985 (included) | 2007-2016 | ED only | All patients 18+ that underwent RSI in the ED by an EM resident using the resuable GlideScope or the direct laryngoscope | Determine the impact of a soiled airway on first pass success of emergency intubation, compare the success of the GlideScope and the direct laryngoscope in this context |
|  | JC Sakles, JM Mosier, AE Patanwala, B Arcaris, JM Dicken, 2016 | Full-text | USA (Tucson) | Single centre | 3,149 (total), 1,017 (included) | 2009-2015 | ED only | All adult patients (18+) intubated by RSI the C-MAC or Mac direct laryngoscope as the initial device | Evaluate the clinical utility of the C-MAC as a direct laryngoscope and determine its impact on first pass success compared to conventional Macintosh direct laryngoscope |
|  | JC Sakles, JM Mosier, AE Patanwala, JM Dicken, 2016 | Full-text | USA (Tucson) | Single centre | 856 (total), 127 (included) | 2013-2015 | ED only | All patients 18+ with an intracranial hemorrhage who underwent RSI in the ED by an EM resident with a starting oxygen saturation of >=90% | Determine the effect of apneic oxygenation on oxygen desaturation during the RSI of patients with intracranial hemorrhage in the ED |
|  | JC Sakles, JM Mosier, AE Patanwala, B Arcaris, JM Dickens, 2016 | Full-text | USA (Tucson) | Single centre | 1,140 (total), 635 (total) | 2013-2015 | ED only | All patients 18+ who underwent RSi in the ED by an EM resident with a starting oxygen saturation of >=90% | Determine the effect of apneic oxygenation on first pass success without hypoxemia |
|  | Dicken et al., 2016 | Abstract | USA (Tucson) | Single centre | 318 | 2014-2016 | ED only | All patients 18+ undergoing RSI by an EM resident using either the C-MAC or GVL-MAC | Compare FPS of the C-MAC and GlideScope-Mac when used for video laryngoscopy and direct laryngoscopy in the ED |
|  | JC Sakles, PP Javedani, E Chase, J Garst-Orozco, JM Guillen-Rodriguez, U Stolz, 2015 | Full-text | USA (Tucson) | Single centre | 2,677 (included) | 2007-2013 | ED only | All patients with intubation attempts in the ED by EM residents with a DL or VL (mean age 44.8 years) | Compare the incidence of esophageal intubations when EM residents use a direct laryngoscopy vs. a video laryngoscopy for intubation attempts in the ED |
|  | JC Sakles, JM Mosier, AE Patanwala, JM Dicken, L Kalin, PP Javedani, 2015 | Full-text | USA (Tucson) | Single centre | 2,587 (total), 398 (included) | 2009-2014 | ED only | All patients 18+ undergoing orotracheal intubation attempts by the same emergency physicians for all attempts, using CMAC or DL for the second attempt | Compare the CMAC with the direct laryngoscope to determine success when used for a rescue attempt after a failed initial intubation attempt |
|  | Arcaris et al., 2015 | Abstract | USA (Tucson) | Single centre | 1,873 | 2007-2015 | ED only | Adult patients only (18+) that underwent RSI by EM resident with a Mac VL or DL | Compare performance of EM residents using video-enabled Macintosh direct laryngoscope blade compared to conventional Macintosh direct laryngoscope blade |
|  | Corn et al., 2015 | Abstract | USA (Tucson) | Single centre | 345 | 2007-2015 | ED only | All adult patients 18+ with a bloody airway who underwent RSI in the ED with a standard GVL or DL | Compare effectiveness of GlideScope video laryngoscope to direct laryngoscope in patients with bloody airways |
|  | Patanwala et al., 2014 | Full-text | USA (Tucson) | Single centre | 2,258 (total), 2,098 (included) | 2007-2012 | ED only | All patients who underwent RSI in the ED, where ketamine or etomidate was used (9.24% paediatrics) | Compare first past intubation success between etomidate and ketamine use for rapid sequence intubation |
|  | JC Sakles, J Mosier, AE Patanwala, J Dicken, 2014 | Full-text | USA (Tucson) | Single centre | 1,613 (included) | 2007-2014 | ED only | Adult patients (18+) undergoing initial intubation attempt by EM residents using DL or GVL | Compare the learning curves for direct laryngoscopy and GlideScope video laryngoscopy |
|  | JC Sakles, AE Patanwala, J Mosier, J Dicken, N Holman, 2014 | Full-text | USA (Tucson) | Single centre | 2,972 (total), 583 (included) | 2007-2013 | ED only | All patients 18+ who underwent initial intubation attempts using the GVL or the cGVL using a stylet | Compare the first pass success and clinical performance characteristics of the GlideScope video laryngoscopy and Cobalt GlideScope video laryngoscope |
|  | JC Sakles, AE Patanwala, JM Mosier, JM Dicken, 2014 | Full-text | USA (Tucson) | Single centre | 2,834 (total), 1,600 (included | 2007-2013 | ED only | All patients 18+ with attempted intubation in the ED by an EM physician with difficult airways and using either VL or DL | Compare the efficacy of video laryngoscopy to direct laryngoscopy on first pass intubation success of patients with difficult airway characteristics |
|  | Mosier et al., 2013 | Full-text | USA (Tucson) | Single centre | 1,475 (total), 463 (included) | 2009-2012 | ED only | All patients intubated with GlideScope video laryngoscope or the C-MAC as the initial device. Mean age: 47.7 - 49.6 | Compare intubation success between GlideScope video laryngoscope and C-MAC laryngoscopy in ED patients |
|  | JC Sakles, CS Chiu, J Mosier, C Walker, U Stolz, 2013 | Full-text | USA (Tucson) | Single centre | 1,850 (total), 1,828 (included) | 2007-2011 | ED only | All patients undergoing orotracheal intubation in the ED | Describe the association between multiple intubation attempts and incidence of adverse events during ED intubations |
|  | JC Sakles, J Mosier, M Cosentino, A Patanwala, 2013 | Abstract | USA (Tucson) | Single centre | 350 (included) | 2008-2012 | ED only | All patients who sustained blunt or penetrating trauma to the head who underwent RSI in the ED (age not specific) | Determine the incidence of hypoxemia during rapid sequence intubation of head-injured patients in the ED |
|  | J Sakles, D Falvey, N Stea, U Stolz, 2013 | Abstract | USA (Tucson) | Single centre | 2457 |  | ED only | All patients intubated in the ED over a 5-year period (age not reported) | Describe if operator-identified patient obesity was associated with reduced first-pass success rate |
|  | JC Sakles, J Moiser, S Chiu, M Cosentino, L Kalin, 2012 | Full-text | USA (Tucson) | Single centre | 1,117 (total), 750 (included) | 2009-2011 | ED only | Patients intubated in ED | Compare performance of C-MAC video laryngoscope to the Macintosh direct laryngoscope in intubated ED patients |
|  | Sakles and Kalin, 2012 | Full-text | USA (Tucson) | Single centre | 1,846 (total), 473 (included) | 2007-2011 | ED only | All patients intubated in the ED using the GlideScope Standard, Cobalt, or Ranger as the initial device (age not specified, mean age 37.6-43.3 years) | Determine whether using the GlideRite rigid stylet compared with a standard malleable stylet affects success rate of intubation using the GlideScope in ED intubations |
|  | Sakles et al., 2012 | Full-text | USA (Tucson) | Single centre | 943 (total), 583 (included) | 2007-2009 | ED only | All patients requiring intubation in the ED using a GlideScope video laryngoscope or traditional laryngoscopy (age not reported) | Compare the performance of GlideScope video laryngoscopy and direct laryngoscopy in the ED |
|  | Patanwala et al., 2011 | Full-text | USA (Tucson) | Single centre | 621 (total), 327 (included) | 2007-2008 | ED only | All 18+ patients intubated in the ED with RSI, with etomidate for induction, and succinylcholine or rocuronium for paralysis | Determine the effect of paralytic type and dose on first-attempt rapid sequence induction success in the ED, and other factors that may be associated with FPS |
| DREAM | Mendez et al., 2021 | Full-text | USA (San Antonio) | Single centre | 74 (total, included) | 2020 | ED only | All patients 18+ intubated – Centre serves active duty personnel, retired military beneficiaries, and dependents | Describe pilot data collected as part of the development of the Defense Registry for Emergency Airway Management (DREAM) |
| EDIR | Hale et al., 2017 | Full-text | Edinburgh (UK) | Multi-centre (7) | 3988 (total), 3601 (included) | 1999-2011 | ED only | All ages - this adult ED mostly manages patients 13 or older. In this study <13 0.2%, 13-16 = 1.8%, 17-24 = 9.6%, 25+ = 88.3% | Describing intubation practices, technique, drugs, rates of success, and adverse events in adolescents (13-16 years), 17-24 years (young adult), and at least 25 years (older adult) |
|  | Kerslake et al., 2015 | Full-text | Edinburgh (UK) | Multi-centre registry (7)- this study was single centre | 3988 (total), 3738 (included) | 1999-2011 | ED only | All ages (only 0.2% aged less than 13) | Describe intubation practices at this single centre within the EDIR |
|  | Paul et al., 2012 | Full-text | Edinburgh (UK) | Single centre | 2234 (total), 80 (included) | 1999-2006 | ED only | Adult patients undergoing intubation without medication administration in the ED (all those undergoing RSI were excluded) - age not specified, median age 69 | To determine long-term survival rates in critically ill medical patients undergoing intubation without medication assistance. For this study medical patients = any patient who was not in cardiac arrest, had no trauma, and no surgical diagnosis |
|  | Reid et al., 2011 | Full-text | Edinburgh (UK) | Multi-centre (7) | 2524 (total) | 1999-2007 | ED only | All patients aged 13+ undergoing ED intubation (% paediatrics not reported) | To determine the frequency of and primary indication for surgical airway during emergency department intubation, and the primary indication for surgical airway |
|  | Donald, 2011 | Full-text | Dundee (UK) | Single-centre study (registry is multi-centre) | 329 | 2005-2009 | 86% ED, 14% pre-hospital | Patients undergoing RSI - 3% were children (14 or younger), and 14% were performed in pre-hospital environment, rest were adult ED patients | To illustrate RSI training and experience attained as a specialist registrar in a Scottish teaching hospital emergency department and establish whether it is sufficient to progress to independent practice. |
|  | Stevenson et al., 2007 | Full-text | Scotland, UK | Single centre | 234 (total), 199 (included) | 2003-2006 | ED only | Patients of all ages undergoing intubation in ED (9% paediatrics) | To characterize intubation practice in a Scottish ED over 40 months. |
|  | Graham et al., 2003 | Full-text | Edinburgh (UK) | Multi-centre (7) | 1631 (total), 735 (included) | 1999-2001 | ED only | Adult Patients undergoing RSI within the ED (median age 44-49, % paediatrics not reported) | To determine current practice for rapid sequence intubation (RSI) in a sample of emergency departments in Scotland performed by EM physicians or anesthesiologists |
| JEANI+II | Yamanaka et al., 2020 | Full-text | Japan | Single centre study | 266 (total), 181 (included) | 2010-2016 | ED only | Adult patients undergoing ED intubation (no age cut off stated - median age 68) | Compare one or more intubation attempts in the ED With risk of morbidity and mortality during hospitalization - do multiple intubation attempts impact survival during hospitalization? |
|  | Goto et al., 2017 | Full-text | Japan | Multi-centre (14 - JEAN II) | 10,927 (total), 10,875 (included) | 2010-2012 (JEAN 1), 2012-2016 (JEAN 2) | ED only | All adult and paediatric patients intubated in the ED (3% aged 0-17) | Investigate the changes in practice and related outcomes in Japanese EDs |
|  | Okubo et al., 2017 | Full-text | Japan | Multi-centre (13) | 4,094 (total), 2,365 (included) | 2010-2012 | ED only | All adults and children intubated in the ED with medications and using a consistent method across attempts (3.7% paediatrics aged 18 years or younger) | Investigate intubation success and complication rates with the use of RSI compared to non-RSI methods in the ED |
|  | Nakao et al., 2015 | Full-text | Japan | Multi-centre (13) | 4,094 (total), 723 (included) | 2010-2012 | ED only | All patients intubated in the ED due to trauma (% paediatrics not reported, median age 56) | Describe the current practice of airway management for trauma patients in EDs in Japan |
|  | Goto et al., 2015 | Full-text | Japan | Multi-centre (13) | 4,094 (total), 1,289 (included) | 2010-2012 | ED only | All adult and paediatric patients with repeated tracheal intubation attempt in the ED and were ultimately successful (3% paediatrics aged <18 years) | Determine whether success rate at repeated attempts at tracheal intubation by a single intubator was lower than that by alternate intubators in the ED |
|  | Goto et al., 2014 | Full-text | Japan | Multi-centre (13) | 4,094 (total), 2,800 (included) | 2010-2012 | ED only | All adult and paediatric patients intubated in the ED by EM residents (% paediatrics not reported) | Examine success rate of ED airway management by residents in Japan EDs |
|  | Imamura et al., 2013 | Full-text | Japan | Multi-centre (11) | 3,277 (total), 3,178 (included) | 2010-2011 | ED only | All patients 18+ intubated in the ED | Characterize airway management for ED geriatric patients in Japan |
| KEAMR | Kim et al., 2017 | Full-text | Korea | Multi-centre (20) | 10,978 (total), 5,783 (included) | 2006-2010 |  | All non-cardiac arrest patients intubated in the ED by emergency physicians % paediatrics not reported, average age 58.02-59.82 years) | Evaluate the effectiveness of using a simulation-based emergency airway management education program (SBEAMP) through comparison of performance to other hospitals that don't use the program |
|  | Lee et al., 2016 | Full-text | Seoul, Korea | Multi-centre (2) | 3,386 (total), 2,817 (included) | 2006-2010, 2013-2015 | ED only | All patients 18+ who underwent oral endotracheal intubation in the ED by an EM physician without the use of surgical methods or extraglottic devices on first attempt | Assess changes of usage frequency and intubation success rates during a first attempt using GlideScope videolaryngoscopy and direct laryngoscopy |
|  | Choi et al., 2015 | Full-text | Korea | Multi-centre (13) | 4041 | 2007-2010 | ED only | Patients 18+ undergoing orotracheal intubation in the ED, cardiac arrest cases excluded. | Compare the use of GlideScope video laryngoscopy with Macintosh laryngoscopy for improving first attempt intubation success |
|  | Cho et al., 2015 | Full-text | Korea | Multi-centre (13) | 2,086 (total), 1,831 (included) | 2006-2010 | ED only | All trauma patients who received intubation attempts in the ED (% paediatrics not reported - mean age 45.9) | Evaluate factors affecting FPS in trauma patients, including intubation methods and devices used |
|  | Cho et al., 2013 | Full-text | Korea | Multi-centre (13) | 10,942 (total), 4,891 (included) | 2006-2012 | ED only | All patients 65+ requiring intubation in the ED | Evaluate current status and complications of emergency airway management in Korean elderly patients |
|  | Kim et al., 2013 | Full-text | Korea | Multi-centre (13) | 6,157 (total), 5,905 (included) | 2007-2010 | ED only | All patients 18+ who received advanced airway management with failed first attempt | Identify factors associated with successful second and third attempts after failed first intubation attempts in the ED |
|  | Kim et al., 2011 | Abstract | Korea | Multi-centre (6) | 1,867 (included) | 2006-2010 | ED only | Adult patients with out-of-hospital cardiac arrests intubated in the ED | Determine whether first attempt success rate of intubation using GlideScope video laryngoscope is different to Macintosh laryngoscope during CPR |
| King Abdulaziz University Hospital Airway Registry | Bakhsh et al., 2021 | Full-text | Saudi Arabia (Jeddah) | Single centre | 146 (total, included) | 2018-2020 | ED only | All ED patients requiring intubation (median age 55.56 years - % of peds not reported) | Evaluate the implementation of a local airway database and quality improvement program in the Kingdom of Saudi Arabia on first-pass success rate improvement |
| Middlemore Hospital Airway Registry | Brainard et al., 2014 | Poster | New Zealand (Auckland) | Single centre | 258 | 2014 | ED only | Age not reported | Describe the implementation of an airway registry at a single large ED in Auckland |
| NEARI | Sagarin et al., 2003 | Full-text | USA | Multi-centre (11) | 1,288 (total), 888 (included) | 1996-1997 | ED only | Patients intubated orally using a RSI technique (13.9% paediatrics 18 years or younger) | Determine the patterns of use of midazolam in the ED and assess compliance with recommended dosages. Determine whether any observed underdosing is a result of reduction of the midazolam dose when it is being used in combination with other agents. Compare the use of midazolam with that of other induction agents |
| NEARII | Walls et al., 2011 | Full-text | International | Multi-centre (31) | 8,937 | 1997-2002 | ED only | All ED patients with attempted intubation (% paediatrics not reported) | Describe emergency intubation indications, methods used, operator characteristics, and adverse event rates |
|  | Sagarin et al., 2005 | Full-text | USA, Canada, Singapore | Multi-centre (31) | 8,495 (total), 7,498 (included) | 1996-2001 | ED only | All patients intubated in the ED (% paediatrics not reported) | Examine the success of airway management by emergency medicine residents across North America |
|  | Delorio, 2005 | Full-text | International | Multi-centre (35) | 6695 (included) |  | ED only | All non-cardiac arrest ED patients requiring intubation (age not specified) | Evaluate the use of end-tidal carbon dioxide monitoring in non-cardiac arrest patients within emergency departments |
|  | Collins et al., 2005 | Abstract | International | Multi-centre (30) | 7,453 (total), 256 (included) | 1998-2001 | ED only | All patients requiring intubation secondary to asthma (% paediatrics not reported) | Evaluate characteristics of managing airways for patients with severe asthma in the ED setting |
|  | Bair et al., 2002 | Full-text | International (USA, Canada, Singapore) | Multi-centre (30) | 7,712 (total), 207 (included) | 1998-2001 | ED only | Patients intubated in the ED if the primary method of intubation was unsuccessful (3% paediatrics aged 0-17) | Describe prevalence of ED airway management failures requiring rescue maneuvers, describe successful rescue methods used when the primary method chosen is unsuccessful, and characterize the roles of emergency physicians and other specialists in rescue airway management |
|  | Walls et al., 1999 | Abstract | USA | Multi-centre (16) | 2,392 (total, included) | 1997-1998 | ED only | All ED intubation patients, adults and children (7.2% paediatrics younger than 18 years) | Characterize success and complication rates of US EM airway management |
| NEARIII | Nikolla et al., 2022 | Full-text | USA | Multi-centre (25) | 19,071 (total) | 2016-2018 | ED only | Adults medical patients >17 years of age undergoing ED VL intubation in non-supine position (ramped or upright) | To compare first attempt success between hyperangulated and standard geometry VL during ED intubations in non-supine positions |
|  | Trent et al., 2021 | Full-text | USA | Multi-centre (23) | 19,071 (total), 4,449 (included) | 2016-2018 | ED only | All patients intubated for trauma in the ED (4% aged 0-14) | Characterize endotracheal intubations in trauma patients, estimate first pass success and the associations between patient intubation characteristics and first pass success |
|  | Nikolla et al., 2021 | Full-text | International | Multi-centre (25) | 19,071 (total), 3,858 (included) | 2016-2018 | ED only | All patients 18+ who underwent oral intubation for non-trauma indications and not in cardiac arrest | Compare post-induction hypoxemia and other adverse events between the ramped and supine positions in ED intubations with apneic oxygenation |
|  | Driver et al., 2021 | Full-text | International | Multi-centre | 19,071 (total), 12,511 (included) | 2016-2018 | ED only | All patients 14+ intubated orally or nasally using RSI, ketamine alone as a sedating agent without a NMBA, or topical anesthesia facilitation | Evaluate FPS and adverse events for patients who underwent intubation using only ketamine, topical anesthesia, and RSI approaches |
|  | Sandefur et al., 2021 | Full-text | USA | Multi-centre (25) | 19,071 (total), 98 (included) | 2016-2018 | ED only | All patients undergoing airway management for angioedema in the ED (youngest patient was 17 years old, median age 59 years) | Describe the patient characteristics, emergency airway management techniques and outcomes among patients in the ED and managed for angioedema |
|  | Kaisler et al., 2021 | Full-text | International | Multi-centre (25) | 19,071 (total), 82 (included) | 2016-2018 | ED only | All patients with an awake intubation attempt (defined as use of topical airway anaesthesia only or plus sedation) as first attempt | Describe awake intubation practices in the ED |
|  | Levin et al., 2021 | Full-text | International | Multi-centre (25) | 19,071 (total), 8,034 (included) | 2016-2018 | ED only | All patients >14 who underwent RSI with rocuronium (between 0.5-2mg/kg dose) | Assess the relationship of escalating weight-based dosing for rocuronium on first attempt intubation success and peri-intubation adverse events |
|  | Chan et al., 2021 | Full-text | Singapore | Single centre | 669 | 2016-2018 | ED only | All patients 21+ requiring intubation in the ED | Describe intubation characteristics of ED intubations at the National University Hospital in Singapore |
|  | April et al., 2021 | Full-text | International | Multi-centre | 19,071 (total), 15,776 (included) | 2016-2018 | ED only | All patients 15+ intubated in the ED | Examine prevalence of peri-intubation cardiac arrest and its association with case features in the ED setting |
|  | Kunzler et al., 2021 | Abstract | International | Multi-centre (22) | 17,984 (included) | 2016-2018 | ED only | All patients 14 or older undergoing ED intubation for overdose vs. other indications | Quantify the frequency of peri-intubation adverse events for patients intubated in the ED for overdose, and determine whether first attempt success without adverse events differ from patients intubation for other reasons |
|  | Garcia et al., 2021 | Abstract | International | Multi-centre | 15,204 (included) | 2016-2018 |  | All patients who underwent an orotracheal intubation first attempt by an EM trainee using a device | Compare the learning curve of EM trainees of direct laryngoscope, hyperangulated blade video laryngoscope, and standard geometry blade video laryngoscopes |
|  | Godwin et al., 2020 | Full-text | International | Multi-centre (25) | 19,071 (total), 173 (included | 2016-2018 | ED only | All patients intubated with a primary indication of asthma (mean age 43.3 years, % paediatrics not reported) | Describe current airway management techniques, devices, and outcomes for patients undergoing intubation for severe asthma |
|  | Driver et al., 2020 | Full-text | International | Multi-centre | 19,071 (total), 11,927 (included) | 2016-2018 | ED only | All patients 14+ or older who were orally intubated with a standard-geometry or hyperangulated video laryngoscope on the first attempt | Compare outcomes of patients intubated with a standard-geometry video laryngoscope with those using a hyperangulated video laryngoscope |
|  | Mohr et al., 2020 | Full-text | International | Multi-centre (25) | 12,722 (total), 531 (included) | 2016-2017 | ED only | All patients intubated in the ED for sepsis (% paediatrics not reported) | Describe the induction agents used for sepsis patients requiring intubation in the ED; compare peri-intubation adverse events between etomidate and ketamine |
|  | April et al., 2020 | Full-text | International | Multi-centre (25) | 19,071 (total), 6,906 (included) | 2016-2018 | ED only | All patients >14 intubated in ED who were normotensive, undergoing RSI, and received either ketamine or etomidate | Compare patient outcomes between ketamine and etomidate use during ED intubations |
|  | Lembersky et al., 2020 | Full-text | International | Multi-centre (25) | 12,722 (total), 11,748 (included) | 2016-2017 | ED only | All patients intubated in the ED with sedatives or analgesic medications (% paediatrics not reported. Mean age in sedation was 50.8, mean age in no sedation was 54.3) | Examine rates of post-intubation sedation and identify associated factors |
|  | Brown et al., 2020 | Full-text | International | Multi-centre (25) | 11,714 (Total), 6,938 (included) | 2016-2017 | ED only | Patients >14 intubated in the ED using unassisted VL or augmented DL | Compare unaided video larngyoscopy (without bougie , ramped patient positioning, or external laryngeal manipulation) with combinations of augmented direct laryngoscopy |
|  | Runde et al., 2020 | Abstract | International | Multi-centre | 19,071 (total), 18,915 (included) | 2016-2018 | ED only | All patients intubated in the ED (% paediatrics not reported) | Determine if there is any association between the frequency of videolaryngoscopy use and proportion of first intubation attempts given to junior trainees |
|  | Watase et al., 2020 | Abstract | International | Multi-centre (25) | 19,071 (total), 10,141 (included) | 2016-2018 | ED only | All adult encounters aged 15+ patients intubated in the ED with either direct laryngoscopy or hyperangulated video laryngoscopy on first attempt | Analyze the association between first attempt success and glottic view with hyperangulated videolaryngoscopy compared to direct laryngoscopy |
|  | Joshi et al., 2020 | Abstract | USA (Boston) | Single centre | 237 | 2016-2017 | ED only | Patients requiring intubation in the ED, 18+ years old | To determine the impact of deep sedation immediately following ED intubation on mortality |
|  | Stoecklein et al., 2019 | Full-text | International | Multi-centre (25) | 12,722 (total), 11,480 (included) | 2016-2017 | ED only | All patients 18+ with recorded peri-intubation positioning undergoing RSI in the ED | Compare the rate of first pass success, peri-intubation adverse events, and Cormack and Lehane laryngeal view for patients undergoing intubations in supine and nonsupine positions |
|  | Monette et al., 2019 | Full-text | USA, Canada | Multi-centre (18) | 15,280 (total), 14,313 (included) | 2002-2012 | ED only | All patients intubated at sites with an EM training program performed by an EM provider using a direct laryngoscope, the C-MAC, or the GlideScope (4.9% paediatric) | Evaluate the impact of video laryngoscopy on the clinical learning environment of EM residents |
|  | Roy et al., 2019 | Abstract | USA | Multi-centre (25) | Over 12,000 (total, included) | 2016-2018 | ED only | Patients intubated in the ED in the USA (% paediatrics not reported) | Evaluate intubation methods and associations with adverse events and first pass success |
|  | Brown et al., 2019 | Abstract | USA | Multi-centre (25) | 19,071 (total), 18,915 (included) | 2016-2018 | ED only | All patients undergoing intubation in the Emergency Department (3.3% were paediatrics aged <15) | Determine if crcicothyrotomoy rates have declined, describe current incidence of cricothyrotomy techniques used, describe cricothyrotomy success and failure rates, and risk factors |
|  | Runde et al., 2019 | Abstract | USA | Multi-centre | 19,071 (total), 18,915 (included) | 2016-2018 | Not stated | All intubated ED patients, adults and children (% paediatrics not reported) | Compare peri-intubation adverse event rates between videolaryngoscopy and direct laryngoscopy in ED intubation, stratified by intubator level of training |
|  | April et al., 2018 | Full-text | International | Multi-centre (22) | 5,071 (total), 2,275 (included) | 2016 | ED only | All patients 15+ intubated in ED with succinylcholine or rocuronium (mean age 49.8 for succ, 54.3 for roc) | Compare first pass intubation success between succinylcholine and rocuronium use for ED rapid sequence intubation |
|  | Maddry et al., 2018 | Full-text | USA (San Antonio) | Single centre | 503 (total, included) | 2016-2018 | ED only | All patients intubated at the San Antonio Military Medical Centre ED (average age 45.8, % paediatrics not reported) | Evaluate various elements of endotracheal intubation |
|  | Hayden et al., 2018 | Full-text | International | Multi-centre (13) | 17,190 (total), 204 (included) | 2002-2012 | ED only | All patients 15+ who underwent a FFI in the ED | Characterize the use of flexible fibreoptic intubation in EDs |
|  | Goldberg et al., 2018 | Abstract | USA | Multi-centre (25) | 12,722 | 2016-2017 | ED only | All intubations performed in ED aged 15+ | Assess impact of bougie use on first pass success and ultimate success for difficult airways, evaluate the differential improvement in intubation success based on blade shape |
|  | Kilgo et al., 2018 | Abstract | USA (El Paso, Texas) | Multi-centre | 380 | 2016-2017 | ED only | All intubations in the ED over a one year period | Describe intubation practices at this single centre comparing to data from NEAR as a benchmark |
|  | Ruderman et al., 2018 | Abstract | International | Multi-centre | 12,722 (included) | 2016-2017 | ED only | All patients with attempted intubation in the ED with either direct laryngoscopy or video laryngoscopy with difficult airway characteristics (% paediatrics not specified) | Compare first pass success rates between video laryngoscopy and direct laryngoscopy in patients with predicted difficult airways |
|  | Van Oeveren et al., 2017 | Full-text | USA | Multi-centre | 206 (total, included) | 2014-2015 | Not specified | All ED patient intubation attempts with telemedicine activated -- participating centres mostly served towns of <5,000 people with large rural catchment areas, most are critical access hospitals with limited resources (7.8% paediatrics aged 18 or younger) | Describe telemedicine-assisted intubation in rural EDs served by large ED networks |
|  | April et al., 2017 | Full-text | USA (Houston) | Single centre | 259 | 2016-2017 | ED only | All patients intubated from March 2016 to March 2017 at Brooke Army Medical Centre- this centre serves active duty and retired military personnel, military beneficiaries, and nonmilitary trauma patients (% paediatrics not reported) | Describe ED endotracheal indication, intubation techniques, success and failure rates, adverse events, device, induction agents at Brooke Army Medical Centre |
|  | Carlson et al., 2015 | Full-text | USA | Multi-centre (13) | 17,583 (total), 325 (included) | 2002-2012 | ED only | All ED patients 15+ with an intubation attempt and indication of GI bleed | Compare intubation outcomes between patients with GI bleeds managed with video laryngoscopy vs direct laryngoscopy |
|  | Brown et al., 2015 | Full-text | International (USA, Canada, Australia) | Multi-centre (18, 13 included in study based on compliance with reporting) | 17,583 | 2002-2012 | ED only | All patients 15+ intubated in the ED | Analyze performance attributes and identify evolving trends in ED intubation practices |
|  | Brown et al., 2007 | Abstract | USA | Multi-centre | 81 | 2006-2007 | ED only | Convenience ED patients for whom the video laryngoscope was chosen as the intubating device (% paediatrics not reported, average age 55) | Evaluate DCI II video laryngoscope in the ED setting, especially compared to direct laryngoscopy |
| NERAA | Umana et al., 2022 | Full-text | Ireland | Multi-centre (11) | 118 | 2020  (3 months) | ED only | All patients over the age of 16 requiring emergency airway management | To describe emergency airway management of critically unwell patients presenting to Irish EDs |
| Samsung Medical Centre Emergency Airway Program | Kim et al., 2019 | Full-text | Korea (Seoul) | Single centre | 1,087 (total), 689 (included) | 2014-2017 | ED only | All patients 19+ intubated in the ED | Investigate whether the dose of sedative used during intubation could affect post-intubation hypotension and identify clinical factors that affect hypotension |
|  | Hwang, Park, et al., 2018 | Full-text | Korea (Seoul) | Single centre | 1,087 (total, included) | 2014-2017 | ED only | All patients 18+ intubated in the ED | Evaluate the effect of changes made as part of a quality improvement project in the ED |
|  | Hwang, Lee, et al., 2018 | Full-text | Korea (Seoul) | Single centre | 939 (total), 744 (included) | 2014-2016 | ED only | All patients 18+ intubated by EM residents with conventional direct laryngoscopy or C-MAC | Evaluate the usefulness of C-MAC as a training tool for direct laryngoscopy in the ED |
|  | Hwang and Jo, 2010 | Full-text | Seoul, Korea | Single Centre | 241 (total), 126 (included) | 2007-2008 | ED only | All non-trauma patients >15 years old undergoing ED intubation | To evaluate factors related to mortality of non-traumatic adult patients who had received intubation in an ED |
| Singapore General Hospital Emergency Airway Registry | Weng et al., 2021 | Full-text | Singapore | Single centre | 2,950 (total, included)) | 2009-2016 | ED only | All patients intubated in the ED – age not reported | Determine whether video laryngoscopy or direct laryngoscopy use affects first pass success rates for endotracheal intubations |
|  | Zakaria & Wong, 2017 | Abstract | Singapore | Single centre | 2,934 (total), 2,899 (included) | 2009-2016 | Not specified | Not reported | Compare first pass success rates among attending and non-attending emergency physicians using direct laryngoscopy and video laryngoscopy |
|  | Wong & Ngo, 2009 | Abstract | Singapore | Single centre | 172 (included) | 2008-2009 | Not specified | Age not reported | Document the airway characteristics of patients with difficult airways |
|  | Wong & Ng, 2008 | Full-text | Singapore | Single centre | 2,343 (total, included) | 2000-2006 | Not specified | Age not reported | Identify the reasons for difficult airways in ED, study the rescue methods used |
|  | Wong & Ho, 2006 | Full-text | Singapore | Single centre | 533 (162 pre-SARS, 162 during SARS, 209 post-SARS) | 2002-2004 | ED only | Age not reported | Study the effects of wearing powered air-purifying respirator (PAPR), PPE, and the restriction in the number of resuscitation personnel on airway management duing the SARS crisis |
| South African ED Registry | Hart & Goldstein, 2020 | Full-text | South Africa (Johannesburg) | Single centre | 374 (total), 371 (included) | 2015-2016 | ED only | All patients 18+ intubated in the ED | Analyze airway management characteristics in a South African centre and compare to international data |
| The Aberdeen Royal Infirmary Airway Registry | Yeap et al., 2019 | Abstract | UK (Aberdeen) | Single centre | 197 (included) | 2015-2017 | ED only | ED intubation patients given rapid sequence induction | Determine whether the addition of airway adjunct improves rate of first pass success |
| The Alfred Airway Registry | Groombridge et al., 2021 | Full-text | Australia (Melbourne) | Single centre | 783 (included): 647 pre, 136 post | 2017-2020 | ED only | All patients intubated in the ED (age not reported, mean age 43.2 - 45.4 years) | Identify the consequences of implemented modifications to intubation practice and impact of COVID-19 |
|  | Groombridge et al., 2020 | Full-text | Australia (Melbourne) | Single centre | 526 (included): 157 pre, 369 post | 2017-2019 | ED only | All patients intubated in the ED (age not reported, median age 43 years) | Examine whether a bundle of activities (a dedicated clinician-completed airway registry, monthly audit with targeted education based on issues identified from the registry, cognitive aid form of a checklist) would improve first attempt success rate and decrease complications |
| The Royal North Shore Emergency Airway Registry | Fogg et al., 2016 | Full-text | Australia (Sydney) | Single centre | 655(total) - 295 (pre-intervention) + 360 (post-intervention) | 2010-2011; 2012-2014 | ED only | All patients requiring endotracheal intubation in the ED ((7% paediatrics age <17 yearS) | Investigate whether changes made to endotracheal intubation in the centre's ED improved first pass success rate and reduced complication incidence |
|  | Vassiliadis et al., 2015 | Full-text | Australia (Sydney) | Multi-centre (2) | 601 (included) | 2011-2012 | ED only | All ED patients with endotracheal intubation attempt using Macintosh or Miller intubation device (% paediatrics not reported, mean age 53-55.7 years) | Compare direct and C-MAC video laryngoscopy in terms of first pass success rate, airway grade and complications |
|  | Annesley et al., 2012 | Abstract | Australia (Sydney) | Single centre | 295 (total, included) | 2010-2012 | Not specified | Not reported | Describe the practice of ED intubations in a tertiary hospital in Australia |

**ANZEDAR** The Australian and New Zealand Emergency Department Airway Registry, **BCARE** British Columbia Airway Registry for Emergencies, **DREAM** Defense Registry for Emergency Airway Management, **EDIR** Emergency Department Intubation Registry, **JEAN** Japanese Emergency Airway Network Registry 1 and 2, **KEAMR** Korean Emergency Airway Management Registry, **NEAR** National Emergency Airway Registry, **NERAA** National Emergency Resuscitation Airway Audit

Direct laryngoscopy (DL), video laryngoscopy (VL), emergency department (ED), emergency medicine (EM), first pass success (FPS), rapid sequence intubation (RSI)
