# Supplementary material for: Airway registries in primarily adult, emergent endotracheal intubation: a scoping review
Source: Scand J Trauma Resusc Emerg Med. 2023 Mar 8;31:11. doi: 10.1186/s13049-023-01075-z (PMC9993388; doi:10.1186/s13049-023-01075-z)
Supplement: Supplementary file 3 — Additional file 3. QI/QA Studies. [file 13049_2023_1075_MOESM3_ESM.docx]

Additional File 3: QI/QA Studies

| **Airway Registry** | **Author, Year of Publication** | **Abstract or Full Text** | **Aim of Study** | **Study Methodology** | **Study Population** | **Information Captured by Airway Registry** | **Intervention Type** | **Study Outcome Measures** | **Important Conclusions** |
| --- | --- | --- | --- | --- | --- | --- | --- | --- | --- |
| **QA Studies** | | | | | | | | | |
| ANZEDAR | Powell et al., 2018 | Full-text | Describe airway management practices in a rural New Zealand ED | Prospective observational | All ED patients requiring intubation | - Patient demographics - Indication for intubation - Difficult airway characteristics - Pre- and post-intubation vitals - Oxygenation - Patient positioning - Medications used - Operator seniority and level of experience - Use of pre-intubation checklist - Devices used - Intubation maneuvers used - Complications - Patient disposition |  | - Operator characteristics - Indication for intubation - Medications used - Device used - First pass success - Airway assessment - Patient age - Patient disposition - Adverse event rate | - Intubation is an infrequent procedure in this ED - First pass success rate is comparable to larger centres while complication rates were lower |
|  | Alkhouri et al., 2021 | Full-text | Describe management of cases resulting in emergency front-of-neck access | Retrospective analysis and case review | All ED patients undergoing emergency front-of-neck access |  |  | - Patient vital signs immediately before induction - Rate of hypoxia - Patient disposition | - Most cases deviated from standard difficult airway practice, which likely increased the need for emergency surgical airway |
| Cleveland Clinic Emergency Airway Registry | Phelan et al., 2011 | Full-text | Determine the rate at which confirmation of correct endotracheal tube (ET) placement is documented | Retrospective analysis | All ED patients requiring intubation | - Device used - Number of attempts - Attempt success or failure - Adverse events rate |  | - Documentation of correct ET placement | - Documentation is poor in the ED for patients who arrive intubated or transferred between hospitals - High rate (96%) of documentation for patients intubated in the ED |
| JEANI+II | Goto et al., 2014 | Full-text | Examine success rate of airway management by ED residents | Retrospective analysis | All ED patients requiring intubation managed by residents | - Patient demographics - Indication for intubation - Method used - Medications used - Operator specialty and level of experience - Number of attempts - Attempt success or failure - Adverse events rate |  | - Success rates - Operator level of training - Intubation method - Adverse event rate | - Steady improvement in success rate of both initial and rescue attempts over residency training - Lower reported first attempt success rate compared to previous studies |
|  | Goto et al., 2017 | Full-text | Investigate changes in Japanese ED practices and related outcomes over time (2010-2016) | Retrospective analysis | All ED patients requiring intubation |  |  | - First, second, and third attempt success rate - Overall success rate - Intubation method - Adverse event rate | - Centres implemented more state-of-the-art airway management practices - Increased overall success rates, particularly among residents - Increased RSI and VL use - No significant change in rate of adverse events |
| NEARI | Sagarin et al., 2003 | Full-text | Identify patterns of midazolam use in the ED and assess compliance with recommended dosages | Retrospective analysis | All ED patients intubated orally using RSI | - Patient demographics - Difficult airway characteristics - Presence of reduced neck mobility - Method used - Patient positioning - Devices used - Medications used - Operator characteristics - Attempt success or failure - Adverse events rate - Patient disposition - Immediacy of intubation |  | - Medications used - Rate of hypotension - Rate of altered patient mental status | - Midazolam, when used as the sole induction agent with RSI, was underdosed for the majority of patients - Underdosing not observed with other agents used only for anesthetic induction |
| NEARIII | April et al., 2017 | Full-text | Describe airway management practices at Brooke Army Medical Centre | Prospective observational | All ED patients requiring intubation |  |  | - Operator characteristics - First pass success - Device used Indication for intubation | - The military EM residency program provides residents with robust exposure to airway management opportunities - Outcomes, success rates, and adverse event rates are comparable to civilian centres (data from NEAR) |
|  | Kilgo et al., 2018 | Abstract | Describe airway management practices at this single centre and its comparison to data from NEAR | Prospective observational | All ED patients requiring intubation |  |  | - FPS rate - Indication for intubation - Operator characteristics - Medications used - Adverse events rate | - Etomidate was the most commonly used induction agent, similar to NEAR - Rocuronium was the most commonly used paralytic agent, unlike with NEAR, where the most common agent was succinylcholine - FPS rates and adverse events rates are similar to those reported in NEAR |
| South African ED registry | Hart & Goldstein, 2020 | Full-text | Analyze airway management characteristics in a South African centre, and its comparison to international data | Retrospective analysis | ED patients >18 years old requiring intubation | - Patient demographics - Patient preparation - Preload volume - Inotrope use - Methods used - Devices used - Use of pre-oxygenation - Ultimate success or failure - Operator level of training - Medications used - Vital signs - Complications - Cormack-Lehane view |  | - FPS rate - Devices used - Adverse events rate | - Success rates were comparable to international levels |
| The Royal North Shore Emergency Airway Registry | Annesley et al., 2012 | Abstract | Describe airway management practices in a tertiary Australian ED | Prospective observational |  | - Operator level of training - Number of attempts - Attempt success or failure - Devices used - Difficult airway characteristics - Medications used - Complications |  | - Operator characteristics - Number of attempts - Adjuncts used - Adverse event rate | - Success rate within two attempts is comparable to the literature - Rate of complications is high but comparable to data from EDs overseas - Rate of difficult laryngoscopy is much higher than the literature |
| **QI Studies** | | | | | | | | | |
| Cleveland Clinic Emergency Airway Registry | Phelan et al., 2010 | Full-text | Determine the utility of an airway registry to survey and evaluate ED airway management | Prospective observational | All ED patients requiring intubation | - Device used - Number of attempts - Attempt success or failure - Adverse events rate | Airway registry implementation | - Operator characteristics - First pass success - Overall success - Rate of RSI - Rate of cricothyrotomy | - Overall success rate was comparable to NEAR - Ability to set up an airway registry to audit ED practices is feasible - Data collected can be used to assess ED airway management for quality improvement purposes |
|  | Phelan et al., 2016 | Full-text | Evaluate whether the use of a multi-facetted intervention is associated with reduced mortality rates | Prospective observational cohort study | All ED patients requiring intubation |  | Implementation of interventions designed to improve EM physicians’ documentation rate of correct ET placement | - Patient mortality | - Interventions improved documentation rate of correct ET placement, which was associated with a decline in patient morality - Lack of verification of ET placement and failure to document confirmation poses a significant risk to patient safety |
| Continuous quality improvement database | Sakles et al., 2019 | Full-text | Describe the impact of a continuous airway quality improvement program on airway management | Retrospective analysis | All ED patients >18 years old that underwent RSI by an EM resident as the first operator | - Patient demographics - Trauma status - Failure of prehospital intubation - Difficult airway characteristics - Method used - Devices used - Reason for device selection - Medications used - Indication for intubation - Operator specialty and level of training - Number of attempts - Attempt success or failure - Technique used to confirm correct tracheal tube placement - Cormack- Lehane view | Airway registry implementation and  educational program implementation | - First pass success - First pass success without adverse events - Procedural characteristics - Adverse event rate | - The quality improvement program improved first pass success and decreased rate of adverse events - A small increase in the number of intubations performed by senior residents was observed |
| KEAMR | Kim et al., 2017 | Full-text | Evaluate the effectiveness of a simulation-based emergency airway management education program (SBEAMP) | Retrospective sub-group analysis | All non-cardiac arrest ED patients requiring intubation | - Patient demographics - Indication for intubation - Presence of a crash airway - Difficult airway characteristics - Glottis exposure grade - Number of attempts - Operator level of training and specialty - Method used - Devices used - Attempt success or failure - Adverse events rate | Educational program implementation | - Medications used - Use of pre-oxygenation - First pass success - Adverse event rate | - Ratio of patients intubated with no medications decreased over time, but decreased more rapidly in the SBEAMP group - Pre-oxygenation use was more prevalent in SBEAMP group - No significant difference in first pass success or failed airways was found between the groups |
| King Abdulaziz University Hospital Airway Registry | Bakhsh et al., 2021 | Full-text | Evaluate whether the implementation of a local airway registry and quality improvement program improved first pass success rate | Prospective observational | All ED patients requiring intubation | - Time of intubation - Patient demographics - Indication for intubation - Vital signs - Cormack-Lehane grade - Medications used - Method used - Devices used - Number of attempts - Operator level or training - Complications | Airway registry implementation and introduction of a quality improvement program | - First pass success - Adverse event rate | - First pass success rate improved significantly over study period - Laryngoscopy use and good Cormack- Lehane view were significant in predicted first pass success |
| Samsung Medical Centre Emergency Airway Program | Hwang et al., 2018 | Full-text | Evaluate the effectiveness of a quality improvement project | Prospective observational | All ED patients requiring intubation | - Patient demographics - Pre- and post-intubation vitals - Indication for intubation - Number of attempts - Devices used - Glottic opening score - Difficult airway characteristics - Operator level of experience and seniority - Medications used - Adverse events rate | Procedural standardization,  educational program implementation, and equipment upgrades | - First pass success - Multiple attempt rate - Adverse event rate | - Compliance with recommendations improved over the three years - FPS rate increased, adverse events rate decreased, and total number of attempts decreased since program implementation |
| Singapore General Hospital Emergency Airway Registry | Wong & Ho, 2006 | Full-text | Investigate the effect of PPE mandates and number of personnel restrictions on airway management during the SARS crisis | Retrospective analysis | All ED patients requiring intubation | - Patient demographics - Indication for intubation - Devices used - Method used - Difficult airway characteristics - Attempt success or failure - Number of attempts - Complications - Operator specialty and level of training - Rescue methods used - Patient disposition | Introduction of protective equipment policies and procedures | - Overall success rates - Operator characteristics - Adverse event rate | - The use of PPE, including powered air-purifying respirators, did not affect intubation success rate during the SARS outbreak - With personnel restrictions, experienced operators are more likely to perform the intubations |
| The Alfred Airway Registry | Groombridget et al., 2020 | Full-text | Evaluate whether a bundle of quality improvement initiatives improves first pass success and complication rates | Prospective cohort interventional | All ED patients requiring intubation | - Patient demographics - Indication for intubation - Glasgow coma score - Vitals - Operator specialty - Method used - Maneuvers used - Medications used - Devices used - Confirmation of placement - Patient disposition - Adjunct used - Number of attempts - Attempt success or failure | Airway registry implementation, introduction of audits, educational program implementation, and creation of cognitive aids | - First attempt success - Complication rates | - Targeted quality improvement initiatives were associated with improved first pass success and decreased adverse events |
|  | Groombridge et al., 2021 | Full-text | Identify the effect of COVID-19 modifications to intubation practices | Retrospective analysis | All ED patients requiring intubation | - Same as above, but modified to include PPE, intubation team size and intubation team makeup | PPE modification, risk reduction measures, increased supervision, airway registry modification | - First pass success - Rate of hypoxia | - Intubations during the COVID-19 era were associated with more hypoxia despite implemented measures - First pass success was not affected by COVID-19 measures |
| The Royal North Shore Emergency Airway Registry | Fogg et al., 2016 | Full-text | Evaluate whether new ED measures improved first pass success and complication rates | Prospective observational | All ED patients requiring intubation | - Patient demographics - Indication for intubation - Difficult airway characteristics - Pre- and post-intubation vitals - Oxygenation - Patient positioning - Medications used - Operator seniority and level of experience - Use of pre-intubation checklist - Devices used - Intubation maneuvers used - Complications - Patient disposition | Educational program implementation, protocol standardization, and minimum training requirement prior to intubation | - First pass success - Overall success rate - Adverse event rate | - New measures significantly improved first pass success rate - Majority of staff had incorporated the changes into their routine practice |

**ANZEDAR** The Australian and New Zealand Emergency Department Airway Registry, **BCARE** British Columbia Airway Registry for Emergencies, **DREAM** Defense Registry for Emergency Airway Management, **EDIR** Emergency Department Intubation Registry, **JEAN** Japanese Emergency Airway Network Registry 1 and 2, **KEAMR** Korean Emergency Airway Management Registry, **NEAR** National Emergency Airway Registry, **NERAA** National Emergency Resuscitation Airway Audit

Direct laryngoscopy (DL), video laryngoscopy (VL), emergency department (ED), emergency medicine (EM), first pass success (FPS), rapid sequence intubation (RSI)
