# Supplementary material for: Airway registries in primarily adult, emergent endotracheal intubation: a scoping review
Source: Scand J Trauma Resusc Emerg Med. 2023 Mar 8;31:11. doi: 10.1186/s13049-023-01075-z (PMC9993388; doi:10.1186/s13049-023-01075-z)
Supplement: Supplementary file 4 — Additional file 4. Research Studies. [file 13049_2023_1075_MOESM4_ESM.docx]

Additional File 4: Research studies

| **Airway Registry** | **Author, Year of Publication** | **Abstract or Full-text** | **Aims of Study** | **Study Methodology** | **Study Population** | **Information Captured by Airway Registry** | **Study Outcome Measures** | **Important Conclusions** |
| --- | --- | --- | --- | --- | --- | --- | --- | --- |
| ANZEDAR | Alkhouri et al., 2017 | Full-text | Describe airway management practices across Australasian EDs | Prospective observational | All ED patients requiring intubation | - Patient demographics - Indication for intubation - Difficult airway characteristics - Vital signs at time of decision to intubate and immediately after intubation - Patient positioning - Medications used - Operator seniority and level of training - Use of pre-intubation checklist - Devices and blades used Intubation maneuvers used - Complications - Patient disposition | - Character of participating EDs - Indication for intubation - Patient positioning - Operator level of training - Induction drugs used - Intubation success - Airway maneuvers used Complications - Vital signs immediately before induction - Rate of surgical airway - Rate of hypoxia - Patient disposition | - Intubation success and complications rate does not differ based on size or location of the ED, but are instead associated with operator seniority, use of video laryngoscopy, bougie use, and Cormack-Lehane grade I or II view |
|  | Arnold et al., 2021 | Full-text | Describe current airway management practices after a failed intubation attempt in Australian and New Zealand EDs; Explore factors associated with second attempt success | Retrospective analysis | ED patients intubated with multiple attempts |  | - Operator characteristics - Devices used - Airway maneuvers used - Predicted difficult airway characteristics | - A change in operator occurred in over half of failed first attempt intubation cases and was more likely to occur when the first attempt was undertaken by a novice operator - Multiple attempts are associated with increased risk of adverse events |
|  | Ferguson et al., 2019 | Full-text | Describe the incidence of ketamine use in Australasian EDs and evaluate whether there has been any increase in its use; Identify predictors of ketamine use | Retrospective analysis | ED patients >2 years old requiring intubation where an induction agent was used |  | - Ketamine use - Indication for intubation - Operator specialty - FPS rate | - Over the 5-year study period, there occurred a significant increase in the use of ketamine as an induction agent - Choice of ketamine use was driven by patient physiology and presenting condition |
|  | Fogg et al., 2015 | Abstract | Create a data pool of airway management practices in Australasian EDs for participating departments to use in self-improvement and descriptive studies | Prospective observational | Not reported |  | Not reported | - ANZEDAR is an important initiative that can be used for gathering and analyzing data for audit purposes |
|  | Freeman et al., 2021 | Full-text | Describe and compare the hemodynamic effects of propofol, ketamine, and thiopentone during RSI; Identify predictors of hemodynamic instability | Retrospective analysis | ED patients >16 years old requiring intubation where an induction agent (not etomidate) was used |  | - Induction medications and doses - Indication for intubation - Patient age - Pre- and post-induction vitals - Glasgow coma scale | - Ketamine was used most frequently in trauma cases, propofol for medical indications and thiopentone for head trauma cases - Propofol and ketamine are associated with dose-dependent post-intubation hypotension - Factors associated with post-intubation hypotension includes medication use, lower oxygen saturation, older age, higher heart rate, and a shock index > 0.9 |
|  | Perera et al., 2021 | Full-text | Investigate whether the use of nasal oxygen during efforts to secure a tube for apneic oxygenation during RSI decreases the incidence of desaturation; Examine the contribution of apneic oxygenation on the incidence of desaturation | Prospective observational | ED patients indicated for intubation for non-cardiac arrest reasons, who were not ventilated during the apneic period |  | - Desaturation to <93% | - Nasal oxygen during efforts to secure a tube apneic oxygen is associated with lower incidence of desaturation in RSI - Patients with anticipated difficult airways and those requiring pre-oxygenation via laryngeal mask were at greater risk of desaturation |
| BCARE | Botros et al., 2020 | Abstract | Compare intubation techniques, success, and complication rates between EM consultants and trainees | Prospective observational | ED patients intubated by EM physicians | - Operator specialty and level of training - Airway assessment prior to intubation - Devices used - Number of attempts - Attempt success or failure - Adverse events | - Pre-intubation airway assessment - Technique used - FPS rate - Number of attempts - Complication rates | - Trainees had a stronger preference for using VL as the first-line technique than staff physicians - Success rates between senior residents and staff were similar, but significantly higher than junior residents - Number of attempts and complication rate was similar in all levels of training |
|  | Liu et al., 2018 | Abstract | Describe the development and implementation of a new airway registry at a tertiary Canadian centre | Not reported | Not reported | - Operator seniority and discipline - Preparation, technique used - Airway maneuvers used - Cormack-Lehane view - Confirmation techniques - Complications - Difficult airway identification | Not reported | - An airway registry is useful for tracking intubation performance and identifying factors associated with adverse patient outcomes - The development and implementation of an airway registry requires multi-disciplinary collaboration, engagement, and user feedback |
|  | Yoo et al., 2018 | Abstract | Describe development of the BCARE network, an emergency intubation database at two tertiary care centres and one community hospital | Not reported | All patients requiring intubation outside the OR |  | - Adverse event rate | - The BCARE network is a valuable quality improvement tool and can be used to assess performance across hospitals - Most common indication for intubation were intracranial hemorrhage/stroke, seizures, and sepsis - VL was more commonly used than DL |
| Chulalongkorn Airway Registry | Saoraya et al., 2021 | Full-text | Explore the incidence, management, and outcomes of patients with difficult airway predictors in an ED | Retrospective analysis | ED patients requiring intubation who were assessed for signs of difficult airway | - Patient characteristic - Indication for intubation - Difficult airway characteristic - Method used - Medications used - Number of attempts - Glottic view, Operator level of experience - Complications | - Incidence of difficult airway predictors - Method used - Medications used - Devices used - FPS rates - Complications rates | - Difficult airway predictors were present in a majority of patients - Difficult airway predictors were associated with decreased use of neuromuscular blocking agents but were not associated with glottic view, FPS or complication rates |
| Cipto Mangunkusumo General Hospital airway registry | Sulistio et al., 2021 | Full-text | Describe emergency intubation characteristics and FPS rates at the Cipto Mangunkusumo General Hospital | Prospective observational | All ED patients requiring intubation | - Patient demographics - Indication for intubation - Difficult airway characteristic - Vital signs pre-intubation - Equipment used - Patient positioning - Airway laryngoscopy grades - Number of attempts - Attempt success or failure - Complications - Patient disposition | - FPS rate - Indication for intubation - Adverse events rate - Patient demographics - Pre-intubation vital signs | - There is increased involvement of non-anaesthetists in ED airway management - FPS and complications rate were comparable to overseas data, though the types of drugs used were different due to operator familiarity and availability |
| Cleveland Clinic Emergency Airway Registry | Good et al., 2017 | Abstract | Evaluate utilization trends of direct laryngoscopy (DL) vs. video laryngoscopy (VL) over a four-year period at the Cleveland Clinic | Retrospective analysis | All ED patients requiring intubation | - Device used - Number of attempts - Attempt success or failure - Adverse events | - Devices used and frequency of use - FPS rates - Adverse events rate | - Increased use of VL compared to DL in the four-year period, though there was no significant difference in FPS or adverse events rate |
| Continuous quality improvement database | Sakles et al., 2012 | Full-text | Compare the performance of the C-MAC video laryngoscope to the Macintosh direct laryngoscope in ED patients | Retrospective analysis | ED patients who underwent intubation with the C-MAC or MAC direct laryngoscope as the initial device | - Patient demographics - Trauma status - Failure of prehospital intubation - Difficult airway characteristic - Method used - Device used - Reason for device selection - Medications used - Indication for intubation - Operator specialty and level of training - Number of attempts - Attempt success of failure - Confirmation of correct endotracheal tube placement - Difficult airway characteristic - Cormack- Lehane view | - Overall successful intubation rate - FPS rate - Cormack-Lehane view - Rate of immediately recognized esophageal intubations | - C-MAC VL resulted in more successful intubations than DL - C-MAC has a higher success rate in patients with difficulty airway characteristics - C-MAC has improved Cormack-Lehane views and lower incidences of esophageal intubation than DL |
|  | Patanwala et al., 2014 | Full-text | Compare FSP rates between etomidate and ketamine use with RSI | Retrospective analysis | ED patients who underwent RSI, where ketamine or etomidate was used |  | - FPS rate | - Etomidate and ketamine use result in equivalent FPS rates |
|  | Sakles et al., 2014 | Full-text | Compare the learning curves for DL and GlideScope VL (GVL) | Retrospective analysis | ED patients >18 years old who underwent initial intubation attempt by an EM resident using DL or GVL |  | - FPS rate - Resident level of training | - PGY2 and 3 residents’ FPS rate improved after the first year with GVL but not with DL, suggesting that the DL learning curve is fairly flat while GVL's is fairly steep |
|  | Arcaris et al., 2015 | Abstract | Compare the performance of EM residents using a video-enabled Macintosh direct laryngoscope blade to a conventional Macintosh direct laryngoscope blade | Retrospective analysis | ED patients >18 years old who underwent RSI by an EM resident using a Mac video laryngoscope or DL |  | - FPS rate - Overall success rate | - EM residents had a higher FPS rate and were ultimately more successful when using a Mac VL compared to a Mac DL |
|  | Corn et al., 2015 | Abstract | Compare the effectiveness of the GlideScope video laryngoscope to a direct laryngoscope in patients with bloody airways | Retrospective analysis | ED patients >18 years old who underwent RSI with a standard GVL or DL |  | - FPS rate | - GVL had a higher FPS rate than DL for patients with bloody airways undergoing RSI in the ED |
|  | Dicken et al., 2016 | Abstract | Compare FPS rate of the C-MAC and GlideScope-Mac when used for VL and DL in the ED | Retrospective analysis | ED patients >18 years old who underwent RSI by an EM resident using the C-MAC or GVL-MAC |  | - FPS rate | - When used as a VL, the C-MAC and GVL-MAC results in similar FPS - When used as a DL, the C-MAC resulted in higher FPS rates |
|  | Mosier et al., 2013 | Full-text | Compare intubation success between the GlideScope video laryngoscope and C-MAC laryngoscopy in ED patients | Retrospective analysis | ED patients intubated with a GlideScope video laryngoscope or the C-MAC as the initial device |  | - FPS rate - Overall success rate | - No difference was found in either FPS or overall success rates between the C-MAC and GlideScope |
|  | Pacheco et al., 2021 | Full-text | Examine the association between physiologically and anatomically difficult airways using FPS without adverse events | Retrospective analysis | ED patients >18 years old who underwent RSI with DL or VL |  | - Rate of FPS without adverse events - Adverse events rate | - Anatomically and physiologically difficult airways demonstrated a similar decrease in FPS without adverse events - Standard geometry VL and hyperangulated VL use were associated with an increase in FPS without adverse events |
|  | Patanwala et al., 2011 | Full-text | Determine the effect of paralytic type and dose on first-attempt RSI success in the ED, and other factors associated with FPS | Retrospective analysis | ED patients >18 years old who underwent RSI, induced with etomidate or received succinylcholine or rocuronium as a paralytic agent |  | - FPS rate - Number of attempts - Operator experience - Presence of difficult airway characteristics - Device used - Laryngeal view, Patient characteristics | - No difference was found in FPS rate or number of attempts across paralytic type or dose used |
|  | Sakles et al., 2013 | Abstract | Determine the incidence of hypoxemia during RSI of head-injured patients in the ED | Retrospective analysis | ED patients who sustained blunt or penetrating trauma to the head who underwent RSI |  | - Hypoxemia rate | - There is a high rate of hypoxemic events during the intubation of head-injured patients |
|  | Sakles et al., 2016 | Full-text | Evaluate the clinical utility of the C-MAC as a direct laryngoscope and determine its impact on FPS rates compared to conventional Macintosh direct laryngoscope | Prospective observational | ED patients >18 years old who underwent RSI with the C-MAC or Mac direct laryngoscope used as the initial device |  | - FPS rate - Cormack-Lehane view - Hypoxemia rate | - Conventional Mac DL had a higher overall success rate than C-MAC as the initial device used - With C-MAC, operators were able to make mid-intubation switches to VL for a higher overall FPS rate - The incidence of hypoxemia were similar across the two devices |
|  | Sakles et al., 2013 | Full-text | Describe the association between multiple intubation attempts and incidence of adverse events during ED intubations | Retrospective analysis | All ED patients requiring intubation |  | - Adverse events rate | - Multiple intubation attempts is associated with a higher incidence of adverse events, including oxygen desaturation, aspiration, and esophageal intubation |
|  | Sakles et al., 2017 | Full-text | Determine the impact of a soiled airway on FPS and compare the success rate between the GlideScope and the direct laryngoscope in this context | Prospective observational | ED patients >18 years old who underwent RSI by an EM resident using the reusable GlideScope or a direct laryngoscope |  | - FPS rate - Overall success rate with initial device used - Degree of lens contamination | - Soiled airways (eg. blood, vomitus) are associated with reduced FPS rates regardless of the device used - GlideScope resulted in higher overall success rates in patients with soiled airways than DL - After a failed attempt with a soiled airway, the GlideScope successfully rescued DL more frequently than the converse |
|  | Sakles et al., 2014 | Full-text | Compare the FPS and clinical performance characteristics of GVL and Cobalt GlideScope video laryngoscope (cGVL) | Retrospective analysis | ED patients >18 years old where the GVL or cGVL with a stylet was used for the initial intubation attempt |  | - FPS rate - Ultimate success rate with initial device used - Degree of lens contamination - Degree of lens fogging | - The standard GVL had a higher FPS and overall success rate than the disposable cGVL - The cGVL had a significantly higher incidence of fogging and lens contamination |
|  | Sakles et al., 2012 | Full-text | Compare the performance of GVL and DL in the ED | Retrospective analysis | ED patients requiring intubation where a GlideScope video laryngoscope or a traditional laryngoscope was used |  | - FPS rate - Overall success rate - Key performance characteristics - Reasons for failures | - Use of GVL achieved a higher FPS rate than DL for ED intubations |
|  | Sakles et al., 2015 | Full-text | Compare the incidence of esophageal intubations when EM residents use DL vs. VL during intubation attempts in the ED | Retrospective analysis | ED patients who underwent RSI by an EM resident using DL or VL |  | - Rate of esophageal intubations - Adverse events rate | - Use of VL by EM residents was associated with a significant reduction in the incidence of esophageal intubations compared to DL - Inadvertent esophageal intubations was associated with a higher rate of adverse events |
|  | Sakles & Kalin, 2012 | Full-text | Compare the intubation success rates between the GlideScope GlideRite rigid stylet and a standard malleable stylet | Retrospective analysis | ED patients requiring intubation where the GlideScope Standard, Gobalt, or Ranger was used as the initial device |  | - FPS rate - Overall success rate | - Use of GlideScope GlideRite rigid stylet was associated with higher overall success rates than use of a standard malleable stylet for GlideScope VL - GlideScope GlideRite rigid stylet was associated with lower complication rates, primarily the incidence of oxygen desaturation |
|  | Sakles et al., 2015 | Full-text | Compare the CMAC with a direct laryngoscope to determine success rates when used for a rescue attempt after a failed initial intubation attempt | Retrospective analysis | ED patients >18 years old intubated with multiple attempts, all by the same EM physician, who used C-MAC or DL for the second attempt |  | - Rate of successful rescue intubation on second attempt by the initial operator | - After a failed first intubation attempt, EM physicians were more successful on their second attempt when using the CMAC compared to the DL, regardless of which device was used initially |
|  | Sakles et al., 2016 | Full-text | Determine the effect of apneic oxygenation on FPS without hypoxemia | Prospective observational | ED patients >18 years old who underwent RSI by an EM resident, with a starting oxygen saturation of >=90% |  | - Rate of FPS without hypoxemia | - Use of apneic oxygenation during RSI is associated with an increase in FPS without hypoxemia. - Apneic oxygenation was only used in 60% of patients undergoing RSI |
|  | Sakles et al., 2016 | Full-text | Determine the effect of apneic oxygenation on oxygen desaturation during RSI of patients with intracranial hemorrhage in the ED | Prospective observational | ED patients >18 years old with an intracranial hemorrhage who underwent RSI by an EM resident, with a starting oxygen saturation of >90% |  | - Hypoxemia rate | - Apneic oxygenation is associated with a significant reduction in oxygen desaturation during RSI for patients with intracranial hemorrhage |
|  | Sakles et al., 2014 | Full-text | Compare the efficacy of VL to DL on FPS of patients with difficult airway characteristics | Retrospective analysis | ED patients >18 years old with a difficult airway and attempted intubation by an EM physician using VL or DL |  | - FPS rate | - VL had a greater FPS rate than DL for patients with difficult airway characteristics |
|  | Sakles et al., 2013 | Abstract | Determine if operator-identified patient obesity is associated with reduced FPS rates | Prospective observational | All ED patients requiring intubation |  | - FPS rate | - Obesity is associated with a significant reduction in FPS rate - Identification of obese patients may help operators choose the appropriate intubation methods and devices to improve FPS |
| DREAM | Mendez et al., 2021 | Full-text | Describe pilot data collected as part of developing the Defense Registry for Emergency Airway Management (DREAM) | Prospective observational | All ED patients requiring intubation | - Patient demographics - Indication for intubation - Methods used - Operator characteristic - Number of attempts - Attempt success of failure | - FPS rate - Devices used - Indication for intubation - Difficult airway characteristics - Operator level of training | - Most intubations were indicated due to trauma - The most frequently encountered difficult airways included blood in the airways and facial trauma - Most intubations were performed with VL, which resulted in a high FPS rate that was comparable to other studies |
| EDIR | Kerslake et al., 2015 | Full-text | Describe airway management practices at the Royal Infirmary of Edinburgh  within the EDIR | Prospective, observational | All ED patients requiring intubation | - Patient demographics - Pre-induction vitals - Difficult airway characteristic - Time of intubation - Use of pre-oxygenation Use of cricoid pressure - Medications used - Operator characteristic - Supervisor characteristic - Cormack- Lehane view - Adjuncts used Complications | Not reported | - More than 50% of patients had a medical diagnosis requiring intubation - Use of RSI was associated with a higher FPS rate |
|  | Hale et al., 2017 | Full-text | Describe airway management practices and outcomes across age ranges | Retrospective analysis | All ED patients requiring intubation |  | - Adverse events rate | - Adolescents had low intubation frequencies and were associated with higher FPS and lower adverse events rates - Adolescent airways can be safely managed in adult ED with close collaboration between EM and anaesthesia staff |
|  | Donald, 2011 | Full-text | Evaluate the RSI training and experience attained as a specialist registrar in a Scottish teaching hospital ED and whether it's sufficient to progress to independent practice | Retrospective analysis, followed by qualitative surveys | ED patients who underwent RSI by an EM resident using DL or VL |  | - Adverse events rate | - Trainees were satisfied with the RSI training they received - Additional training may be necessary to improve confidence in performing pre-hospital RSI - An EM consultant led airway service with the support of the anaesthesia department is essential for delivering RSI training and supervision |
|  | Graham et al., 2003 | Full-text | Evaluate RSI practices performed by EM physicians or anesthesiologists in a sample of EDs in Scotland | Prospective observational | ED patients who underwent RSI |  | - FPS rate - Complications Laryngoscopic view - Adverse event rate | - Anaesthetists have a higher FPS rate and obtained more grade I and II laryngoscopy views than EM physicians - EM physicians had a higher proportion of patients who experienced immediate complications compared with anaesthetists, though not statistically significantly - EM physicians perform RSI on a higher proportion of patients with physiological compromise and within 15 minutes of arrival to the ED than anaesthetists |
|  | Paul et al., 2012 | Full-text | Determine long-term survival rates in critically ill patients undergoing intubation without medication assistance | Prospective observational | ED patients >18 years old who underwent intubation with no medication administered (all those who underwent RSI were excluded) |  | - 12-month survival - Location of discharge - Length of stay | - Most common indication for intubation was loss of consciousness and respiratory failure - Non-medicine-assisted laryngoscopy was associated with higher first pass failure in patients with intact airway reflexes - Administration of a neuromuscular blocking agent was associated less attempts and fewer complications |
|  | Reid et al., 2011 | Full-text | Determine the frequency and primary indication for surgical airway during ED intubation | Prospective observational | ED patients >13 years old requiring intubation |  | - Surgical airway rate - Indication for surgical airway - Survival to hospital discharge | - Rate of surgical airways during the study period was 0.2% - All surgical airways were performed after failed intubation attempts and had 100% success rates on first attempt - Most common indication for surgical airways was trauma |
|  | Stevenson et al., 2007 | Full-text | Characterize airway management practices in a Scottish ED over 40 months | Prospective observational | All ED patients requiring intubation |  | - FPS rate - Complication rate | - EM physicians and anesthetists had comparable success and complication rates - RSI can be performed safely in the district hospital ED |
| JEANI+II | Imamura et al., 2013 | Full-text | Characterize airway management for ED geriatric patients in Japan | Retrospective analysis | ED patients >18 years old requiring intubation | - Patient demographics - Indication for intubation - Method used - Medications used - Operator specialty and level of training - Number of attempts - Attempt success or failure - Adverse events | - Indication for intubation - Methods used - Devices used - Medications used - Operator characteristics - Overall success rates - Adverse events | - Geriatric patients had higher success rates compared to younger patients - There were no significant differences in adverse event rates |
|  | Nakao et al., 2015 | Full-text | Describe the current airway management practices for trauma patients in Japanese EDs | Prospective observational | ED patients requiring intubation due to trauma |  | - Indication for intubation - Initial method used - FPS rate - Overall success rate - Adverse events rate | - Japanese EDs have an acceptable overall success rate for airway management - There is a lot of variability across Eds in the initial method of intubation used, success rates, and adverse event rates for trauma airway management |
|  | Yamanaka et al., 2020 | Full-text | Determine the relationship between multiple intubation attempts in the ED and patient morbidity and mortality during hospitalization | Retrospective analysis | ED patients >18 years old requiring intubation |  | - Patient mortality and morbidity - Number of attempts | - Multiple intubation attempts was not associated with increased mortality or morbidity during hospitalization - DL was the most frequently used first device - Use of medications was associated with a higher change of FPS - Junior residents had lower FPS rates than senior physicians |
|  | Okubo et al., 2017 | Full-text | Investigate intubation success and complication rates with the use of RSI compared to non-RSI methods in the ED | Retrospective analysis | ED patients who underwent intubation with medications administered, and where a consistent method was used across all attempts |  | - FPS rate - Second pass success rate - Adverse events rate | - Use of RSI was associated with higher first and second attempt success rates - No significant difference was found in complication rates between RSI and non-RSI methods |
|  | Goto et al., 2015 | Full-text | Determine whether repeated intubation attempts by the same operator had better success rates than by multiple operators | Retrospective analysis | ED patients successfully intubated after multiple attempts |  | - Second and third attempt success rates | - Success rate by a single operator significantly declined with increased numbers of attempts - A change in operator for the second attempt was associated with a higher success rate |
| KEAMR | Cho et al., 2015 | Full-text | Evaluate factors affecting FPS in trauma patients | Prospective observational | Trauma ED patients requiring intubation | - Patient demographics - Indications for intubation - Presence of a crash airway - Difficult airway characteristic - Glottis exposure grade - Number of attempts - Operator specialty and level of training - Method used - Device used - Attempt success or failure - Adverse events | - FPS rate | - Factors associated with greater FPS rates for difficult airway trauma patients includes operator specialty and level of training, and use of VL |
|  | Kim et al., 2013 | Full-text | Identify factors associated with successful second and third attempts after failed first intubation attempts in the ED | Retrospective analysis | ED patients >18 years old who received advanced airway management after a failed first attempt |  | - Rate of successful rescue attempts | - Factors associated with success second attempts includes non-difficult airways, use of RSI, and a senior EM physician as the operator - Non-difficult airways and use of RSI was associated with successful third attempts |
|  | Choi et al., 2015 | Full-text | Compare the use of GlideScopeVL with Macintosh laryngoscopy for improving FPS | Retrospective analysis | ED patients >18 years old requiring intubation |  | - FPS rate - Rate of intubation failure | - GVL and MAC did not differ in terms of FPS rates |
|  | Cho et al., 2013 | Full-text | Evaluate current practices and complications of emergency airway management in Korean elderly patients | Retrospective analysis | ED patients >65 years old requiring intubation |  | - Airway assessment factors - RSI performance - FPS rate - Complications rate | - Rate of difficult airways was lower in elderly patients compared to younger patients - Rate of RSI attempts, FPS, and complications was similar across all age groups |
|  | Kim et al., 2011 | Abstract | Compare intubation FPS rate between the GlideScope video laryngoscope and Macintosh laryngoscope during CPR | Prospective observational | ED patients >18 years old with out-of-hospital cardiac arrests requiring intubation |  | - FPS rate - Rate of intubation failure | - No difference was found in FPS rates between GVL and MAC use - GVL use was associated with a lower failure rate than MAC |
|  | Lee et al., 2016 | Full-text | Characterize the change in GlideScope VL and DL usage frequency and FPS rate | Retrospective analysis | ED patients >18 years old who underwent intubation by an EM physician without the use of surgical methods or extraglottic devices on the first attempt |  | - FPS rate | - Across the study period, the rate of RSI use increased, use of GVL increased, and FPS rate for DL decreased while FPS rate for GVL increased - Level of training of the first operator was not associated with success rate |
| Middlemore Hospital Airway Registry | Brainard et al., 2014 | Poster | Describe the implementation of an airway registry at a single large ED in Auckland | Prospective observational |  | - Patient demographics - Indication for intubation - Method used - Device used - Patient positioning - Use of pre-RSI checklist - Medications used - Number of attempts - Operator specialty and level of training - Confirmation of endotracheal tube placement - Intubation maneuvers used - Complications - Patient disposition - Difficult airway characteristics | - Percent completion of airway registry forms - Time taken for form completion - Time taken for form entry - Indications for intubation | - Implementation of an airway registry at a single institution is feasible |
| NEARI | Delorio, 2005 | Full-text | Evaluate the use of end-tidal carbon dioxide monitoring in non-cardiac arrest patients within EDs | Retrospective analysis and qualitative surveys | ED patients requiring intubation not due to cardiac arrest | - Patient demographics - Difficult airway characteristic - Presence of reduced neck mobility - Method used Patient positioning - Device used - Medications and doses used - Operator characteristic - First attempt success or failure - Ultimate success or failure - Adverse events - Immediacy of intubation | - Use of end-tidal carbon dioxide monitoring to confirm endotracheal tube placement - Use of qualitative methods to confirm endotracheal tube placement | - Most physicians did not use the quantitative monitoring methods provided |
| NEARII | Sagarin et al., 2005 | Full-text | Examine the success of airway management by emergency medicine residents across North America | Retrospective analysis | All ED patients requiring intubation |  | - FPS rate - Success rate by the initial operator | - EM residents have a high degree of success with airway management, with improvement seen from PGY1 to PGY3 residents - Residents are successful 2/3 of the time on second attempts - EM residents performed rescue intubations with similar success rates to initial intubation attempts - RSI was the most commonly used method amongst residents |
|  | Blair et al., 2002 | Full-text | Describe the prevalence of ED airway management failures requiring rescue maneuvers and characterize successful rescue methods used; describe the role of emergency physicians and other specialists in rescue airway management | Retrospective analysis | ED patients requiring more than one course of intubation |  | - Method used - Operator specialty - Adjuncts used - Number of attempts | - RSI is the most common rescue maneuver used - Invasive airway techniques are still important and used, though only in a small percentage of cases - Airway adjuncts are not commonly used |
|  | Walls et al., 2011 | Full-text | Describe airway management practices across participating EDs | Prospective observational | All ED patients requiring intubation |  | - Indications for intubation - Method used - Device used - Medications used - Overall success rates - Operator specialty - Adverse events rate | - Most common indications for intubation include cardiac arrest, overdose, congestive heart failure, and coma - Neuromuscular blockade was used in most cases - An overall success rate of 99% was observed across all EDs |
|  | Collins et al., 2005 | Abstract | Evaluate airway management practices for patients with severe asthma in the ED setting | Prospective observational | All ED patients requiring intubation due to trauma |  | - Intubation frequency - Methods used - Medications used - Operator specialty and level of training - Overall success rate - Complications | - RSI with DL was the most common intubation method used - Etomidate was the most commonly used induction agent and succinylcholine was the most commonly used paralytic - An overall success rate or 98.8% was observed across all EDs |
|  | Walls et al., 1999 | Abstract | Characterize the success and complication rates of US emergency airway management | Prospective observational | All ED patients requiring intubation |  | - Overall success rate - Number of attempts - Complications rate | - EM physicians performed most intubations, with RSI as the most common method - Use of RSI was associated with higher success rates |
| NEARIII | Roy et al., 2019 | Abstract | Evaluate the association between intubation methods with adverse events and FPS | Retrospective analysis | All ED patients requiring intubation |  | - Method used - Device used - Medications used - FPS rate - Adverse events rate | - VL is more commonly used than DL - Higher adverse events were observed in medical patients and with ketamine use - VL was associated with a higher FPS rate |
|  | Joshi et al., 2020 | Abstract | Determine the impact of deep sedation immediately following ED intubation on mortality | Retrospective analysis | ED patients >18 years old requiring intubation |  | - Richmond Agitation-Sedation Scale (RASS) score of sedation used - Patient mortality | - Propofol was the most common sedative used - No statistically significant difference in mortality was found between deep and light sedation groups |
|  | April et al., 2021 | Full-text | Examine the prevalence of peri-intubation cardiac arrest and its association with case features in the ED setting | Retrospective analysis | ED patients >14 years old requiring intubation |  | - FPS rate - Ultimate success rate - Peri-intubation cardiac arrest rate - Adverse events rate - Patient disposition | - Increased risk of peri-intubation cardiac arrest was associated with pre-intubation SBP < 100mmHg and pre-intubation oxygen saturation < 91% |
|  | Brown et al., 2014 | Full-text | Analyze performance attributes and identify evolving trends in ED intubation practices | Retrospective analysis | ED patients >14 years old requiring intubation |  | - FPS rate - Devices used - Medications used - Method used - Indication for intubation - Complications rate | - Trainee intubation success rates has improved compared to previous registry data - Most failed airways were with trauma patients rather than medical patients - During the study period, an increased use of GlideScope and C-MAC or V-MAC was observed, which improved glottic visualization and intubation success - During the study period, use of etomidate for RSI and use of rocuronium increased dramatically - EM physicians predominantly use RSI and are able to successfully manage the majority of ED patients |
|  | Chan et al., 2021 | Full-text | Characterize airway management practices in the ED at the National University Hospital in Singapore | Prospective observational | ED patients >21 years old requiring intubation |  | - FPS rate - Devices used - Medications used - Method used - Complications rate | - All difficult airway predictors, other than facial trauma, showed good correlation with glottic exposure with VL - The majority of patients underwent RSI with etomidate and succinylcholine - The most commonly used device was the C-MAC video laryngoscope - The most frequently used sedation and analgesic medications were propofol and fentanyl |
|  | Driver et al., 2021 | Full-text | Evaluate FPS and adverse events for patients who underwent intubation using only ketamine, topical anesthesia, and RSI approaches | Retrospective analysis | ED patients >14 years old who underwent RSI, where ketamine was used as the only sedating agent, without NMBA or topical anesthesia facilitation |  | - FPS rate - FPS rate for airways with 1+ difficult airway characteristics - Rate of FPS without adverse events - Proportion of patients with Cormack-Lehane grade 1 or 2 on first attempt - Change in approach after failed first attempt - Hypoxemia rate - Adverse events rate | - Ketamine is rarely used and was associated with lower success rates and higher adverse events rates than other airway management strategies |
|  | Godwin et al., 2020 | Full-text | Describe current airway management techniques, devices, and outcomes for patients undergoing intubation for severe asthma | Retrospective analysis | ED patients requiring intubation due to asthma |  | - Incidence of intubation for asthma - Methods used - Medications used - Device used - Overall success rate - Adverse events rate | - Most patients requiring intubation for asthma underwent RSI after pre-oxygenation with BPAP and ketamine induction - Overall success rate in this population is 100% |
|  | Kaisler et al., 2021 | Full-text | Describe awake intubation practices in the ED | Retrospective analysis | ED patients who underwent awake intubation (defined as use of topical airway anesthesia only or with sedation) as the first attempt |  | - Devices used - Medications used - Methods used - Operator characteristics - FPS rate - Adverse events rate | - Awake intubations were most commonly performed in the setting of airway edema or obstruction - FPS rates for awake intubations were comparable to overall rates with DL but were lower than with VL - Rate of adverse events was higher than in the overall NEAR population - Flexible endoscopy and VL were the most commonly used techniques |
|  | Maddry et al., 2018 | Full-text | Characterize airway management practices at participating centres | Retrospective analysis | All ED patients requiring intubation |  | - FPS rate - Cormack-Lehane view - Adverse events rate - Lowest peri-intubation oxygen saturation | - Most intubation indications were related to trauma - FPS rate was similar to that reported in ED centres nationwide - The military centre provides trainees with adequate and comparable training to other EDs |
|  | Goldberg et al., 2018 | Abstract | Assess impact of using bougie on both FPS and ultimate success for difficult airways; evaluate the differential improvement in intubation success based on blade shape | Retrospective analysis | ED patients >15 years old requiring intubation |  | - FPS rate - Rate of bougie use - Bougie use in predicted difficult airways - Devices used | - Use of a hyperangulated blade with bougie was associated with a much lower FPS rate compared to use of a traditional blade with bougie |
|  | April et al., 2018 | Full-text | Compare FPS between succinylcholine and rocuronium use for RSI in the ED | Retrospective analysis | ED patients >14 years old who underwent intubation with succinylcholine or rocuronium |  | - FPS rate - Best Cormack-Lehane view - Adverse events rate - Lowest peri-intubation oxygen saturation | - No association between choice of paralytic and first-pass intubation success, glottic view, or adverse events |
|  | April et al., 2020 | Full-text | Compare patient outcomes between ketamine and etomidate use during ED intubations | Retrospective analysis | Normotensive ED patients >14 years old who underwent RSI, where ketamine or etomidate was used |  | - Peri-intubation hypotension - Mean systolic blood pressure nadir - Treatment for peri-intubation hypotension - FPS rate - Cormack-Lehane view - Adverse events rate | - Ketamine use was associated with higher peri-intubation hypotension compared to etomidate |
|  | Brown et al., 2007 | Abstract | Evaluate the performance of the DCI II video laryngoscope in the ED setting, especially compared to DL | Prospective observational | Convenience ED patients who underwent intubation with VL |  | - Rate or grade I (full) glottic view | - The DCI II video laryngoscope improves glottic exposure nearly a full grade in patients with partial or no view with DL - A full view of the vocal cords occurred more commonly with VL |
|  | Brown et al., 2020 | Full-text | Compare unaided VL with combinations of augmented DL | Retrospective analysis | ED patients >14 years old who underwent intubation with unassisted VL or augmented DL |  | - FPS rate - Adverse events rate | - VL used without any augmenting maneuver, device, or technique results in higher FPS rates than DL with augmentation - VL results in fewer esophageal intubations |
|  | Driver et al., 2020 | Full-text | Compare outcomes of patients intubated with a standard-geometry video laryngoscope with those using a hyperangulated video laryngoscope | Retrospective analysis | ED patients >14 years old who underwent intubation with a standard-geometry or hyperangulated video laryngoscope on the first attempt |  | - FPS rate - Rate of FPS without adverse events - Proportion of patients with Cormack-Lehane view of grade 1 or 2 on the first attempt - Devices used - Hypoxemia rate - Lowest oxyhemoglobin saturation - Adverse events rate | - No association was found between blade shape and FPS rate, with and without adverse events |
|  | Kunzler et al., 2021 | Abstract | Quantify the frequency of peri-intubation adverse events for patients intubated in the ED for overdose, and determine whether FPS without adverse events differ from patients with other indications | Retrospective analysis | ED patients >14 years old requiring intubation |  | - FPS rate - Rate of FPS without adverse events - Rate of rescue surgical airways - Adverse events rate | - FPS rates were similar between patients intubated for overdose compared to other indications - Overdose patients had a higher rate FPS without adverse events |
|  | Lembersky et al., 2020 | Full-text | Examine rates and associated factors of post-intubation sedation | Retrospective analysis | ED patients who underwent intubation with sedatives or analgesic medications |  | - Indication for intubation - Method used - Adverse events rate | - Post-intubation sedation rates were higher than previously reported - Factors that predispose use of sedation includes use of RSI and succinylcholine - Factors associated with decreased odds of receiving sedation includes pre- and post-hypotension |
|  | Levin et al., 2021 | Full-text | Assess the relationship of escalating weight-based dosing for rocuronium on FPS and peri-intubation adverse events | Retrospective analysis | ED patients >14 years old who underwent RSI with rocuronium (between 0.5-2mg/kg dose) |  | - FPS rate - Cormack-Lehane view - Adverse events rate - Lowest peri-intubation oxygen saturation | - Rocuronium doses of 1.4mg/kg or greater was associated with higher FPS rates among patients with pre-intubation hypotension, particularly when using DL - Adverse event rates similar across all doses. |
|  | Mohr et al., 2020 | Full-text | Describe the induction agents used for sepsis patients requiring intubation in the ED; compare peri-intubation adverse events between etomidate and ketamine use | Prospective observational | ED patients requiring intubation due to sepsis |  | - Medications used - FPS rate - Overall success rate - Adverse events rate | - Etomidate is the most commonly used induction agent amongst sepsis patients - Sepsis patients have a greater risk of adverse events post-intubation, including hypotension requiring a vasopressor - Ketamine was used more frequently among sepesis patients than patients without sepsis - Ketamine use was associated with a higher incidence of hypotension compared to etomidate use amongst sepsis patients |
|  | Monette et al., 2019 | Full-text | Evaluate the impact of VL on the clinical learning environment of EM residents | Retrospective analysis | ED patients requiring intubation at sites with an EM training program, where a direct laryngoscope, the C-MAC, or the GlideScope was used |  | - FPS rate - Devices used - Failed intubations in which a trainee had a second opportunity to perform an intubation | - FPS rates improved with increasing residency training years for both GlideScope and C-MAC - PGY 1 residents, but not PGY2 residents are more likely to receive a second attempt if the failed first attempt was performed with VL - No evidence that EM physicians are losing DL skills in the setting of increased VL use was found - Residents have a higher rate of FPS when using C-MAC compared to DL |
|  | Ruderman et al., 2018 | Abstract | Compare FPS rates between VLand DL in patients with predicted difficult airways | Retrospective analysis | ED patients with difficult airway characteristics requiring intubation, where DL or VL was used |  | - FPS rate - Indication for intubation - Patient characteristics - Peri-intubation hemodynamics - Oxygenation status - Adverse events rate | - VL was used twice more often than DL, with a higher FPS rate, including for patients with anticipated difficult airways |
|  | Nikolla et al., 2021 | Full-text | Compare post-induction hypoxemia and other adverse events between the ramped and supine positions in ED intubations with apneic oxygenation | Retrospective analysis | ED patients >18 years old requiring intubation for non-trauma and non-cardiac arrest indications |  | - Post-induction hypoxemia rate - FPS rate - Rate of FPS without adverse events - Cormack-Lehane view - Adverse events rate | - No association between position (ramped or supine) and post-induction hypoxemia with either DL or VL was found - No difference in post-induction hypoxemia with ramped position was found across body habitus subgroups |
|  | Garcia et al., 2014 | Abstract | Compare the learning curve of EM trainees of DL, hyperangulated blade VL, and standard geometry blade VL | Retrospective analysis | ED patients who underwent intubation by an EM trainee, where a device was used |  | - FPS rate - Devices used | - EM trainee proficiency with each type of laryngoscope was greatest at the PGY3+ level of training |
|  | Sandefur et al., 2021 | Full-text | Characterize airway management practices in the ED for patients with angioedema | Retrospective analysis | ED patients requiring intubation due to angioedema |  | - FPS rate - Adverse events rate | - The FPS rate for patients with angioedema is 81%, across a range of devices and techniques - RSI was the most common method used for patients with angioedema |
|  | Stoecklein et al., 2019 | Full-text | Compare the FPS rate, peri-intubation adverse events, and Cormack-Lehane laryngeal view of patients undergoing intubations in supine and nonsupine positions | Retrospective analysis | ED patients >18 years old who underwent RSI and have recorded peri-intubation positioning data available |  | - FPS rate - Glottic view - Adverse events rate | - Most VL and DL intubations were performed with patients in the supine position - Patients with morbid obesity and predicted difficult airways were more likely to be intubated in a non-supine position - FPS and overall glottic views were similar between groups, with more adverse events in the non-supine group. |
|  | Trent et al., 2021 | Full-text | Characterize airway management practices for trauma patients and identify factors associated with FPS | Retrospective analysis | ED patients requiring intubation due to trauma |  | - FPS rate | - A dramatic increase in the use of VL during the study period was associated with increased FPS in trauma patients - VL was associated with twice the odds of FPS compared to DL |
|  | Watase et al., 2020 | Abstract | Analyze the association between FPS and glottic view with hyperangulated VL compared to DL | Retrospective analysis | ED patients >15 years old who underwent intubation with DL or hyperangulated VL on the first attempt |  | - FPS rate - Glottic view | - Cormack-Lehane grade 3 was associated with higher FPS with hyperangulated VL compared to DL - All other grades showed no difference between devices |
|  | Van Oeveren et al., 2017 | Full-text | Describe telemedicine-assisted intubation practices in rural EDs served by large ED networks | Prospective observational | ED patients requiring intubation where telemedicine was activated during intubation attempts |  | - FPS rate - Ultimate success rate - Indication for intubation - Operator characteristics - Methods used - Medications used - Adverse events rate | - Telemedicine-enabled intubation performed in rural hospitals is feasible - Telemedicine consultation was most often requested after failed intubation attempts had failed - Adverse event rates were similar to those reported in other studies |
|  | Runde et al., 2020 | Abstract | Determine the association between the frequency of VL use and FPS rates amongst junior trainees | Retrospective analysis | All ED patients requiring intubation |  | - Proportion of first and second attempt intubations performed by PGY1 residents | - VL use was associated with higher FPS rates for PGY1 residents during first and second attempt intubations |
|  | Runde et al., 2019 | Abstract | Compare peri-intubation adverse event rates between VL and DL in the ED, stratified by intubator level of training | Retrospective analysis | All ED patients requiring intubation |  | - Adverse events rate | - VL and DL had similar rates of adverse events - Adverse event rates did not differ across operator level of training - VL was associated with lower incidences of esophageal intubation, vomiting with aspiration, and mainstem intubation |
|  | Hayden et al., 2018 | Full-text | Characterize the use of flexible fibreoptic intubation (FFI) in EDs | Prospective observational | ED patients >15 years old who underwent FFI |  | - FPS rate - Overall success rate - Adverse events rate | - FFI was most commonly used in the setting of airway obstruction - FFI use for airway obstruction had lower success rates and a higher need for surgical rescue compared to all other intubations - Over 40% of FFIs were unsuccessful in their first attempt with either VL or DL - FFI has a wide range of uses |
|  | Carlson et al., 2015 | Full-text | Compare intubation outcomes between patients with GI bleeds managed with VL vs. DL | Retrospective analysis | ED patients >14 years old requiring intubation due to GI bleed |  | - FPS rate - Cormack-Lehane view - Change in devices used | - For patients with GI bleeding, DL and VL had similar success rates, glottic views, and need to change devices |
|  | Brown et al., 2019 | Abstract | Characterize the frequency, risk factors, and outcomes of cricothyrotomies | Retrospective analysis | All ED patients requiring intubation |  | - FPS rate - Cricothyrotomy technique used - Risk factors for requiring cricothyrotomy | - All cricothyrotomies were performed with an open surgical technique - Success rates were similar regardless of bougie or no-bougie use - Risk factors for surgical airways include trauma, airway obstruction, and difficult airway characteristics - Cricothyrotomies in the ED are uncommon and rates are lower than those previously recorded |
|  | Nikolla et al., 2022 | Full-text | Compare the FPS rate between hyperangulated and standard geometry VL during ED intubations in non-supine positions | Retrospective analysis | ED patients >17 years old who underwent intubation in a non-supine position (ramped or upright) |  | - FPS rate | - Hyperanguated and standard geometry blades for VL had similar outcomes, including FPS and FPS without adverse events, in both the ramped and upright patient position |
| NERAA | Umana et al., 2022 | Full-text | Describe emergency airway management practices of critically unwell patients presenting to Irish EDs | Prospective observational | ED patients >16 years old requiring emergency airway management | - Patient demographics - Indication for intubation - Method used - Medication and dose used - Operator specialty and level of training - Number of attempts - Attempt success or failure - Complications | - Indication for intubation - Medications used - Complications rate | - Most common medical indication for intubation was cardiac arrest - Most common drugs used were propofol and rocuronium |
| Samsung Medical Centre Emergency Airway Program | Hwang et al., 2018 | Full-text | Evaluate the usefulness of C-MAC as a training tool for DL in the ED | Retrospective analysis | ED patients >18 years old who underwent intubation by an EM resident using conventional DL or C-MAC | - Patient demographics - Pre- and post-intubation vitals - Indication for intubation - Number of attempts - Devices used - Glottic opening score - Difficult airway characteristic - Complications - Operator level of training - Medications used | - FPS rate - Number of attempts - Adverse events rate | - C-MAC use was associated with greater FPS, less attempts, and lower complication rates compared to conventional DL. |
|  | Kim et al., 2019 | Full-text | Investigate whether sedative dose used during intubation affects post-intubation hypotension and identify other clinical factors that affects hypotension | Retrospective analysis | ED patients >19 years old requiring intubation |  | - Post-intubation hypotension rate | - Dose reduction of etomidate had a significant association with decreased post-intubation hypotension - Clinical condition of the patient prior to intubation (age, shock index, arterial acidosis) are risk factors for post-intubation hypotension - Non-depolarizing neuromuscular blocking agents had lower incidences of post-intubation hypotension compared to succinylcholine |
|  | Hwang & Jo, 2010 | Full-text | Evaluate factors associated with mortality for non-traumatic adult ED patients | Retrospective analysis | ED patients >15 years old requiring intubation |  | - Hospital mortality rate | - Over half of trauma patients survived to hospital discharge - Most common indication for intubation was failure of ventilation or oxygenation - Elevated respiratory rate and increased time between ED arrival and intubation were significantly associated with increased in-hospital mortality |
| Singapore General Hospital Emergency Airway Registry | Zakaria & Wong, 2017 | Abstract | Compare FPS rates amongst attending and non-attending EM physicians using DL and VL | Retrospective analysis |  | - Patient demographics - Indication for intubation - Device used - Method used - Difficult airway characteristic - Number of attempts - Attempt success or failure - Complications - Operator specialty and level of training - Rescue methods - Patient disposition | - FPS rate | - VL use was associated with higher FPS in difficult airways and with non-attending EM physicians - Attending EM physicians achieved higher FPS rates with DL |
|  | Weng et al., 2021 | Full-text | Determine whether VL or DL use affects FPS rates for endotracheal intubations | Retrospective analysis | All ED patients requiring intubation |  | - FPS rate - FPS rate for difficult intubations | - VL had a lower overall FPS rate compared to DL, though this is inconclusive for difficult airways |
|  | Wong & Ngo, 2009 | Abstract | Document the airway characteristics of patients with difficult airways | Retrospective analysis |  |  | - Number of attempts - Patient diagnoses - Rescue methods used - Adverse events rate | - Difficult airways are associated with higher rates of complications |
|  | Wong & Ng, 2008 | Full-text | Identify the reasons for difficult airways and the rescue methods used in EDs | Retrospective analysis |  |  | - Indication for intubation - Difficult airway characteristics - Number of attempts - Operator specialty - Medications used - Rescue device used - Complications | - Most difficult airways are managed successfully - Use of the bougie or other rescue devices like VL improves success rate |
| The Aberdeen Royal Infirmary Airway Registry | Yeap et al., 2019 | Abstract | Determine whether the addition of an airway adjunct improves FPS rate | Retrospective analysis | ED patients who underwent RSI | - Patient demographics - Indication for intubation - Difficult airway characteristic - Device used - Operator speciality - Ultimate success or failure - Adverse events | - FPS rate | - Use of bougie was not found to confer a significant benefit - Bougie use was associated with increased risk of complications |
| The Alfred Airway Registry | Groombridge et al., 2021 | Full-text | Evaluate the outcomes of implemented modifications to airway management practice and the impact of COVID-19 | Retrospective analysis | All ED patients requiring intubation | - Patient demographics - Indication for intubation - Glasgow coma score - Patient vitals - Operator specialty - Method used - Intubation maneuvers used - Medications used - Devices used - Confirmation of endotracheal tube placement - Patient disposition - Adjunct used - Number of attempts - Attempt success or failure - PPE used - Intubation team size and makeup | - Rate of hypoxia - FPS rate | - Intubations during the COVID-19 era were associated with more hypoxia despite implemented measures - FPS remained constant across time periods |
| The Royal North Shore Hospital Emergency Department Airway Registry | Vassiliadis et al., 2015 | Full-text | Compare direct and C-MAC VL in terms of FPS rate, airway grade, and complication rates | Retrospective analysis | ED patients who underwent intubation with a Macintosh or Miller intubation device | - Operator level of training - Number of attempts - Attempt success or failure - Devices used - Difficult airway characteristics - Medications used - Complications | - FPS rate - Airway grade - Adverse events rate | - VL use was associated with greater intubation success compared to DL, particularly in the setting of grade III/IV oral views |

**ANZEDAR** The Australian and New Zealand Emergency Department Airway Registry, **BCARE** British Columbia Airway Registry for Emergencies, **DREAM** Defense Registry for Emergency Airway Management, **EDIR** Emergency Department Intubation Registry, **JEAN** Japanese Emergency Airway Network Registry 1 and 2, **KEAMR** Korean Emergency Airway Management Registry, **NEAR** National Emergency Airway Registry, **NERAA** National Emergency Resuscitation Airway Audit

Direct laryngoscopy (DL), video laryngoscopy (VL), GlideScope video laryngoscope (GVL), emergency department (ED), emergency medicine (EM), first pass success (FPS), rapid sequence intubation (RSI)
