# Supplementary material for: Airway registries in primarily adult, emergent endotracheal intubation: a scoping review
Source: Scand J Trauma Resusc Emerg Med. 2023 Mar 8;31:11. doi: 10.1186/s13049-023-01075-z (PMC9993388; doi:10.1186/s13049-023-01075-z)
Supplement: Supplementary file 5 — Additional file 5. Intubation Practices and Adverse Events in Identified Airway Registries. [file 13049_2023_1075_MOESM5_ESM.docx]

Additional File 5: Intubation Practices and Adverse Events

| **Name of Registry** | **Author, Year of Publication** | **Most Common Intubator(s)** | **Most Common Indication for Intubation** | **FPS (%)** | **Most common device (%)** | **Rate of RSI and Most Common Medications (if available)** | **Total rate of adverse events (%)** | **Most common adverse event (%)** | **Definition of Intubation Attempt** | **Definition of FPS** | **Definition of hypoxia** | **Definition of hypotension** |
| --- | --- | --- | --- | --- | --- | --- | --- | --- | --- | --- | --- | --- |
| ANZEDAR | Freeman et al., 2021 |  | Medical  78.4% |  |  | 35.3% thiopentone (note - those intubated with etomidate were excluded from this study) |  |  |  |  |  | SBP <90 mmHg |
|  | Arnold et al., 2021 | Second attempts at intubation most often by EM residents (34.2%) and EM staff (36.8%) | Medical 73.2% | 84.3% | VL 54.3% |  |  |  | Single pass of the laryngoscope blade into the mouth | Successful placement of an endotracheal tube following the first pass of the laryngoscope into the mouth |  |  |
|  | Perera et al., 2021 | 85.7% EM physicians | Medical 72.8% | 85% |  |  |  |  | Each insertion of the laryngoscope blade into a patient’s mouth |  | Peripheral O2 saturation <93% measured by pulse oximeter |  |
|  | Ferguson et al., 2019 |  | Medical 76% | 84.4% (ketamine use), 84.6% (no ketamine use) |  | 5% ketamine in 2010, 36.8% ketamine in 2015 - other specific meds not reported |  |  |  |  |  |  |
|  | Powell et al., 2018 | EM physicians 92% | Medical  62% - most common overall was head injury 23% | 87% | VL 55% | 74% RSI; 52% Etomidate Succinylcholine 43% | 16% |  |  |  |  |  |
|  | Alkouri et al., 2017 | EM physicians 84.5% | Medical 76.4% - overdose/ ingestion 21.1% | 84.3% | VL 50.7% | 29.5% Propofol; 29.2% Thiopentone; 75% Succinylcholine | 26% | Hypoxia 11.9% | Single passage of the laryngoscope blade into the mouth |  | <93% SpO2 | Requiring IV fluids or vasopressor/inotrope treatment |
|  | Fogg et al., 2015 |  |  |  |  |  |  |  |  |  |  |  |
| BCARE | Botros et al., 2020 | 67.8% EM attendings |  | 78.8% (PGY1-2), 86.5% (PGY3-5), 85.7% (staff) – Overall = 85.2% | DL 24.2% in PGY1-2, 24.8% in PGY3-5, 30.1% by attendings – study only looked at DL |  | 16.9% | Hypoxia (% not reported) |  |  |  |  |
|  | JH Yoo, J Trojanowski, K Dullemond, C Liu, C Renschler, D Griesdale, J Brubacher, 2018 | Attending physicians more often in ED (48%) | ICH/stroke - 14.6%, seizure - 10.9%, sepsis 9.5% | 81.8% in ED, 79.2% in ICU, 77.5% on ward | VL 57.7% |  | 14.1% |  |  |  |  |  |
|  | C Liu, JH Yoo, J Trojanowski, M Laberge, D Griesdale, JR Brubacher, 2018 |  |  |  |  |  |  |  |  |  |  |  |
| Chulalongkorn Airway Registry | Saoraya et al., 2021 | 53.6% EM physician mid-level trainee (1st or 2nd year resident) | Medical 35.5% - CHF 18.6% | 74.30% | DL 84.5% | 49.1% Etomidate; 55.5% Succinyl- choline; 57.4% RSI rate; 35.5% Sedation without paralysis; 7.1% No meds | 13.2% | Hypotension 4.1% | Attempt of laryngoscopy | Successful intubation during the first attempt |  |  |
| Cipto Mangunkusumo General Hospital Airway Registry | Sulistio et al., 2021 | 63.8% Anaesthetists | Medical 93% - Respiratory failure 55.8% | 89.6% overall. 88.9% for anesthesia; 55.4% for EM, 58.5% for other |  | 68.4% Fentanyl; 41.6% Rocuronium; 21.6% No medication; 9% RSI rate | 22.1% | Hypotension 4.3% |  |  | SpO2 <93% | Requiring fluid |
| Cleveland Clinic Emergency Airway Registry | Good et al., 2017 | EM physicians, EMS (% not reported) |  |  | DL 60.6% |  |  |  |  |  |  |  |
|  | Phelan et al., 2016 |  |  |  |  |  |  |  |  |  |  |  |
|  | Phelan et al., 2011 |  |  |  |  |  |  |  |  |  |  |  |
|  | Phelan et al., 2010 | 91% EM residents (91%); other residents < EM attendings |  | 74% |  | 73% RSI rate |  |  | Single insertion of a laryngoscope for oral intubations or a single insertion of an endotracheal tube for nasal attempts |  |  |  |
| Continuous Quality Improvement Database | Pacheco et al., 2021 | 56% EM physician PGY1 or 2 | 66% medical - 68% airway protection | For FPS without adverse events. 92.4% (non-difficult airway), 82.1% (anatomic), 81.7% (physiologic), 70.9% (both) | 94% VL, 6% DL | 100% RSI rate- only included if underwent RSI. 60% Succinyl- choline; 88% etomidate | 5.7% (non difficult airway), 12.6% (anatomic), 14.5% (physiologic), 24.4% (both) | 9.2% hypoxia (in anatomically difficult airways); 8.6% hypoxia (in physio- logically difficult airways) |  |  | SpO2 <93% |  |
|  | Sakles et al., 2019 | 52% EM PGY 1 or 2 |  | 92.4% | VL 97.8%; DL 2% | 100% RSI rate - only included if underwent RSI. 81.6% Etomidate; 54% Succinylcholine | 14.6% | Hypoxia 11.2% |  |  | SpO2 <90% | SBP <90mmHg |
|  | Sakles et al., 2017 | EM residents only - not included in study otherwise | Medical 57%, Airway protection 78.5% | GlideScope: 81.4% (soiled airway), 91.0% (clean)  DL: 65.5% (soiled), 75.8% (clean) | DL 52% | 100% RSI rate 100% - only included if RSI done. 51.5% Succinyl- choline; 92% Etomidate |  |  | Insertion of the laryngoscope blade into the mouth of the patient, regardless of whether an attempt was made to insert a tracheal tube | Successful tracheal intubation on a single laryngoscope insertion |  |  |
|  | JC Sakles, JM Mosier, AE Patanwala, B Arcaris, JM Dicken, 2016 | 63.8% were EM PGY1 or 2 | Medical  71.7% | 87.6% (C-MAC), 75.3% (Mac) | Only investigating device use as DL | 100% RSI rate - only included if received RSI. 91.2% Etomidate; 50% Succinylcholine |  |  | Insertion of the laryngoscope blade into the mouth of the patient, regardless of whether an attempt was made to insert a tracheal tube | Successful tracheal intubation on a single laryngoscope insertion | SpO2 <90% |  |
|  | JC Sakles, JM Mosier, AE Patanwala, JM Dicken, 2016 | EM residents only - not included in study otherwise | Trauma 77%; 92% Airway protection | 93% (apneic oxygen), 84% (no apneic oxygen) | VL 80% | 100% RSI rate - only included if RSI performed. 60% Succinylcholine |  |  | Insertion of the laryngoscope blade into a patient’s mouth as an intubation attempt, regardless of the outcome of the attempt |  | SpO2 <90% during intubation |  |
|  | JC Sakles, JM Mosier, AE Patanwala, B Arcaris, JM Dickens, 2016 | EM residents only - not included in study otherwise | Medical 67%; 76% Airway protection | **FPS without hypoxemia. 82.1% (apneic oxygen), 69.0% (no apneic oxygen) | 82% VL | 100% RSI rate - only included if RSI performed. 91% Etomidate; 52.5% Succinylcholine |  |  | Insertion of the laryngoscope blade into the mouth of the patient, regardless whether an attempt was made to insert a tracheal tube | Successful tracheal intubation on a single laryngoscope insertion. | SpO2 <90% |  |
|  | Dicken et al., 2016 | EM residents - only patients intubated by residents were included in analysis |  | 93.4% (C-MAC as VL), 92.3% (GVL- MAC as VL), 64.9% (C-MAC as DL), 43.2% (GVL- MAC as DL) | 100% VL - only included patients undergoing intubation with VL in the study |  |  |  |  | Successful tracheal intubation on a single insertion of the laryngoscope blade |  |  |
|  | JC Sakles, PP Javedani, et al., 2015 | EM residents only - not included in study otherwise | Medical 56%; 61.5% Airway protection |  | VL 55.2%; DL 44.7% | 85.2% RSI rate; 43% Rocuronium; 76% Etomidate | 49.5% (patients with esophageal intubation), 19.8% (no esophageal intubation) | Hypoxemia 16.8% - 35.5% | Insertion of the laryngoscope blade into the oropharynx, regardless of whether an attempt was made to pass the tube | Correct placement of the tube in the trachea as confirmed by a combination of standard clinical assessments and end-tidal carbon dioxide capnometry on first attempt |  |  |
|  | JC Sakles, JM Mosier, et al., 2015 | 100% EM residents, EM attendings, EM-paediatrics residents, 58% PGY 1 or 2 EM residents | Medical 58%; 61% airway protection |  | VL 35%; DL 23%; 40% other. reported for rescue attempts only | 79% rate of RSI; 39.6% both rocuronium and succinyl- choline, no induction agent reported |  |  | Insertion of the laryngoscope blade into the patient’s mouth, regardless of whether an attempt was made to pass a tracheal tube | Appropriate placement of a tracheal tube into the patient’s airway as confirmed by standard clinical means, including end-tidal CO2 capnometry on first attempt |  |  |
|  | Arcaris et al., 2015 | 100% EM residents - only included intubations performed by EM residents in study |  | 87.7% (Mac VL), 74.0% (Mac DL) | DL 51%; VL 49% | 100% RSI rate - only included patients undergoing RSI |  |  |  | Success with a single laryngoscope insertion |  |  |
|  | Corn et al., 2015 |  |  | 77.9% (GVL), 62.6% (DL) | DL 54%; VL 46% | 100% RSI rate - only included patients undergoing RSI |  |  |  | Successful intubation with a single laryngoscope insertion |  |  |
|  | Patanwala et al., 2014 | 95.2% EM residents PGY1-3. Overall : 96% by EM physicians | Medical 56%; 70% airway protection | 77% (etomidate) 79.1% (ketamine) | VL 49% | 100% RSI rate - only included patients undergoing RSI. 50.7% Rocuronium |  |  | Insertion and subsequent removal of the laryngo- scopic device from the patient’s mouth, regardless of whether an attempt was made to pass a tracheal tube | Correct placement of the tracheal tube into the trachea, which was confirmed by end-tidal CO2 capnometry, pulse oximetry, chest auscultation, observation of chest excursion, absence of epigastric sounds, and misting of the endotracheal tube on the first attempt |  |  |
|  | JC Sakles, J Mosier, AE Patanwala, J Dicken, 2014 | Only intubations by residents PGY1-3 included in study | Medical 57.8%- 81.7% | For DL: PGY1 (69.9%), PGY2 (71.7%), PGY3 (72.9%)  For GVL: PGY1 (74.4%), PGY2 (83.6%), PGY3 (90.0%) | DL 64% | 83.3% - 89.1% Rate of RSI; 40.2%-43.2% Rocuronium; 39.6%-48.9% Succinyl- choline; 79% - 84.7% Etomidate |  |  |  |  |  |  |
|  | JC Sakles, AE Patanwala, J Mosier, J Dicken, N Holman, 2014 | EM physicians: 96.6%, 60.8% EM PGY1 or 2 | 62.1%Trauma, 65% Airway protection | 81.0% (GVL), 58.2% (cGVL) | 100% VL - only included if VL used | 85.6% RSI rate |  |  | Insertion of the video laryngoscope blade into the mouth of the patient, regardless whether an attempt was made to insert a tracheal tube | Tracheal intubation on a single laryngo- scopic insertion |  |  |
|  | JC Sakles, AE Patanwala, JM Mosier, JM Dicken, 2014 | EM physicians 100% - not included otherwise. 61% PGY 1 or 2 | Medical 56%; 61% Airway protection | VL: 90.8% (no difficult airway characteristics), 85.1% (1 DAC), 80.5% (2 DACs), 68.9% (>=3 DACs). DL: 82.0% (no DACs), 69.4% (1 DACs), 65.8% (2 DACs), 54.1% (>=3 DACs) | VL 57%, DL 43% | 86% RSI rate |  |  | Insertion of the laryngoscope blade into the mouth of the patient, regardless whether an attempt was made to insert a tracheal tube | Correct placement of the tracheal tube in the airway on a single laryngos- copic insertion. |  |  |
|  | Mosier et al., 2013 | EM residents, EM attendings: 65% by EM PGY1 or 2 | Trauma 50.7%; 61% Airway protection | 82% (GlideScope), 84% (C-MAC) | 100% VL - only included if VL used | 83% RSI; 72% Etomidate; 44% Succinylcholine |  |  | Insertion of the laryngoscope blade into the oropharynx regardless of whether an attempt was made to pass the endotracheal tube | Correct placement of the endotracheal tube in the trachea, as confirmed by end-tidal CO2 capnometry, pulse oximetry, chest auscultation, observation of chest excursion, absence of epigastric sounds, and misting of the endotracheal tube on the initial attempt |  |  |
|  | JC Sakles, CS chiu, J Mosier, C Walker, U Stolz, 2013 | EM attendings, EM residents, other residents. EM physicians: 97%, 56.5% EM PGY 1 or 2 | Medical 53% - 62.5% Airway protection | 72.9% | DL 54.5% | 85% RSI rate; 75% Etomidate; 43.9% Rocuronium | Total: 14.2% (first attempt), two attempts 47.2%, three attempts 63.6%, four attempts 70.6% | 9.2% Oxygen desaturation in first attempt; 37.8% in multiple attempts group |  | Correct placement of the endotracheal tube in the trachea as confirmed by end-tidal CO2 capnometry, pulse oximetry, chest auscultation, observation of chest excursion, absence of epigastric sounds, and misting of the endotracheal tube on first attempt | A decrease in oxygen saturation greater than or equal to 10% |  |
|  | JC Sakles, J mosier, M Cosentino, A Patanwala, 2013 | EM attendings, EM residents (% not specified) | Trauma 100% - only included if intubated for traumatic indication |  |  | 100% RSI rate - only included if RSI performed |  |  |  |  | Desaturation of 10% or more |  |
|  | J Sakles, D Falvey, N Stea, U Stolz, 2013 | Attending EM physician, EM residents in years 1-3, medical students: % not reported |  |  |  |  |  |  |  |  |  |  |
|  | JC Sakles, J Moiser, S Chiu, M Cosentino, L Kalin, 2012 | 96% by EM physicians - 95% by EM trainees PGY1-3 | Medical 59% - 59% Airway protection | 79.2% (C-MAC), 73.1% (DL) | DL 66% | 84.5% RSI rate |  |  | Insertion of the device into the mouth regardless of whether there was an attempt to pass the tube | Correct placement of the endotracheal tube in the trachea, as confirmed by end-tidal CO2 capnometry, pulse oximetry, chest auscultation, observation of chest excursion, absence of epigastric sounds, and misting of the endotracheal tube on initial attempt |  |  |
|  | Sakles and Kalin, 2012 | EM residents, EM attendings: 57.7% EM PGY1 or 2 | Trauma 68% | 82.9% (Gliderite rigid stylet), 67.% (standard malleable stylet) | VL 100% - only VL intubations included |  |  | Hypoxia 22% |  |  | Drop in oxygen saturation below 90% during the procedure or a drop in saturation of more than 10% if the starting saturation was less than 90%. |  |
|  | Sakles et al., 2012 | 97.6% by EM physicians: 54.3% by PGY 1 or 2 | Trauma 53% | 69% (DL), 75% (VL) | DL 62%, VL 38% | 87% RSI rate | 16.7% | Hypoxia 10% | Insertion of the laryngoscope blade into the patient’s mouth, regardless of whether an attempt to pass a tracheal tube took place | Placement of an endotracheal tube on the first attempt. |  |  |
|  | Patanwala et al., 2011 | 53.8% by EM PGY1 or 2. | Trauma 54.4%- 72.5% Airway protection | 72.6% (succinyl- choline), 72.9% (roc- uronium) | DL 59% | 100% RSI rate - only included if underwent RSI; 100% Etomidate - only included if received etomidate; 65%. Rocuronium; 35% Succinylcholine |  |  | Introduction of the laryngoscope into the patient’s mouth and its removal regardless of whether an endotracheal tube was inserted | Proper placement of the endotracheal tube without requirement for a surgical airway on the first attempt |  |  |
| DREAM | Mendez et al., 2021 | EM physicians only - 61% PGY2, 28% PGY 1 | Trauma 64% | 93% | VL 86% |  |  |  |  |  |  |  |
| EDIR | Hale et al., 2017 | 75.3% ED physician - 65% adolescent intubations;, 71% young adult intubations; 76% older adult intubations | Medical 75%, 21% Cardiac arrest | 94% (adolescents), 90% (young adults), 85% (older adults). 85.5% overall |  | 68% RSI rate; 43% Thiopental; 65% Succinylcholine | 9.8% | Hypotension 4.2% |  |  | SpO2 <90% | SBP<90 mmHg |
|  | Kerslake et al., 2015 | EM (78%), anesthesia (22%) | Medical 57% | 85% |  | 74% RSI rate; 17% No medications; 65%  Thiopental; 95% Succinylcholine | 8% | Hypotension 4.5% |  |  | Oxygen saturation <90%, but only if oxygen saturation was >90% prior to intubation | SBP <90mmHg, but only if SBP was >90mmHg prior to intubation |
|  | Paul et al., 2012 | 93.8% EM - training level not specified | Definite airway compromise due to decreased conscious level 62.5% | 80% |  | Excluded if RSI performed |  |  |  |  |  |  |
|  | Reid et al., 2011 | 5 surgical airways performed in total - Three by EM trainees, one by EM specialist, one by ENT specialist. |  |  |  | 66.2% RSI rate |  |  |  |  | O2 sat <90% | systolic blood pressure less than 90 mmHg |
|  | Donald, 2011 | Emergency physicians 88%, 63% of these were ED residents. | Trauma 41% | 87% |  | 100% RSI rate - not included otherwise; 59% Thiopentane | 11% | Hypotension 4% |  |  |  | Requiring fluids |
|  | Stevenson et al., 2007 | Anesthesia 56% | 62% Medical | 82% (EM), 91% (anesthesia) |  | 85% RSI rate;, 44% Etomidate |  | Hypotension 6% |  |  | SpO2 <90% | Requiring treatment |
|  | Graham et al., 2003 | EM 51%, Anesthesia 48% | Medical = 57% | 83.8% (EM), 91.8% (anesthesia) |  | 100% RSI rate - not included otherwise | 10.8% | Hypotension 3.4% |  |  |  | SBP <90mmHg |
| JEANI+II | Yamanaka et al., 2020 | 82.3% by senior ED resident or ED attending physician | 58.6% "internal disturbance of consciousness" | 65.2% | DL 91.2% | RSI rate not stated. 63.5% Medication used. Specific meds not reported | 17.1% | Esophageal intubation 4.4% | Single insertion of the laryngoscope past the teeth | Tracheal tube being placed through the vocal cords, with confirmation by quantitative or colorimetric end-tidal carbon dioxide monitoring, on first attempt |  |  |
|  | Goto et al., 2017 | EM attendings or residents 57%; transitional year resident (PGY1 or 2) 37% | Medical 54% | 74% | DL 58%, VL 40% | 53% RSI rate; 49% Midazolam; 84% Rocuronium | 14% |  | Oral attempt was defined as a single insertion of a laryngoscope (or other device) past the teeth | Endotracheal tube being placed past the vocal cords, with confirmation by quantitative or colorimetric end-tidal carbon dioxide monitoring, on first attempt | Pulse oximetry saturation <90% | SBP <90mmHg |
|  | Okubo et al., 2017 | EM resident 35% | 81.2% Medical - 40% altered mental status | 73% (RSI), 63% (non-RSI) | DL 97% | 32% RSI rate; 29% Midazolam (of all patients); 70% Rocuronium (of patients who underwent RSI only) | Total: 12% (RSI), 13% (non-RSI) | Esophageal intubation: 4% RSI; 5% non-RSI | Single insertion of the laryngoscopy past the teeth | Proper placement of an endotracheal tube through the vocal cord confirmed by quantitative or colorimetric end-tidal CO2 monitoring, on first attempt | Pulse oximetry saturation less than 90% during an intubation attempt, not secondary to esopha- geal intubation | SBP <90mmHg |
|  | Nakao et al., 2015 | EM physicians and residents 60% | Trauma 100% - Only trauma patients included; 32.6% traumatic cardiac arrest | 63.8% | DL 90.5% VL 4.1% | 23.9% RSI rate of all trauma patients, 35.5% of patients without cardiac arrest - specific meds not reported | 10.8% | Esophageal intubation 3.5% | Single insertion of the laryngoscope (or other device) past the teeth. | Successful if it resulted in the tracheal tube being passed through the vocal cords on first attempt | Pulse oximetric saturation less than 90% during an intubation attempt | Systolic blood pressure less than 90 mm Hg |
|  | Goto et al., 2015 | Transitional year resident PGY 1 or 2 40%, specialty not specified | Medical 50% | 68.0% | DL 96% | 19% RSI rate |  |  | Single insertion of the laryngoscope (or other devices) past the teeth | Resulted in a tracheal tube being placed through the vocal cords on first attempt |  |  |
|  | Goto et al., 2014 | Transitional year residents (PGY1 and 2) EM residents (PGY 3,4,5) only - not included in study otherwise | Medical 84% | 64% (medical indication of intubation), 55% (trauma indication) |  | 17% RSI rate | 13% |  | Single insertion of the laryngoscope (or other device) past the teeth | Endotracheal tube being placed through the vocal cords on first attempt |  |  |
|  | Imamura et al., 2013 | EM resident 29%, EM staff physician 19%, transitional year resident (PGY 1 or 2) = 41% | 83% medical - 31% cardiac arrest | 71% (geriatric), 64% (younger) | DL 96% , VL 2% | 18% RSI rate, 78% no paralytic, 62% no sedative, 21% benzodiazepine; 14% Rocuronium | 11% | Esophageal intubation with delayed recognition 4.2% | Single insertion of the laryngoscope (or other device) past the teeth. | Successful if it resulted in an endotracheal tube being placed through the vocal cords | SpO2 < 90% | SBP < 90mmHg |
| KEAMR | Kim et al., 2017 | EM physicians - % not reported | Medical 70.7% - "antici- pated oxygen- ation or airway protection failure" 40.3% | 84.98% (simulation based emergency airway management program), 84.50% (control). Overall = 84.8% |  | 61.3% RSI rate |  |  |  |  | SpO2 <90% |  |
|  | Lee et al., 2016 | PGY 1 EM resident 41% | Medical 72% - 51% failure to protect airway | Season 1: 89.6% (GVL), 86.1% (DL). Season 2: 94.8% (GVL), 76.2% (DL) | VL 55.4%, DL 45% | 46% RSI rate |  |  | Single insertion of the laryngoscope past the teeth. |  |  |  |
|  | Choi et al., 2015 | EM physicians: 85.7%, most common junior residents (PGY1-3) = 75.9% | Medical 74.8% | 85.7% (GVL), 82.3% (MAC) | GVL 13.4%, MAC 27% | 35.7% RSI rate |  |  | Single insertion of the laryngoscope past the teeth |  |  |  |
|  | Cho et al., 2015 | EM physicians = 94.5%, most common PGY1 = 41% | Trauma - only trauma indications included. 36.9% Brain injury | 80.6% | DL 76.2%, VL 18.6% | 44.7% RSI rate | Total: 6.5% | Esophageal intubation 3.4% | Single insertion of the device past the teeth | Proper placement of the endotracheal tube through the vocal cords on first attempt |  |  |
|  | Cho et al., 2013 | EM Physicians, EM Residents, or residents from other departments - % not reported | Medical 87.9% | 79.9% (<65 years), 79.7% (65-79), 82.7% (80+) |  | RSI rate 27.8% (<65); 26.5% (aged 65-79), 25.2% (patients aged 80+) |  | Esophageal intubation 4.3% | Moment of laryngoscope insertion into the mouth to the moment of elimination and the completion of endotracheal intubation on the first attempt |  |  |  |
|  | Kim et al., 2013 | EM physicians, EM residents, residents from other specialities (% not reported) | Medical 76% | 81.0% | DL 85% VL 14% | 41% RSI rate |  |  | An attempt was defined as a single insertion of the device past the teeth |  |  |  |
|  | Kim et al., 2011 |  |  | 86.1% (GVL), 80.9% (MAC) |  |  | Total: 7.2% (GVL), 14.9% (MAC) |  | An attempt was defined as the introduction of the laryngoscope into the mouth |  |  |  |
| King Abdulaziz University Hospital Airway Registry | Bakhsh et al., 2021 | EM trainee 71.9% | Airway protection 48.6% | 80% (pre-intervention was 57.1%) | VL 69.2% |  |  | Esophageal intubation 3.4% | Insertion of the laryngoscope blade into the oropharynx regardless of whether an attempt was made to pass the endotracheal tube | Successful tracheal intubation on a single laryngoscope insertion |  |  |
| Middlemore Hospital Airway Registry | Brainard et al., 2014 |  | Medical 52% - 12.4% ICH/stroke |  |  |  |  |  |  |  | O2 saturation <90% | Requiring treatment with fluid/ vaso- pressors |
| NEARI | Sagarin et al., 2003 | EM residents, EM attendings, non-EM residents - % not reported |  |  |  | 79% RSI rate |  |  |  |  |  |  |
| NEARII | Walls et al., 2011 | EM physician 87% | Medical 67% - Cardiac arrest 12% | 81% |  | 69% RSI rate | 12% | Esophageal intubation - 2.9% | Single effort to place an airway |  |  |  |
|  | Sagarin et al., 2005 | EM residents 78% in USA and Canada | Medical 68% - cardiac arrest 12% | 83% (residents), 89% (attendings) |  | 78% RSI rate |  |  | Single insertion of laryngoscope for oral attempts or a single insertion of an endotracheal tube for nasal attempts | Endotracheal tube placed through the vocal cords on first attempt |  |  |
|  | Deiorio, 2005 |  |  |  |  |  |  |  |  |  |  |  |
|  | Collins et al., 2005 | EM physicians 92.2%, most common EM PGY-2 31.6% |  |  | DL = 86% | 86% RSI rate; 32.8% Etomidate; 75% Succinylcholine | 9.8% | Aspiration and hypotension- 2% each |  |  |  |  |
|  | Bair et al., 2002 | 79% of rescue RSIs by EM physicians, 53% of rescue surgical airways by EM physicians | Rescue intubations: Medical 73% |  |  | 49% RSI rate in rescue attempts |  |  | Single pass of laryngoscope blade into mouth |  |  |  |
|  | Walls et al., 1999 | EM physicians 91% of adults and 79% of paediatrics intubations |  | 95% (RSI), 90% (SED), 89% (NOM), 83% (NTI) |  | 71% RSI rate | 12.9% | "Technical problem" 5.6%; Immediate compli- cation 3.5% |  |  |  |  |
| NEARIII | Nikolla et al., 2022 | EM PGY2 most common = 42% | Altered mental status 26.4% | 91.9% | VL 80.3% | 100% RSI rate - excluded if RSI not performed; 60% Rocuronium | 16.5% | Hypoxia 10% |  | Successful intubation with a single laryngoscope blade insertion | SpO2 <90% during or immedi- ately after attempter desat of >10% of absolute | SBP < 100mmHg |
|  | Trent et al., 2021 | EM or paediatric EM physicians 94%; most common was EM PGY-3 46% | Trauma 23% - only trauma indications included - head injury 33% | 86.80% | VL 71%, DL 28% | 83% RSI rate | 12% | Hypoxia 7% | Any insertion of a laryngoscope beyond the alveolar ridge, whether or not an endotracheal tube was inserted | Properly placed endotracheal tube during an attempt, which was confirmed by colorimetric or quantitative end-tidal CO2 on first attempt | SpO2 < 90% or >10% drop if <90% to start | SBP < 100mmHg |
|  | Nikolla et al., 2021 | EM PGY3-4 46.6%; EM Resident PGY1-4 90.6% | 100% Medical - only medical indications included. altered mental status 27.2% | DL: 89.7% (supine), 87.1% (ramped); VL: 90.9% (supine), 91.6% (ramped) | DL 52.6% VL 47.4% | 55.1% Rocuronium | Total: DL - 9.8% (supine), 12.4% (Ramped), VL - 13.8% (supine), 17.8% (ramped). Total overall = 12.1% | Hypoxia 8.4% |  |  | SpO2 <90% or a drop of >10% |  |
|  | Driver et al., 2021 | EM PGY3-4 48% | Medical 74.8%, | 61% ketamine only; 85% topical anesthesia; 90% RSI | VL 69.3% | 98.5% RSI rate | 9.8% | Hypoxia 8.1% |  |  | SpO2 <90% |  |
|  | Sandefur et al., 2021 | EM physicians 94% overall with EM PGY3 and above as most common intubator at 58% | Only angioedema included | 81% | Most common device: flexible endoscope (49%), 42% of attempts were nasal route. 42% VL | 61% RSI rate | 17% | Hypotension 13% |  |  |  |  |
|  | Kaisler et al., 2021 | 91% of first attempts by EM physician, PGY-3 47.5% | Angioedema 32% and non-angioedema airway obstruction 31% most common reasons for awake intubation attempt | 85% | Flexible endoscope 78% (rescue attempts both VL and DL 17% each) | 41% Ketamine | 16% | Hypoxia 12% |  | Tube placement being confirmed by qualitative or quantitative EtCO2 monitoring on first attempt |  |  |
|  | Levin et al., 2021 | 39.2% EM PGY4; 87.8% EM residents overall | Medical 80.2% | 88.7% | VL 53.4%, DL 45.7% | 100% RSI rate - only RSI included in analysis, 42.1% Rocuronium 78.9% Etomidate | 14.0% |  | Single effort to place an endotracheal tube in which the leading edge of the laryngoscope blade entered the oral cavity past the alveolar ridge |  | SpO2 <90% | SBP < 100mmHg |
|  | Chan et al., 2021 | >50% by senior physician (PGY5 or higher, clinical fellows, or attending physicians) | Medical 84.8% - cardiac arrest 31.1% | 86.5% | VL 75.6% | 67.3% RSI rate;, 70.6% Etomidate; 92.2% Succinylcholine | 13.8% | Hypoxia 11.2% |  |  |  |  |
|  | April et al., 2021 |  | Medical 72.5-73.9% | 88.4% (non-cardiac arrest patients), 87.9% (cardiac arrest) | VL 53.2%-54.1%  DL 44.7% - 45.2% | 66.2-77% Etomidate; 49.2-56.1% Rocuronium | 12.2% (non-cardiac arrest patients), 55.% (cardiac arrest patients) | Hypoxia 7.5-19.1% |  |  | SpO2 90% or lower | SBP <90mmHg |
|  | Kunzler et al., 2021 |  | 11% for overdose, 89% for other indications (not specified) | 90.5% (overdose patients), 87.5% (non-overdose indications) |  |  |  |  |  |  |  |  |
|  | Garcia et al., 2021 | "PGY3+" = 54% |  |  | DL 68.4% |  |  |  |  |  |  |  |
|  | Godwin et al., 2020 | EM PGY 3 40.2% | Only patients intubated for asthma included in the study | 90.8% | VL 66.9% | 96.5% RSI rate; 50.9% ketamine; 55.1% Rocuronium | 12.1% | Hypoxia 8.1% |  |  |  |  |
|  | Driver et al., 2020 | 54% PGY3-4 in the standard geometry group. 48% PGY1-2 in hyperangulated group | Medical - 79% in standard geometry group, 67% in hyperangulated group | 91.9% (standard geometry), 89.2% (hyperangulated) | 100% VL - 61% standard geometry, 39% hyperangulated blade | Neuromuscular blocking agent used in 84% of hyperangulated group, 88% of standard geometry group. Most common was 45% Rocuronium hyperangulated group, 46% in standard geometry group | Standard geometry 8%; hyperangulated 9% |  | Single insertion of the laryngoscope blade into the mouth |  | SpO2 <90% |  |
|  | Mohr et al., 2020 | PGY1-4 EM 90.4%,  EM PGY3 39.3%, residents |  | 86.5% (total), 87.4% (sepsis), 86.9% (non-sepsis) |  | 89% RSI rate; 71% Etomidate | Total: 12.1% (overall), 23.1% (sepsis), 12% (non-sepsis) |  |  |  | SpO2 <90% | SBP < 100mmHg |
|  | April et al., 2020 |  | Medical 66.8% in ketamine group, 75% in etomidate group | 90.0% (ketamine), 90.1% (etomidate) | VL ketamine 59.1%, etomidate 51%. | 89.5% Etomidate; 63% Rocuronium | Total: 18.7% (ketamine), 11.2% (etomidate) | Hypoxia 10.6% (ketamine), 6.9% (etomidate); |  |  | Oxygen saturation drop of >10% or below 90% | SBP < 100mmHg |
|  | Lembersky et al., 2020 |  | Medical 75.7% - 25.2% Non-overdose mental status change in sedation group, 56.7% cardiac arrest in no sedation group |  |  | 89% RSI rate; 42.6% Fentanyl; 50.3% Succinylcholine |  | Hypotension - 14.9% sedated patients, 39.3% non-sedated |  |  |  | SBP < 100mmHg |
|  | Brown et al., 2020 | PGY 3 most common - 37.1% in DL cohort, 37.4% in VL cohort | Medical 69.3% | 90.9% (unassisted VL), 81.1% (augmented DL) | VL 43.3%, DL 56.7% | 80.8% RSI rate | 11.1% (VL) , 12% (DL) |  | Any insertion of a laryngoscope beyond the teeth whether or not an endotracheal tube (ETT) was passed | Properly placed tracheal tube during the first laryngoscopic attempt, confirmed by either colorimetric or quantitative end-tidal CO2 on first attempt |  |  |
|  | Runde et al., 2020 | EM PGY 1 only included in study |  |  |  |  |  |  |  |  |  |  |
|  | Watase et al., 2020 |  |  | Hyperangulated: 94.5% (CL grade 1), 83.9% (grade 2), 54.1% (grade 3), 18.8% (grade 4). DL: 95.3% (grade 1), 84.4% (grade 2), 40.2% (grade 3), 16.1% (grade 4) | VL 44.7%, DL 55.3% |  |  |  |  |  |  |  |
|  | Joshi et al., 2020 |  |  |  |  | 83.1% Propofol; 64.1% Etomidate; 52.7% Fentanyl |  |  |  |  |  |  |
|  | Stoecklein et al., 2019 | 41.1% EM PGY 3; 93.1% by EM residents PGY1-4 | Medical 75.8% | 87.0% | VL 65.8%, DL 34.2% | 100% RSI rate - only included if RSI indicated | Total: 18.3% (non supine), 11.9% (supine) | Hypoxia 7.4% |  |  | SpO2 <90% or a decrease of >10% | SBP < 100mmHg |
|  | Monette et al., 2019 | EM trainee 92% , PGY3+ = 48% |  | 71% (PGY1), 82% (PGY2), 89% (PGY3+), 84% (all residents), 88% (attending) | DL 88% |  |  |  |  |  |  |  |
|  | Roy et al., 2019 |  |  | 85% | VL > DL - % not reported |  | 13% | Hypoxia 7-8% |  |  |  |  |
|  | Brown et al., 2019 |  | Medical  76.3% | 91% (ultimate success after more than one attempt was 94%) | All intubations were cricothyrotomies by open surgical technique and 46% aided by bougie |  |  |  |  |  |  |  |
|  | Runde et al., 2019 |  |  |  | VL 68.33% DL 31.67% |  | VL 12.9%; DL 12.36% |  |  |  |  |  |
|  | April et al., 2018 | EM PGY3-4 47.7% - 48.6% of intubations | Medical 68.6%-78.7% | 87% (succinylcholine), 87.5% (rocuronium) | VL 58.9% (rocuronium group), 65.8% (succinylcholine group) | 79-84.7% Etomidate; 55.8% Succinylcholine | 14.7% (succinylcholine group), 14.8% (rocuronium group) | Hypoxia 8.6% (rocuronium group), 8.8% (succinylcholine group) | Any single effort to place an endotracheal tube in which the leading edge of the laryngoscope blade entered the oral cavity past the alveolar ridge |  | SpO2 <90% | SBP < 100mmHg |
|  | Maddry et al., 2018 | PGY-2 EM residents 62% | Trauma 68.6% | 84.7% | VL 57.3% | Rate of RSI not reported, 58.3% Ketamine; 64.8% Rocuronium | 24.3% | Hypoxia 14.1% |  |  | SpO2 <90% | SBP < 100mmHg |
|  | Hayden et al., 2018 | EM PGY3-4 37.8% | For flexible fiberoptic intubations (FFI): Medical 81.1% - airway obstruction 36.1% | 51.1% | Fiberoptic endoscope intubations only | 11.1% RSI rate in patients needing flexible fiberoptic intubations | 20.8% | Esophageal intubation with immediate recognition 2.9% | Any single effort to pass the tracheal tube, which occurred when the leading edge of the airway device (laryngoscope, fiberscope, etc.) entered the oral cavity past the alveolar ridge |  |  | Required IV fluid |
|  | Goldberg et al., 2018 |  |  |  | Bougie 16%, hyperangulated laryngo- scope 7.1%, traditionally shaped blade 91.5% |  |  |  |  |  |  |  |
|  | Kilgo et al., 2018 | 95% resident physicians, 5% attending physicians | Medical  66% | 84% |  | 83% Etomidate 66% Rocuronium | 11% |  |  |  |  |  |
|  | Ruderman et al., 2018 |  |  | 81.0% (DL), 90.0% (VL) | "VL was used more than twice as often as DL" - no % reported |  |  |  | Insertion of the device into the mouth past the alveolar ridge regardless of success |  |  |  |
|  | Van Devern et al., 2017 | Anesthesia provider 28.6% | Medical 44.7% | 71% | VL 54%, DL 46% | 66% RSI rate - specific meds not reported | 24% | Hypoxemia 17% | Insertion of the laryngoscope past the teeth | Successful placement of an endotracheal tube in the airway confirmed by colorimetric or quantitative end-tidal CO2 on first attempt |  |  |
|  | April et al., 2017 | EM residents 95%, 64% of these PGY-2 (most common) | Trauma 71% - polytrauma 22.3% | 83% | VL 55.2%, DL 44.4% | 81% RSI rate; 59.8% Ketamine; 62.9% Rocuronium | 23.6% | Hypoxia 13.8% |  |  | Oxygen  saturation less than 90% |  |
|  | Carlson et al., 2015 | 96.9% EM physicians, PGY 2 and 3 were most common at 34.8% each | "GI bleed" indications only | 88.5% (DL), 93.3% (VL) | 90.8% DL, 9.2% VL | 93.9% RSI rate |  |  | Any single effort to place a tracheal tube, which was defined by the leading edge of the laryngoscope blade passing the alveolar ridge |  |  |  |
|  | Brown et al., 2015 | 95% EM physicians and of those 79% trainees (41% PGY3 or 4) | Medical 65% - Altered mental status, not overdose 10% | 83% | DL 84%, VL increased from 1% to 27% over study period | 85% RSI rate; 91% Etomidate; 75% Succinylcholine | 12% | Esophageal intubation 3.3% | Any single effort to place a tracheal tube, which occurred when the leading edge of the laryngoscope blade entered the oral cavity past the alveolar ridge |  |  | Required IV fluid |
|  | Brown et al., 2007 |  | Medical 69% - Seizure 9.9% | 96% | VL only |  | 9.9% | Esophageal intubation 6.2% |  |  |  |  |
| NERAA | Umana et al., 2022 | 54% EM physician - 46% anesthesia/ intensive care medicine. 90% of intubating physicians were residents | Medical 83% - Cardiac arrest 30% | 89% | VL 53% | 50% Propofol; 74% Rocuronium; 23% No Sedation; 20%  No paralytic | 19% | Hypotension 10% | Single passage of laryngoscope into the mouth | Successful passage of endotracheal tube through vocal cords on first attempt | SpO2 <93% | Requiring fluid or inotropes |
| Samsung Medical Centre Emergency Airway Program | Kim et al., 2019 |  | Respiratory failure 50.1% |  |  | 49.6% Etomidate; 63.1% Succinylcholine |  |  |  |  |  | SBP <90mmHg or mean BP <65mmHg or requiring vasopressor administration or an increased dose of vasopressor or a decrease in BP by >20%, using the lowest vital signs during 30 min after intubation |
|  | Hwang, Park, et al., 2018 | EM physicians - approximately 50% by PGY1 or 2 | Respiratory distress 35% | 69% in first year, 79% in third year | VL 9% in first year, 60% in in third year, | 84% RSI rate; 48% Etomidate; 62% Succinyl- choline; 87% NMB used | 8% |  | Placement of a laryngoscope blade into the mouth, regardless of an endotracheal tube insertion attempt | Successful ETI on the first intubation attempt. | SpO2 <80% at any time within 30 mins following intubation | Systolic blood pressure of less than 90 mm Hg at any time within 30 minutes after intubation |
|  | Hwang, Lee, et al., 2018 | EM residents only - all others excluded. 51% by junior residents (PGY1 or 2) | Respiratory distress 36% | Overall: 72%. 79% (C-MAC), 65% (DL) | DL only | 38% No sedative use was most common. 45% Succinylcholine | 11% | Esophageal intubation 6% | Placement of a laryngoscope blade into the mouth regardless of successful tube insertion into the trachea | Successful ETI on the first intubation attempt | Peripheral oxygen saturation less than 80% at any time during the 30 minutes following intubation | SBP < 90mmHg at any time during 30 minutes following intubation |
|  | Hwang and Joon, 2010 |  | "Failure of ventilation or oxygenation" 83% |  |  |  |  |  |  |  |  |  |
| Singapore General Hospital Emergency Airway Registry | Weng et al., 2021 | 98.6% EM physicians. 60% of these by EM attendings | Medical: 86.4% Attending; 91.5% Non- attending | Overall: 77.5%. EM attendings: 84.2%; EM non-attendings: 67.4% | DL 87.3% |  | 12.9%; 10.9% EM attendings; 15.9% EM residents |  | Attempt to pass the endotracheal tube (ETT) through the vocal cords |  |  |  |
|  | Zakaria and Wong, 2017 | EM residents, EM attendings -  % not reported |  | 77.5% |  |  |  |  |  |  |  |  |
|  | Wong and Ngo, 2009 |  |  |  |  |  |  |  |  |  |  |  |
|  | Wong and Ng, 2008 | 91.3% EM physicians | Cardiac arrest 39.1% |  | DL 54.8% | 37.60% |  |  |  |  |  |  |
|  | Wong and Ho, 2006 | EM attendings, anaesthesia attending, EM resident, medical officer, surgery attending  -% not reported | Medical 86.4%- medical arrest 37% | EM physician 53.7% - 81.3% |  | 38.80% | Overall rate 9.8% 10.5% (pre- SARS); 10.5% (during SARS); 9.6% (post- SARS) |  |  |  |  |  |
| South African ED Registry | Hart and Goldstein, 2020 | EM physicians, EM residents, medical officers, interns, paramedics, nursing staff. EM trainees most often - % not reported | Medical 71.9% - pulmonary causes 20.3% | Overall 77.7%;  73.3% (DL), 81.7% (VL). | VL 52% | Etomidate 46.8% | 33% | Hypoxia 16.2% |  | Successful intubation by the first operator on the first attempt | SpO2 <90% | SBP <90mmHg or 20% change from baseline |
| The Aberdeen Royal Infirmary Airway Registry | Yeap et al., 2019 | EM, anaesthesia, ITU doctors - % not reported |  | 79.2% (bougie), 82.5% (no bougie); 81.1% (EM intubators), 86.7% (Anaesthetics), 66.7% (ITU doctors) |  |  | 13% | Hypoxia: Bougie 15.1%; No bougie 7.5% |  |  |  |  |
| The Alfred Airway Registry | Groombridge et al., 2021 | During covid more likely to be senior intubator (55.9%, vs pre-covid 22.6%). Most common pre-covid was ED resident 63.5% | Medical: 56.4% Pre-covid; 52.9% during covid | 93.8% pre-covid;, 95.6% during covid | Pre covid: VL 82.5%; During covid: VL 95.6% | 66.9% Ketamine during covid; 42.3% Ketamine pre-covid; 86.8%, Rocuronium during covid; 52.1% pre-Covid | 20.4% Pre covid; 34.6% during covid | Hypoxia: 9.6% pre-covid; 18.4% during covid | Single passage of the laryngoscope blade into the mouth, |  | SpO2 <93% | Administration of IV fluid or vasopressor |
|  | Groombridge et al., 2020 | ED residents 63.4% | Medical 56.3% | 81.2% pre- intervention  94.6% post- intervention | DL 66.3% | Ketamine 34.6%; Succinylcholine 62.7% | 27.4% pre- intervention  23.6% post- intervention |  |  |  | SpO2 <93% | Administration of IV fluid or vasopressor |
| The Royal North Shore Emergency Airway Registry | Fogg et al., 2016 |  | Medical more common - % not provided, 15-20% Overdose /ingestion | 83.4% pre- intervention  93.9% post- intervention | VL increased from 48.8% to 92.7% throughout study |  | Total: 29.0% pre- intervention  19.4% post-intervention | Hypoxia 10.9% |  |  | SpO2 <93% |  |
|  | Vassiliadis et al., 2015 | EM consultants, EM registrars, EM senior resident medical officers - % not reported | Medical  70% | Overall 83.5%; 85% C-MAC VL; 81.6%; DL | VL 57%, DL 43% | 91% received sedatives; 92% received paralytics | Total: 38.5% C-MAC; 32.7% DL; Overall 15% | Hypoxia 9.5% | Single passage of the laryngoscope blade past the lips. | Correct placement of the endotracheal tube into the trachea on first attempt.. This was confirmed by end tidal capnography in all cases, on the first attempt | SpO2 <93% | Requiring IV fluids |
|  | Annesley et al., 2012 | EM residents 57.5% of first attempts |  | 83.70% | VL 47.5% |  | 28% |  |  |  |  |  |

**ANZEDAR** The Australian and New Zealand Emergency Department Airway Registry, **BCARE** British Columbia Airway Registry for Emergencies, **DREAM** Defense Registry for Emergency Airway Management, **EDIR** Emergency Department Intubation Registry, **JEAN** Japanese Emergency Airway Network Registry 1 and 2, **KEAMR** Korean Emergency Airway Management Registry, **NEAR** National Emergency Airway Registry, **NERAA** National Emergency Resuscitation Airway Audit

Direct laryngoscopy (DL), video laryngoscopy (VL), GlideScope video laryngoscope (GVL) emergency department (ED), emergency medicine (EM), first pass success (FPS), rapid sequence intubation (RSI), systolic blood pressure (SBP), oxygen saturation (SpO2), intravenous (IV), neuromuscular blockade (NMB)
